# Probing inter-areal computations with a two-photon holographic mesoscope

**DOI:** 10.1038/s41593-026-02350-9

**Published:** 2026-08-03

**Authors:** Lamiae Abdeladim, Uday K. Jagadisan, Hyeyoung Shin, Mora B. Ogando, Hillel Adesnik

**Affiliations:** 1 Department of Neuroscience, University of California, Berkeley, CA USA; 2 Department of Molecular and Cell Biology, University of California, Berkeley, CA USA; 3 School of Biological Sciences, Seoul National University, Seoul, Republic of Korea; 4 The Helen Wills Neuroscience Institute, University of California, Berkeley, CA USA

**Keywords:** Neural circuits, Sensory processing

## Abstract

Brain computation depends on intricately connected yet highly distributed neural networks. Owing to the absence of the requisite technologies, causally testing fundamental hypotheses on inter-areal processing has remained largely out of reach. Here, we developed a two-photon holographic mesoscope capable of simultaneously reading and writing neural activity patterns with near-single-cell resolution across large regions of the mouse cortex. We demonstrate the precise photoactivation of spatial and temporal sequences of neurons in one or multiple cortical areas while reading out the downstream effects in several other regions. Thus, we have established mesoscale two-photon holographic optogenetics as a platform for mapping functional connectivity and causal interactions across distributed cortical areas with high resolution.

## Abstract (web summary)

The authors developed a new microscope that can measure and manipulate brain activity across multiple brain regions simultaneously with high precision, enabling scientists to study how spatially distributed brain circuits communicate and process information.

## Main

The neural computations that support sensation, cognition and behavior depend on the precise coordination of neural activity patterns within and across widely separated brain areas. Recent advances in optogenetics and three-dimensional (3D) light patterning^1,2^ have enabled investigators to causally probe how the activity of specific neural ensembles impacts computation and drives behavior^3–9^, but only in very small, circumscribed regions of the brain (less than 1–2 mm^2^). These techniques rely on phase modulation of the optical wavefront with a spatial light modulator (SLM), allowing the user to selectively target and photo-stimulate neural ensembles of interest. Despite the power of these new read–write optogenetic approaches, the inability to apply them to distributed brain networks has prevented investigators from causally probing the logic and principles of inter-areal communication that are central to brain function. A new technology that could overcome this technical barrier would allow neuroscientists to address key outstanding questions for the first time, which could have profound importance for understanding brain function in health, disease and neural development.

The recent introduction of mesoscale two-photon (2p) microscopes, which can sample neural activity with near-micron precision across up to 25 mm^2^ of brain tissue, has vastly increased the ability of investigators to acquire physiological data on distributed neural populations in behaving animals^10–16^. Although these 2p mesoscopes can monitor cellular activity, they have no ability for spatially targeted perturbations, preventing the user from probing causal relationships between the activity of specific neural ensembles in connected brain areas and behavior. Therefore, if it were possible to develop a 2p mesoscope that can not only measure but also manipulate neural activity with cellular-scale resolution, neuroscientists could use such a system to address longstanding mysteries of long-range neural communication in the brain. In practice, such a system would enable targeted perturbations of selected individual neurons or groups of neurons while simultaneously recording thousands of cells across cortical areas.

However, there are multiple substantial technical challenges to achieving high-resolution holographic optogenetics in a 2p mesoscope. Existing commercial 2p mesoscopes were not designed for the integration of holographic systems, requiring a large redesign of the mesoscope build. A particularly outstanding challenge in achieving high-resolution patterned photostimulation across a mesoscopic field-of-view (FOV) is the physical limits imposed by SLM designs, which ultimately constrain the accessible photostimulation FOV. We first designed and optimized a flexible 3D holographic system fully integrated onto a 2p random-access mesoscope (2p-RAM) that enables near-single-cell resolution holographic photostimulation of neural ensembles in the brain across a millimetric FOV. We tested and validated the optical capabilities of this new read–write platform to measure and recreate highly specific patterns of activity in the brain. In particular, we showed that we can decode the identity of the specific photostimulus purely from the modulation of activity of postsynaptic neurons in downstream areas, and that we can holographically recreate and transmit visual-like information across areas in mouse visual cortex. Next, we expanded the photostimulation FOV by an order of magnitude by integrating a random-access scan module into the 2p holographic path. We further showed how one can use this system to probe functional interactions between distant brain regions. Finally, we demonstrated, for the first time to our knowledge, near-simultaneous photostimulation of specific neural ensembles across distinct remote cortical areas. Together, these data illustrate how our 2p holographic mesoscope enables the user to probe causality at both the local and the inter-areal level with near-single-cell resolution.

## Results

### A 2p-RAM with temporally focused 2p holographic illumination

**Fig. 1:**
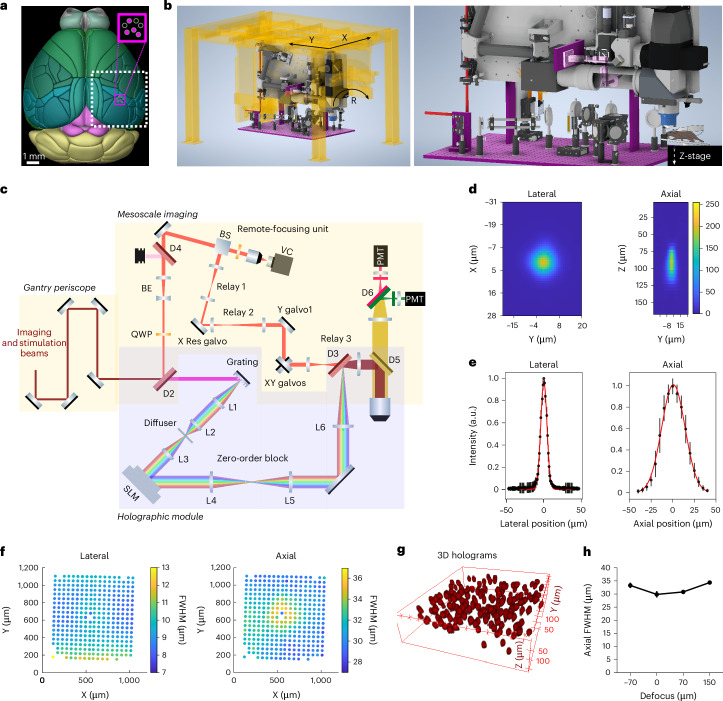
A 2p-RAM with temporally focused 2p holographic photostimulation. **a**, Schematic dorsal view of the mouse brain with putative functional areas demarcated (image credit, Allen Institute for Brain Science, Allen Brain Explorer 2 (ref. ^53^)). The holographic mesoscope allows targeted photostimulation in a 1 × 1 mm FOV (magenta square) combined with simultaneous mesoscale imaging across a nominal 5 × 5 mm imaging FOV (dotted white square). **b**, Left, CAD model of the holographic mesoscope platform. Right, blow-up of the holographic module attached to the main frame of the mesoscope. The 3D-SHOT holographic module consists primarily of the highlighted magenta parts and the optics on the highlighted extension breadboard. **c**, Simplified schematic of the optical path. QWP: quarter-wave plate, BE: beam expander, L*n*: achromatic doublet, D*n*: dichroic mirror; BS: polarization beamsplitter; VC: voice coil. **d**, Example lateral and axial 2p fluorescence intensity profiles of a single, temporally focused holographic illumination spot. **e**, Optical lateral and axial point-spread functions. Error bars, s.e.m. Red curve, Gaussian fit; lateral full-width-half-maximum (FWHM), 9 µm; axial FWHM, 32 µm; *n* = 551 holograms. a.u., arbitrary units. **f**, 2D grid of single-spot holograms (*n* = 538) showing the extent of the holographic FOV and the distribution of lateral and axial resolution across the FOV. **g**, Example point-cloud 3D holograms. **h**, Axial point-spread function as a function of digital *Z*-defocus. *n* = 38, 38, 38 and 37 holograms for −70 µm, 0 µm, 70 µm and 150 µm defocus, respectively. Error bars, s.d. Source data

**Extended Data Fig. 1:**
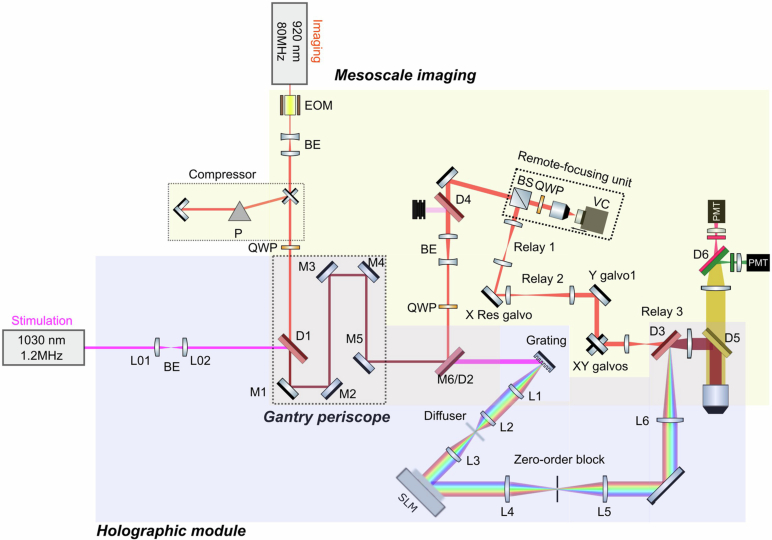
3D-SHOT two-photon holographic mesoscope optical setup. EOM: electro-optic modulator, BE: beam expander, P: prism, QWP: quarter waveplate, Dn: dichroic mirror, BS: polarization beamsplitter, VC: voice coil, SLM: spatial light modulator. M6 mirror in the original mesoscope design has been replaced with the D2 dichroic. Implementation in Figs. 1, 2, 3.

We designed a mesoscale all-optical read–write platform around a 2p-RAM system^10^ (Fig. 1a–c and Extended Data Fig. 1) that is commercially available (Thorlabs). The key features of this imaging system that make it a powerful tool for studying inter-areal communication are its nominal 5 × 5 mm FOV, a fast remote-focusing system for FOV curvature correction and multi-plane imaging, and four automated axes of motion for positional flexibility (Fig. 1c). We sought to develop a mesoscale holographic optogenetics system that achieves near-cellular resolution photostimulation despite the lower numerical aperture of the optical path and that could likewise address the imaging plane curvature. Furthermore, we aimed for an optical design that does not compromise the four-axis movement capabilities of the 2p-RAM system, given that they are essential for executing a diverse array of neurobiological experiments. We therefore designed a compact and re-dimensioned 3D holographic module that uses temporal focusing to confine excitation axially (three-dimensional sparse holographic optogenetics with temporal focusing; 3D-SHOT^17,18^). To preserve all translational axes of the entire system, we built the holographic module on an extension breadboard attached to the movable main frame of the microscope (Fig. 1b, Extended Data Fig. 1, Supplementary Note 1 and Supplementary Table 1). In 3D-SHOT, an initial blazed grating generates a temporally focused disc of light that is magnified and replicated in multiple locations within a 3D volume by an SLM. The 3D light pattern is then relayed onto the objective image space through a set of 4*f*-systems (Fig. 1c). Given the large distance on the mesoscope between the photostimulation laser output and the 3D-SHOT module, we added a non-magnifying telescope after the laser to control the divergence of the beam impinging on the diffraction grating. Preserving the alignment invariance of the holographic path with the motorized movements of the mesoscope is a key challenge. To address this issue (Fig. 1c), we first combined the imaging and photostimulation beams before entering the multi-stage periscope so that the photostimulation beam maintains invariance with X and Y frame translations. Second, we replaced the last periscope mirror with a long-pass dichroic that directs the imaging beam (920 nm) onto the vertical breadboard and the photostimulation beam (1,030 nm) onto the extension breadboard so that the latter maintains invariance with rotation. Third, we recombined both beams with a short-pass dichroic placed right before the mesoscope tube lens on the vertical breadboard. Consequently, the holographic module is invariant within all dimensions controlled by the motorized microscope frame, X, Y translations and rotation. We recovered axial translation by adding a motorized stage for animal positioning along the vertical axis, additionally mounted on a goniometer to ensure that imaging and photostimulation are fully orthogonal to the cranial window surface.

To quantify the optical resolution of the mesoscale holographic system, we first measured the spatial profile or point-spread function (PSF) of single 2p excitation spots (Fig. 1d,e,f), which measured 9 ± 0.7 μm in the lateral dimension and 32 ± 1.6 μm in the axial dimension (mean ± s.d; *n* = 538 holograms arranged in a 2D grid; see also Fig. 1f and Supplementary Fig. 1). Although these values are slightly larger than those previously reported for 3D-SHOT in a conventional 2p microscope, they are consistent with the lower NA of the mesoscope objective (Supplementary Note 2). We then determined the extent of our holographic FOV, which is equivalent to the total accessible FOV range of the SLM at the system’s magnification. We set the FOV limits to the farthest accessible holograms without aliasing, corresponding to a total lateral FOV of ~950 × 990 μm (Fig. 1f), which closely approximates the theoretical SLM FOV at this magnification (see Methods). We compensate for the inherent decrease in diffraction efficiency laterally across the FOV by adjusting the power delivered to each spot according to its spatial location (Supplementary Fig. 1). Notably, we observed that although holograms farther from the FOV center had expectedly lower intensity profiles (owing to lower diffraction efficiencies), off-axis holograms remained only slightly affected by off-axis aberrations (Supplementary Fig. 2). Next, we generated multiple holograms spatially arranged in a 3D point-cloud (Fig. 1g). We then measured the axial optical PSFs in 3D and found that they were mostly invariant within a digital defocusing range of 220 µm (Fig. 1h). A potential challenge for holographic targeting on the 2p-RAM mesoscope is the large microscope field curvature, 160 μm over the 5 mm FOV, mostly introduced by the mesoscope objective. Hence, we quantified the flatness of our holographic FOV by measuring the axial locations of holograms generated with equi-Z SLM coordinates and found the maximal peak-to-valley to be ~40 µm, which is well within the addressable defocus range of the SLM and can be corrected for without compromising 3D targeting (Supplementary Fig. 1). Another potential challenge for multiphoton holographic optogenetics on the 2p-RAM mesoscope is the introduced dispersion from the 4*f* lenses and the large mesoscope optics. We estimated the total group delay dispersion introduced by the holographic path to be ~15,000 fs^2^ (Supplementary Table 2). For relatively broad pulses commonly used in multiphoton optogenetics (~400 fs), the broadening of the pulse width at the objective focus is minimal, thereby eliminating the need for a pulse compression unit on the holographic path.

**Extended Data Fig. 2:**
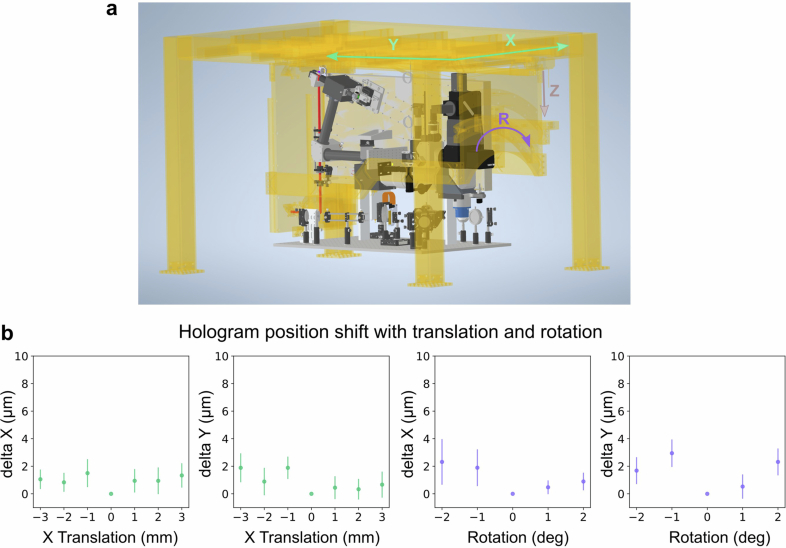
Targeting invariance with 2P-RAM mesoscope movement. **a**) The microscope main frame is motorized in X, Y translation and rotation. The holographic module was designed to be solidary to the main frame thus preserving motion along these axes. Axial Z translation in the 2P-RAM mesoscope is implemented by moving the vertical breadboard relative to the main frame. To maintain holographic invariance, that axis was locked and replaced by a motorized lab jack (MLJ150, Thorlabs) to translate the animal axially relative to the microscope. **b**) Targeting invariance measured for translation (n=18 spots) and rotation (n=19 spots) of the microscope around a central reference position. Delta X and Y are computed, respectively, as the difference between the X and Y location of the centroids of holographically burnt holes in a fluorescent slide at the translated or rotated position and the X and Y location of the centroids in the central reference position. Markers: mean value, error bars: standard deviation. Source data

Finally, we assessed holographic targeting stationarity with X, Y translations and rotation, which is essential for accurate optical generation of neural activity patterns despite movement of the mesoscope relative to the brain. We found that the error between the original hologram location and displaced stage positions remained less than 3 µm over a ±3 mm translation range and a ±2.5° rotation angle range, demonstrating alignment invariance with mesoscope frame movements (Extended Data Fig. 2).

### In vivo 2p photostimulation on the 2p-RAM

**Fig. 2:**
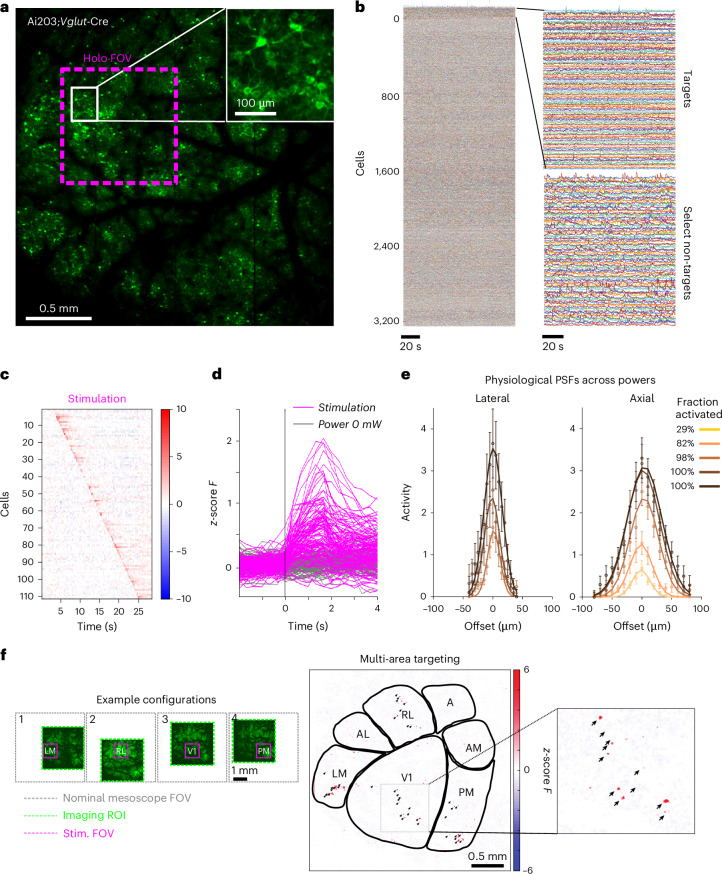
2p holographic optogenetics at the mesoscale. **a**, A 2p mesoscale image of widespread co-expression of GCaMP7s and ChroME over a large FOV in a *Vglut1*-Cre; Ai203 transgenic mouse. The magenta square indicates the accessible holographic FOV within an example mesoscale imaging FOV of 2.4 mm × 2.4 mm recorded at a 4.5 Hz frame rate. **b**, Example single-trial calcium traces from a set of photostimulation targets within the holographic FOV and thousands of traces from non-targets recorded across the mesoscale FOV. **c**, Calcium responses of targeted cells during single-cell sequential targeting on stimulation trials (right). Photostimulation with five pulses (5 ms, 30 Hz, 75 mW). Traces averaged over five trials. **d**, Stimulus-aligned target cell responses across the holographic FOV (*n* = 111 cells). Single-trial traces. **e**, In vivo optically measured lateral (left, *n* = 20 cells) and axial (right, *n* = 32 cells) physiological point-spread functions as a function of stimulation power. The *y* axis indicates neural activity measured as Δ*F* (see Methods). Absolute powers used are 0.5 mW, 1 mW, 2 mW, 4 mW and 8 mW in a *CaMK2*-tTA; tetO-GCaMP6s mouse, corresponding to a fraction of activated cells among targeted cells of 29%, 82%, 98%, 100% and 100%, respectively (see Extended Data Fig. 4). The filled circles represent the actual means across cells; error bars, s.e.m.; the solid traces are weighted Gaussian fits. For lateral PPSFs, fitted FWHMs are 28.5 µm, 31.9 µm and 38.4 µm at 2 mW, 4 mW and 8 mW, respectively. For axial PPSFs, fitted FWHMs are 35.4 µm, 46 µm, 59.8 µm, 66.4 µm and 70.4 µm at 0.5 mW, 1 mW, 2 mW, 4 mW and 8 mW, respectively. **f**, Demonstration of sequential area mesoscale holographic stimulation. Left, four example configurations to holographically target four different visual areas within the same session and record from surrounding visual areas. Imaging ROIs were selected for a 3 Hz imaging rate at 1 µm per pixel. Photostimulation with a single 250 ms pulse, 80 mW. Center, activation map of holographically targeted neurons in four visual areas (V1, PM, RL and LM). Black arrows point to target locations. Right, zoomed-in inset of the activation map in V1. Source data

**Extended Data Fig. 3:**
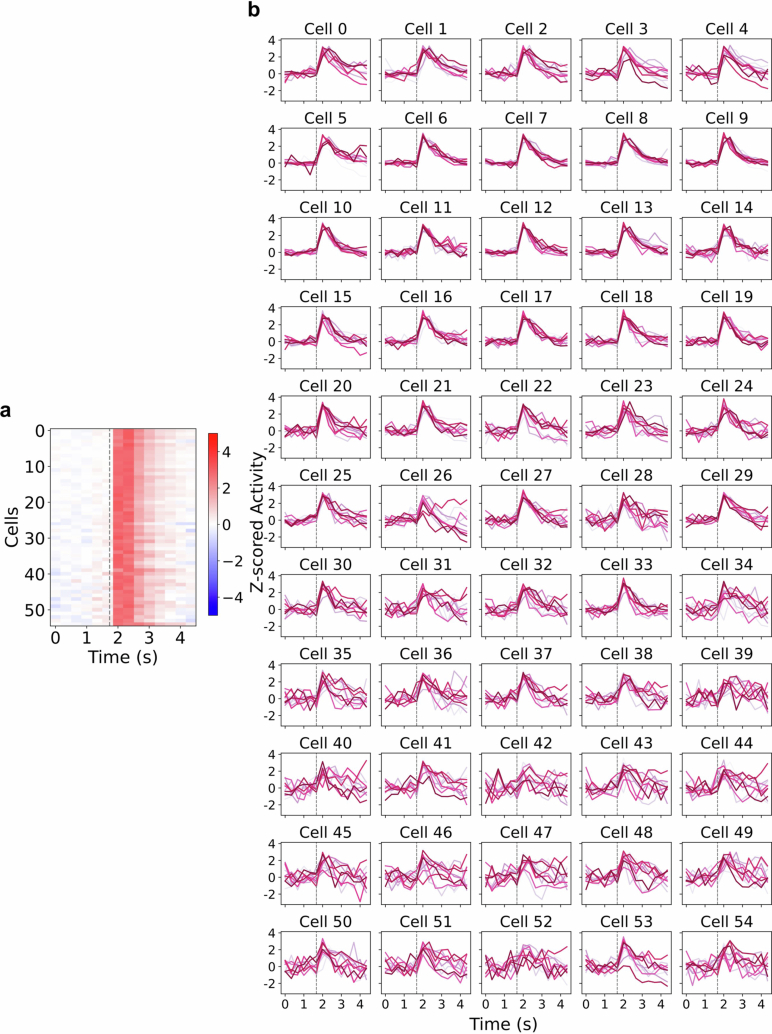
Reliable photostimulation of a large neural ensemble. **a**) Trial-average (n= 20 trials) post-stimulus time histogram (PSTH) of target neurons (n=55) simultaneously photostimulated. Dashed line indicates photostimulation start time. All neurons co-express GCaMP6s and ChRmine. **b**) Single-trial traces of individual target cells from the photostimulated neural ensemble. Source data

**Extended Data Fig. 4:**
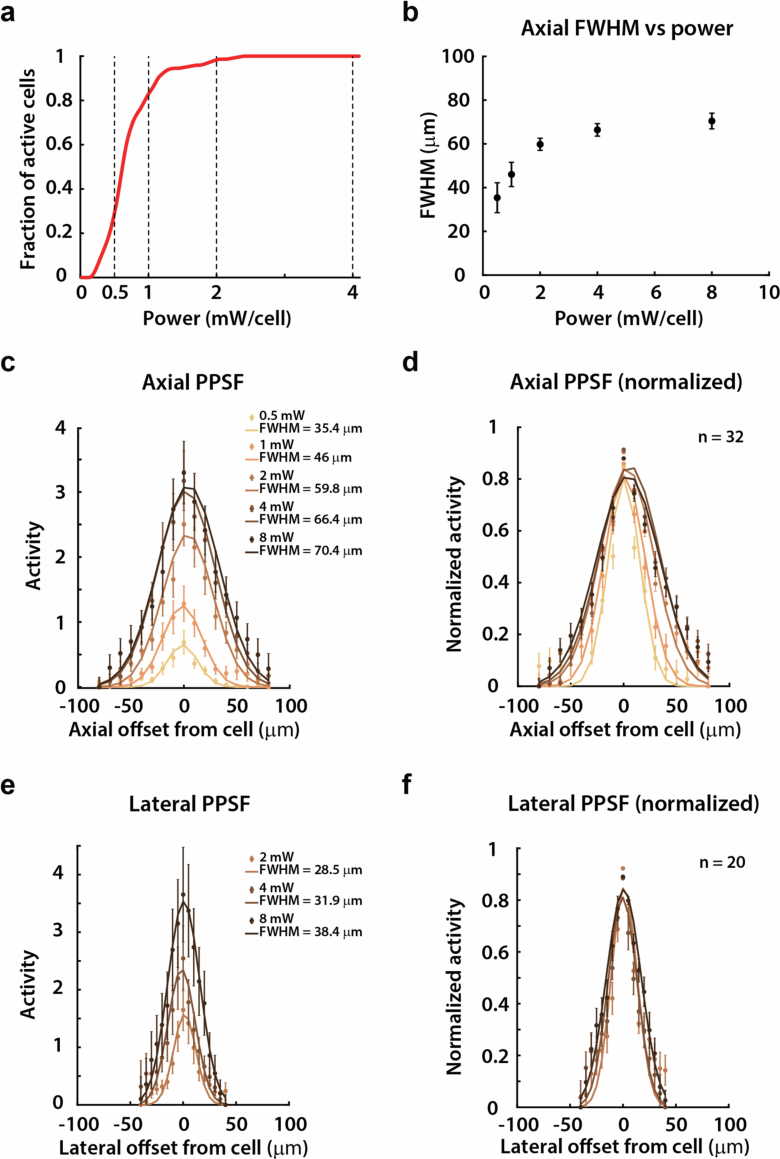
Physiological point-spread functions (PPSFs) as a function of power. **a**) Cumulative density histogram of the fraction of active cells used in the PPSF experiments (ordinate) that were active above a certain power (abscissa). **b**) Mean +/- s.e.m. axial FWHM across cells as a function of power. N = 32 cells. **c**) Axial PPSF profiles for each power condition. The filled circles and error bars are the actual mean +/- s.e.m. across cells, and the solid traces are weighted gaussian fits. N=32 cells. **d**) Normalized axial PPSF profiles for each power condition, normalized to the centered hologram location (axial offset = 0). N=32 cells. The filled circles and error bars are the actual mean +/- s.e.m. across cells, and the solid traces are weighted gaussian fits. **e**) Similar to c), but for lateral PPSFs. N = 20 cells. The filled circles and error bars are the actual mean +/- s.e.m. across cells, and the solid traces are weighted gaussian fits. **f**) Similar to d), but for normalized lateral PPSFs. All PPSF experiments were done in CaMK2-tTA;tetO-GCaMP6s mice injected with a pAAV-hSyn-GCaMP6m-p2A-ChRmine-Kv2.1-WPRE virus at discrete locations across visual cortex. N=20 cells. The filled circles and error bars are the actual mean +/- s.e.m. across cells, and the solid traces are weighted gaussian fits. Source data

Next, we tested our system for in vivo all-optical control of neural ensembles in awake mice. We tested the system on mice expressing soma-targeted opsin, either transgenically or by viral injection. In *Vglut1*-Cre; Ai203 mice^19^, which express the potent opsin ChroME and the sensitive calcium indicator (GCaMP7s) in cortical excitatory neurons (Fig. 2a), we were able to photo-activate selected individual cells within the holographic FOV while recording from thousands of neurons within the mesoscale imaging FOV (Fig. 2b–d). Sequential photostimulation at 75 mW per spot (10 × 10 ms pulses at 50 Hz) elicited significant time-locked responses in 70% of targeted cells (Fig. 2c,d and Supplementary Fig. 3). This finding demonstrates effective holographic photostimulation despite the slightly larger optical axial resolution. Next, using viral transduction with a soma-targeted ChRmine-expressing adeno-associated virus (AAV), we further demonstrated that we could reliably and simultaneously photo-activate large groups of neurons (Extended Data Fig. 3 and Supplementary Fig. 4). To carefully quantify the effective spatial resolution of holographic photostimulation, we recorded physiological PSFs (PPSFs) all optically in awake animals in a wide range of conditions (Fig. 2e, Extended Data Fig. 4 and Supplementary Figs. 5 and 6). PPSFs can vary wildly depending on multiple parameters, both optical and linked to the preparation (see Supplementary Note 3). We measured lateral PPSFs between 23 µm and 38 µm across conditions (*n* = 23 cells, pooled across ChroME and ChRmine cells), and axial PPSFs between 35 µm and 77 µm across conditions (*n* = 35 cells, pooled across ChroME and ChRmine cells). This indicates that despite the lower NA inherent to the mesoscope objective, the achieved effective axial resolution in vivo is within the range of reported PPSFs in 2p optogenetics studies^9,20,21^ and that under certain conditions, near-single-cell resolution can be achieved with our system (Extended Data Fig. 4 and Supplementary Fig. 5).

To illustrate how one can use the 2p holographic mesoscope to address key questions related to inter-areal processing despite the holographic FOV limitation, we sequentially targeted ensembles of neurons in different visual areas while recording the same set of visual cortical areas by translation of the mesoscope, taking advantage of the system’s targeting invariance to spatial translation. We could thus sequentially activate neural ensembles from four different cortical areas (primary visual cortex (V1), posteromedial area (PM), rostrolateral area (RL) and lateromedial area (LM)) while simultaneously recording activity from six surrounding visual areas (Fig. 2f). In each of the four configurations, the system was translated to match the target stimulation area to the holographic FOV. The imaging FOV was then flexibly selected within the accessible 5 mm × 5 mm FOV to maintain a consistent FOV across the four configurations, as shown in Fig. 2f by the similarity in vasculature patterns between the four configurations. Photostimulation in each cortical area elicited a robust response in the holographically targeted cells, even while we maintained acquisition of physiological data from the entire FOV. This demonstrates that a 3D-SHOT-based holographic mesoscope can achieve, in the same mouse, ensemble stimulation in any chosen visual area while concurrently recording neural activity from thousands of neurons in surrounding visual areas.

### Specific transmission of information across cortical areas

**Fig. 3:**
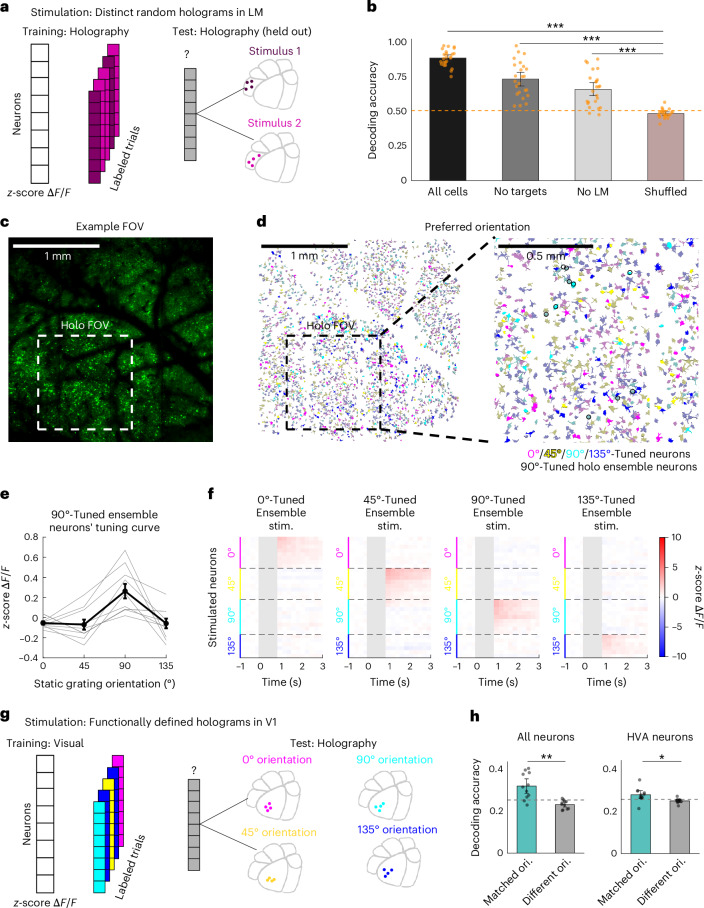
Specific transmission of information at the mesoscale upon targeted local 2p photostimulation. **a**, Schematic of a decoder trained to discriminate different holographically induced patterns of activity from downstream responses. Multi-target (3–20 cells) stimulation in LM (*n* = 4 sessions, two mice expressing GCaMP7s and ChroME), with 10 × 10 ms pulses at 16 Hz and 50 mW. Recording rate, 4.5 Hz. **b**, Decoding accuracy of a classifier trained to discriminate the holographically induced patterns in LM from different groups of non-targeted cells in the FOV. All cells: decoding from all neurons in the mesoscale FOV; no targets: decoding from all neurons except the targeted cells; no LM: decoding from all neurons outside the LM area; shuffled: decoding from all neurons outside the LM area, but with the training set labels shuffled. The cross-validated decoding accuracy across *n* = 4 sessions for all cells, no targets, no LM and shuffled categories was, respectively, 0.88 ± 0.01, 0.73 ± 0.03, 0.65 ± 0.02 and 0.48 ± 0.01 (mean ± s.e.m.), with the chance level of 0.5. Two-sided Wilcoxon signed-rank test across *n* = 4 sessions, *P* values: all cells, no targets: 4.89 × 10^−5^; all cells, no LM: 1.67 × 10^−6^; no targets, no LM: 0.011; all cells, shuffled: 1.67 × 10^−7^; no targets, shuffled: 1.32 × 10^−9^; no LM, shuffled: 5.96 × 10^−7^. All groups (all cells, no targets, no LM) show highly significant decoding performance (*P* < 0.001) compared to the shuffled group. **c**, Example imaging FOV used in this experiment (2.4 × 2.4 mm^2^ at 4.5 Hz recording rate, *Vglut1*-Cre; Ai203 mouse with neurons co-expressing GCaMP7s and ChroME). The holographic FOV was positioned over V1. **d**, Same FOV as **a**, with the cells color-coded by their preferred orientation (0/45/90/135° in magenta/yellow/cyan/blue, respectively). Significantly tuned cells are labeled with solid colors while other cells are grayed out (*P* < 0.05 two-sided Wilcoxon rank-sum test for preferred versus orthogonal orientations; *P* values for all neurons are included in Supplementary Data). Right, enlarged image of the holographic FOV with thin circles indicating the holographic targets that were activated by the 90°-tuned ensemble stimulation. **e**, Orientation tuning curves of a group of neurons that were then co-activated as the 90°-tuned ensemble stimulation in **f**. Photostimulation with ten pulses (10 ms, 16 Hz, 50 mW). Gray, individual neuron tuning curves. Black, mean ± s.e.m across *n* = 10 neurons. **f**, Holographically evoked activity in a population of neurons that were grouped into four distinct orientation-specific ensembles. Imaging frames recorded during the holographic stimulation are grayed out owing to the holographic stimulation artifact. **g**, Schematic of a decoder trained on visual trials and tested on holographic trials. **h**, Decoding performance in assigning holographic trials (functional-specific holographic perturbations) to matching visual trials (oriented gratings). Decoding accuracy was measured as the proportion of holographic trials that were classified as the grating orientation corresponding to the photostimulated ensemble’s orientation preference. Given that four orientations are probed, the chance level is at 0.25. Left, decoder trained and tested on all neurons in the FOV (mean ± s.e.m., 0.316 ± 0.0185 matched orientation, 0.228 ± 0.0062 different orientation, *P* = 0.0098 two-sided Wilcoxon signed-rank test across *n* = 11 sessions). Right, decoder trained and tested only on neurons in downstream extrastriate areas, excluding V1 (mean ± s.e.m., 0.271 ± 0.0096 matched orientation, 0.243 ± 0.0032 different orientation, *P* = 0.04 two-sided Wilcoxon signed-rank test across *n* = 11 sessions). HVA, higher visual area. Source data

One of the major advantages of mesoscale 2p holographic optogenetics should be the ability to specifically perturb the cortical network in one area and read out the impact of the perturbation in the population activity of thousands of neurons in multiple downstream areas, something not possible with conventional 2p microscopes. To test this possibility, we stimulated ensembles of neurons in visual area LM while recording from neurons across four to six other visual cortical areas in a total 2.4 mm × 2.4 mm FOV at 4.5 Hz (Fig. 3a,b). We then asked whether activating different neural activity patterns in one cortical area (LM) induces differentiable patterns of modulation in downstream areas. To answer this question, we sought to decode, trial by trial, the identity of the holographically induced pattern in LM from the neural responses in the other visual areas (Fig. 3a and Supplementary Fig. 7). Indeed, we could properly predict the holographic pattern on each trial significantly above chance levels, despite excluding all measurements taken from LM itself (Fig. 3b). This indicates that photostimulation of even relatively modestly sized neural ensembles is sufficient to drive distributed and informative patterns of neuromodulation downstream at the cellular scale.

**Extended Data Fig. 5:**
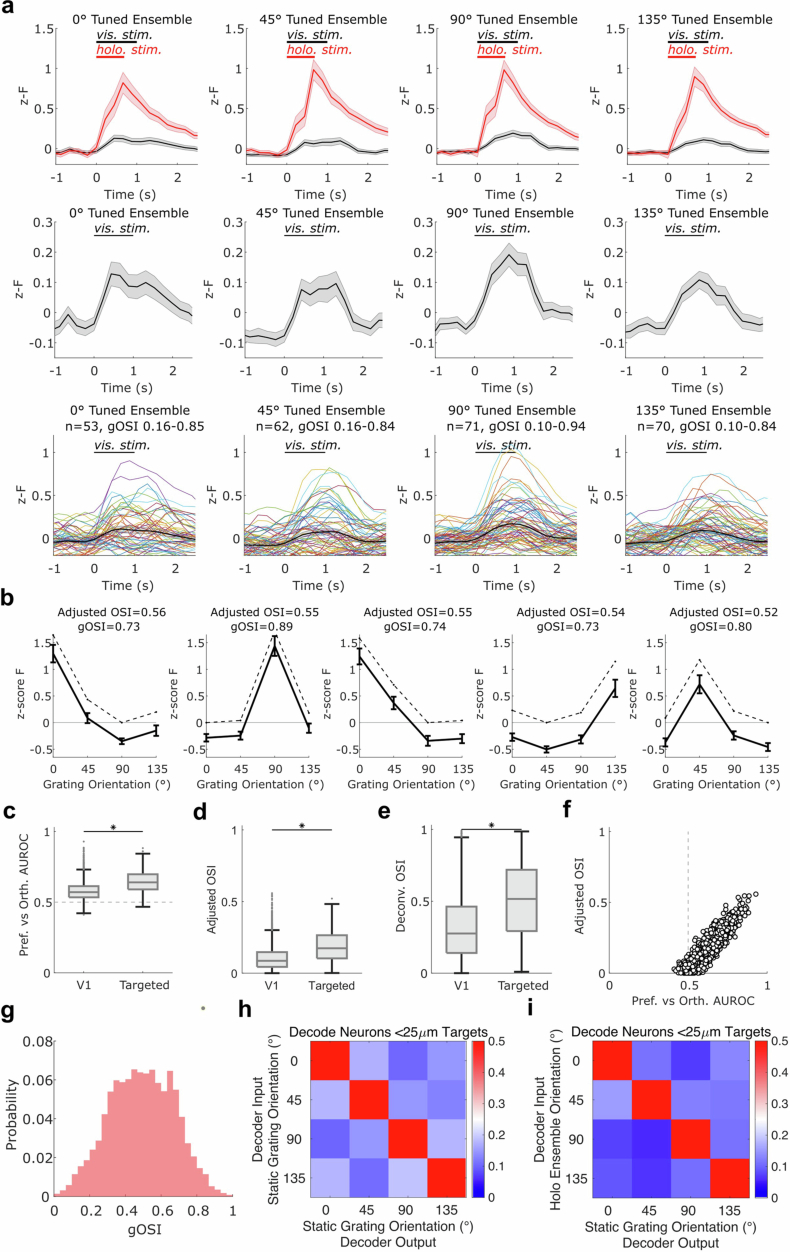
Stimulation of functionally specific neuronal ensembles sharing orientation preference in mouse visual cortex. **a**) Top: Visual (black) and holographic (red) responses of holographically targeted neurons in each ensemble are shown (mean ± SEM across neurons, n=53/62/71/70 neurons in 0/45/90/135° ensembles respectively, aggregated across 11 sessions from 2 mice). Visual responses were driven by static gratings in each orientation, presented for 1s with no intervening period. Holographic responses were driven by synchronous photostimulation of the targets, where each target received 10 of 10ms pulses at 16Hz and 50mW power. Center: Zoom on the visual responses. Bottom: Ensemble mean responses (black) with overlaid individual cell responses. Tuned ensembles contained both sharply tuned and weakly tuned neurons. **b**) Example of highly-tuned neurons. The error bar on the tuning curves indicates s.e.m across n=50 trials. Dashed curve corresponds to the shifted tuning curve used to calculate gOSI (see below). **c**) Preferred vs. orthogonal orientation AUROC for targeted neurons and other non-targets in V1. Pref. vs Orth. AUROC V1 (n=5152) vs Targeted (n=364). Two-sided Wilcoxon rank-sum test, p = 1.95 × 10^−54^. Box-and-whisker plots are formatted as follows: centerline, median; box limits, upper and lower quartiles; whiskers, 1.5× interquartile range; points, outliers. **d**) Adjusted orientation selectivity index (OSI) of targeted neurons and other non-target neurons in V1. Adjusted OSI is defined as Adjusted OSI is hence defined as OSI_adj_ = $\frac{{R}_{{adj\_pref}}-{R}_{{adj\_orth}}}{{R}_{{adj\_pref}}+{R}_{{adj\_prth}}}$ where R_adj_pref_ = R_pref_ +1 and R_adj_orth_ = R_orth_ + 1 (cf. Methods). Adjusted OSI V1 (n=5152) vs Targeted (n=364). Two-sided Wilcoxon rank-sum test, p = 4.56 × 10^−49^. Box-and-whisker plots are formatted as follows: centerline, median; box limits, upper and lower quartiles; whiskers, 1.5× interquartile range; points, outliers **e**) OSI measured with deconvolved responses (suite2P). Deconv. OSI V1 (n=5152) vs Targeted (n=364). Two-sided Wilcoxon rank-sum test, p = 6.82 × 10^−39^. Box-and-whisker plots are formatted as follows: centerline, median; box limits, upper and lower quartiles; whiskers, 1.5× interquartile range; points, outliers **f**) Adjusted OSI vs. AUROC across V1 neurons, n = 5152, Pearson corr. R = 0.896, Spearman corr. Rho = 0.873. **g**) Global orientation selectivity index (gOSI), which is defined as follows:${gOSI}=\frac{\sum {R}_{\theta }{e}^{2i\theta }}{{R}_{\theta }}$. Because gOSI is intended to be applied to non-negative activity metrics, gOSI was calculated from shifted tuning curves where the mean response to the least preferred orientation was subtracted. **h**) Classification accuracy of a linear support vector machine (SVM) trained to classify visual response of 4 orientations, for neurons within 25 ${\rm{\mu }}{\rm{m}}$ of each target in holographic ensembles (average 77.7 neurons per session). 10-fold cross-validated decoding accuracy was 0.557 ± 0.019 mean ± standard error mean (SEM) across N=11 sessions (${\rm{p}}=9.77\times {10}^{-4}$ two-sided Wilcoxon signed-rank test compared to chance-performance of 0.25). **i**) Classification accuracy of the same SVM when probed with orientation-specific holographically evoked activity without any visual stimulus (0.696 ± 0.043 mean ± SEM, ${\rm{p}}=9.77\times {10}^{-4}$ two-sided Wilcoxon signed-rank test across N=11 sessions). Decoding accuracy was measured as the proportion of holography trials that were classified as the corresponding grating orientation. Source data

A key benefit of 2p holographic optogenetics is the ability to co-activate ensembles of neurons based on their functional properties, which may be critical for inter-area communication. We therefore asked whether we could transmit specific visual-like information across cortical areas by co-activating groups of visual cortical neurons that share orientation preference (Fig. 3c–h). In the mouse visual cortex, orientation preference shows minimal spatial clustering^22–24^ and therefore co-activating an ensemble of co-tuned neurons requires highly precise optical targeting. To test this capability, we measured orientation tuning curves of V1 neurons and then constructed various ensembles of neurons with shared orientation preference for 2p holographic stimulation (Fig. 3d,e). Indeed, we could successfully photo-activate groups of co-tuned neurons in V1 (Fig. 3f and Extended Data Fig. 5) while recording activity from surrounding areas. To test whether our holographic stimulation generated functionally specific patterns of neural activity, we trained a decoder to discriminate the visual responses evoked by static gratings across four orientations (Fig. 3g and Extended Data Fig. 5). In the test phase, we probed this decoder, trained only on visual responses, to classify neural activity patterns evoked purely by photostimulation of co-tuned ensembles; that is, in the absence of any visual stimulus. The decoder was able to classify holographic photostimuli above chance across both photostimulation targets and across all recorded neurons (Extended Data Fig. 5 and Fig. 3h). Importantly, even when only decoding from neurons in downstream extrastriate areas, the decoder correctly classified holographic trials according to the orientation preference of the photostimulated ensemble, slightly but significantly above chance (Fig. 3h). This result demonstrates that the system both writes in and transmits visually relevant information across cortical areas. Although targeted activation often recruits additional off-target neurons, these results demonstrate that specific, functionally defined ensembles can still be preferentially enhanced to emulate visually induced neural activity.

### Photostimulation across a mesoscopic FOV with random-access 3D-SHOT

**Fig. 4:**
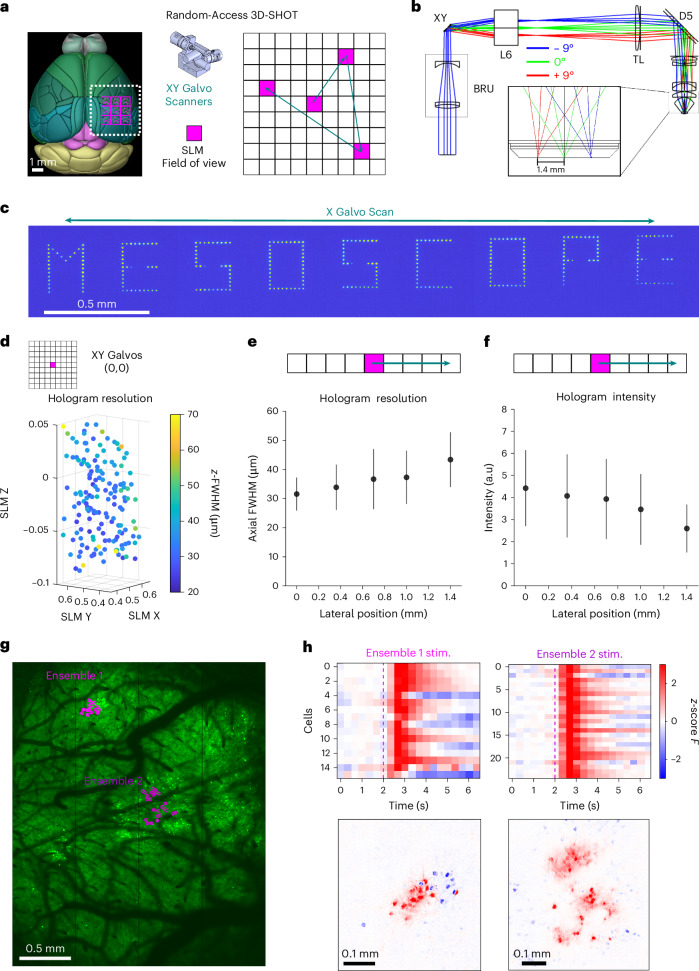
Mesoscale 2p optogenetics with random-access 3D-SHOT. **a**, Principle of random-access 3D-SHOT. XY Galvanometer scanners are used to deflect the center of the SLM FOV, thus effectively expanding the accessible holographic stimulation area. Mouse brain schematic adapted from Allen Brain Explorer 2 (ref. ^53^). **b**, Zemax optical model of the added scanning module implemented for FOV expansion. BRU, beam reducer unit. Compact Galilean telescope with a 2× beam reduction factor. XY, large-aperture 30 mm galvanometer scanners; L6, *f*-theta scan lens; ±9° optical deflection enables a ±1.4 mm shift of the center of the SLM FOV in each direction. **c**, Holographic multi-dot fluorescence patterns generated with random-access 3D-SHOT. Each letter was generated at a distinct X galvanometer position, ranging from –2 V (–9° optical deflection) to +2 V (+ 9°), in 0.5 V increments. Holographic letter patterns were recorded independently using a substage camera, which was translated by 350 µm between each recording. All patterns were stitched together to reconstruct the full 2.8 mm scan range. Occlusions at the center of targeted SLM FOVs correspond to the position of the zero-order block that is used to block non-diffracted light. **d**, Axial optical PSF measured on a 3D distribution of spots (*n* = 154) in the central holographic FOV (X galvo = 0°, Y galvo = 0°) within a 400 µm × 400 µm × 150 µm volume. Mean axial FWHM = 34 µm. **e**, Mean axial resolution measured across the 3D distribution of spots at different galvanometer scan positions. See Extended Data Fig. 7 for more extensive characterizations. Error bars, s.d.; *n* = 380, 176, 169, 142 and 114 holograms, respectively, for 0 mm, 0.35 mm, 0.7 mm, 1.05 mm and 1.4 mm galvo positions. **f**, Mean single-hologram intensity measured across the 3D distribution of spots at different galvanometer scan positions. See Extended Data Fig. 7 for more extensive characterizations. Error bars, s.d.; *n* = 380, 176, 169, 142 and 114 holograms, respectively, for 0 mm, 0.35 mm, 0.7 mm, 1.05 mm and 1.4 mm galvo positions. **g**, Mesoscopic 3.1 mm × 2.4 mm FOV of a *CaMK2*-tTA; tetO-GCaMP6s mouse visual cortex injected with a pAAV-hSyn-GCaMP6m-p2A-ChRmine-Kv2.1-WPRE virus at discrete locations. Photostimulation targets are segmented from the mesoscale FOV and assigned specific combinations of targeting phase masks and XY galvanometer positions (see Methods). **h**, Top, post-stimulus time histograms of activated neural ensembles represented in **g** and arbitrarily located in the mesoscopic FOV. Bottom, spatial fluorescence maps showing the mean pixel-by-pixel baseline-subtracted fluorescence intensity across 2.3 s (seven frames) post stimulation. Scale range, −2 × 10^−3^ (blue) to +2 × 10^−3^ (red). Targets are indicated with dark red circles. Gray stripe indicates exclusion area owing to photostimulation artefact (see Methods). Source data

**Extended Data Fig. 6:**
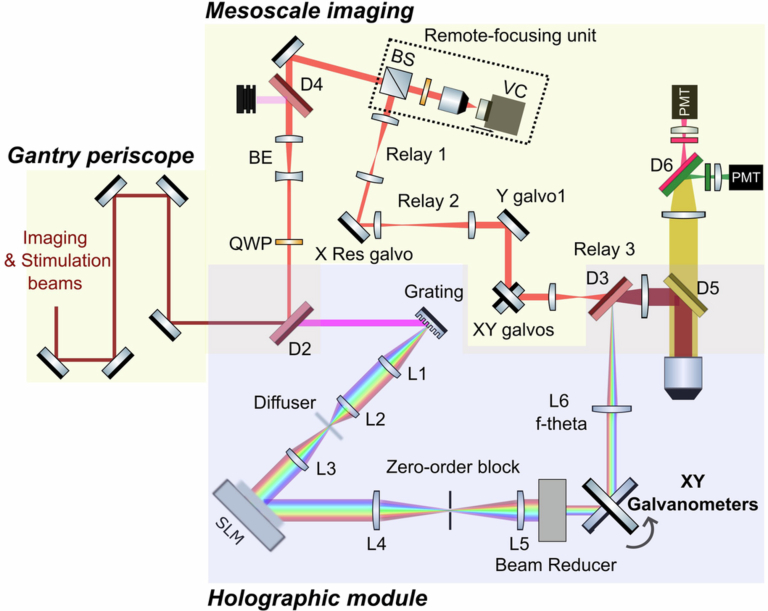
Random-access 3D-SHOT optical layout. A beam reducer and an XY galvo scanner pair are introduced in the collimated space between L5 and L6. L6 is replaced by an f-theta scan lens. The recombination dichroic D3 is replaced with a larger aperture mirror. Implementation in Figs. 4, 5, 6.

Activating neural ensembles in one area and recording from adjacent areas opens a new class of causal paradigms and is sufficient for many experiments. However, creating comprehensive inter-areal functional connectivity maps requires stimulating groups of neurons in different cortical areas within the same experiment, preferably rapidly and randomly interleaved across trials. The initial holographic mesoscope we described above does not achieve this objective, as the photostimulation area is fixed within the FOV owing to limitations of the SLM. To overcome this challenge, we sought to engineer a random-access holographic photostimulation path that would allow flexible jumps to distant neural targets. Our solution incorporated XY galvanometer scanners conjugated to the back aperture plane, enabling flexible and fast repositioning of the SLM FOV anywhere within the mesoscopic FOV (Fig. 4a). Implementing this solution using exclusively off-the-shelf optics proved challenging because of the limited availability of large-aperture components and the need to satisfy multiple design constraints simultaneously. First, the galvanometers needed to be conjugated to both the objective back aperture and the SLM. Second, because of the large beam size at the back aperture, even when oversized galvanometers were used, the beam still required significant reduction, and this beam reduction had to maintain the resolution and optical specifications of the 3D-SHOT path at the center of the FOV. Third, there were substantial steric constraints, given the use of large 3-inch optics within the compact space of the mesoscopic breadboard. Finally, the imaging and holographic paths had to be not too far from coplanarity to maintain the 3D capability of the system. We therefore designed an optical configuration that combined a compact pre-scanning beam reducer in collimated space, large 30 mm XY galvanometer scanners and an *f*-theta scan lens with a shortened focal length to restore the beam size at the back aperture (Fig. 4b and Extended Data Fig. 6). All optical components could be obtained as stock items from major optics vendors, avoiding the need for customized optical elements.

**Extended Data Fig. 7:**
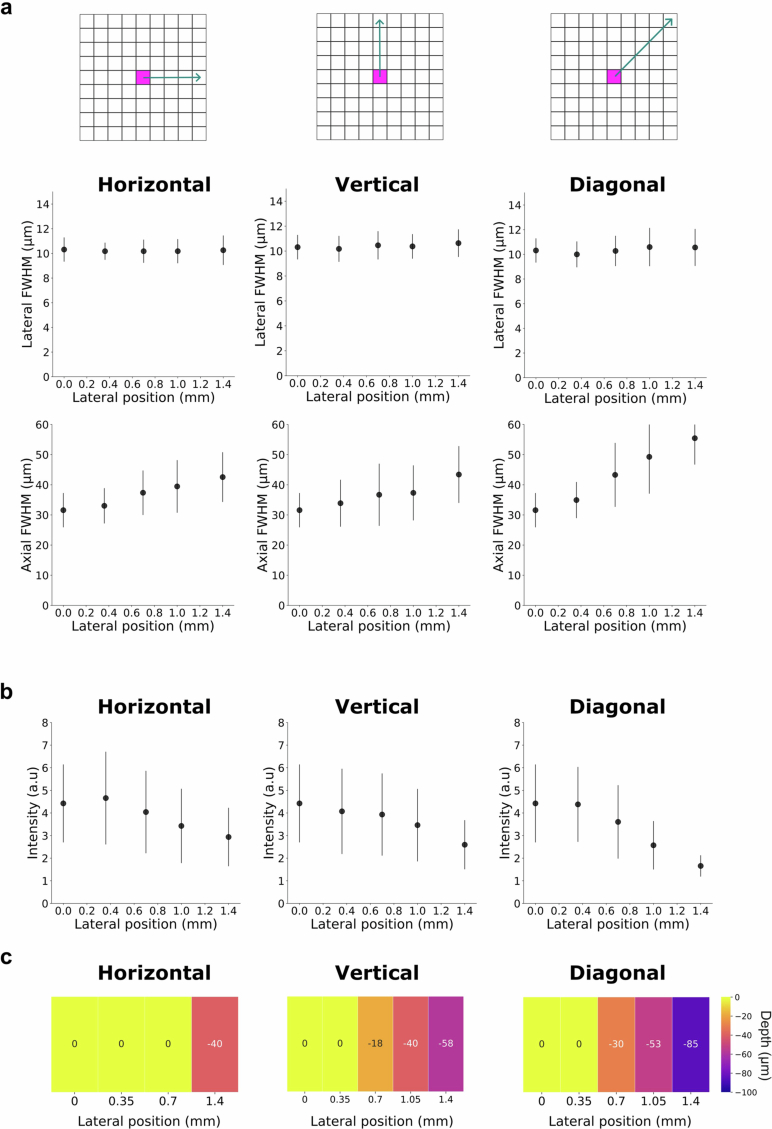
Optical characterization of random-access 3D-SHOT. **a**) Average lateral and axial resolution measured across 3D distribution of spots at different galvanometer scan positions across the horizontal, vertical and diagonal axes. Error bars indicate standard deviation values. Middle row (lateral resolution) horizontal: n = 1156, 243, 276, 246, 225 holograms respectively for 0mm, 0.35mm, 0.7mm, 1.05mm and 1.4mm galvo positions; vertical: n = 1156, 511, 505, and 636 holograms respectively for 0mm, 0.35mm, 0.7mm, 1.05mm and 1.4mm galvo positions; diagonal: n = 1156, 355, 355, 221 and 318 holograms respectively for 0–0mm, 0.35–0.35mm, 0.7–0.7mm, 1.05–1.05mm and 1.4–1.4mm galvo positions. Bottom row (axial resolution): horizontal: n = 380, 176, 169, 142 and 114 holograms respectively for 0mm, 0.35mm, 0.7mm, 1.05mm and 1.4mm galvo positions; vertical: n = 380, 175, 182, 179 and 164 holograms respectively for 0mm, 0.35mm, 0.7mm, 1.05mm and 1.4mm galvo positions; diagonal: n = 380, 172, 161, 130 and 74 holograms respectively for 0–0mm, 0.35–0.35mm, 0.7–0.7mm, 1.05–1.05mm and 1.4–1.4mm galvo positions. **b**) Average single-hologram intensity measured across 3D distribution of spots at different galvanometer scan positions across the horizontal, vertical and diagonal axes. Error bars indicate standard deviation values. Horizontal: n = 380, 176, 169, 142 and 114 holograms respectively for 0mm, 0.35mm, 0.7mm, 1.05mm and 1.4mm galvo positions. Vertical: n = 380, 175, 182, 179 and 164 holograms respectively for 0mm, 0.35mm, 0.7mm, 1.05mm and 1.4mm galvo positions. Diagonal: n = 380, 172, 161, 130 and 74 holograms respectively for 0–0mm, 0.35–0.35mm, 0.7–0.7mm, 1.05–1.05mm and 1.4–1.4mm galvo positions. **c**) Axial offset between imaging plane and holographic plane z=0 plane (no defocus). Source data

To maintain high diffraction efficiency across the FOV, we tiled the mesoscopic FOV into 81 quadrants of 350 µm × 350 µm, each centered on a unique (X, Y) galvanometer position. This strategy enabled consistent patterned photostimulation across a 3.2 mm span (Fig. 4c), representing nearly a tenfold expansion of the accessible photostimulation field. We validated that the random-access holographic configuration retained the axial resolution performance of the mesoscale 3D-SHOT system, as presented above, at the center of the FOV (mean axial full-width-half-maximum (FWHM) = 34 µm, *n* = 154 holograms; Fig. 4d). We also validated that the relative distances between holograms are maintained with galvo scanning (Supplementary Fig. 8). We characterized the axial resolution and the hologram intensity as the galvo is scanning across the mesoscopic FOV (Fig. 4e,f and Extended Data Fig. 7). Hologram intensity decreased by only ~2.5-fold from the center to the corner of the 3.2 mm × 3.2 mm FOV, requiring only a 1.5-fold adjustment in photostimulation power to compensate. Hologram optical axial resolution decreased across the mesoscopic FOV, remaining below 50 µm over more than two-thirds of the area, with a worst-case edge average axial resolution of 56 µm. We characterized the axial field curvature across the mesoscopic FOV and found a maximum relative shift of 85 µm between the flattened imaging plane and the holographic planes, a variation that remains well within the SLM’s defocusing correction range (Extended Data Fig. 7c). Finally, as described above, we characterized the functional PSF (that is, PPSF) at an off-center galvanometer position. Although near-single-cell resolution was achieved at the lowest photostimulation powers, effective functional axial resolution degraded more rapidly at higher powers (~82 µm FWHM at the highest power probes beyond saturating power; Supplementary Fig. 9). The measured values remained at the upper end, but within the range of reported PPSF values for patterned 2p optogenetics. The optical lateral resolution of the holograms was independent of the galvo position (Extended Data Fig. 7). We further examined the neural activity of non-target cells as a function of their distance to the nearest target and observed that the lateral holographic FOV did not exhibit an increase in off-target responses in comparison to the central FOV (Supplementary Fig. 10). We next demonstrated that random-access 3D-SHOT could reliably activate holograms from arbitrary locations within the 3.2 × 3.2 mm^2^ mesoscopic photostimulation FOV (Fig. 4g,h). By dynamically updating the holographic phase mask for each selected galvanometer (X, Y) position, we successfully targeted and stimulated user-defined groups of neurons across distinct cortical areas within the same experiment, in a randomized trial-by-trial manner.

### Causal mapping of functional connectivity across cortical areas

**Fig. 5:**
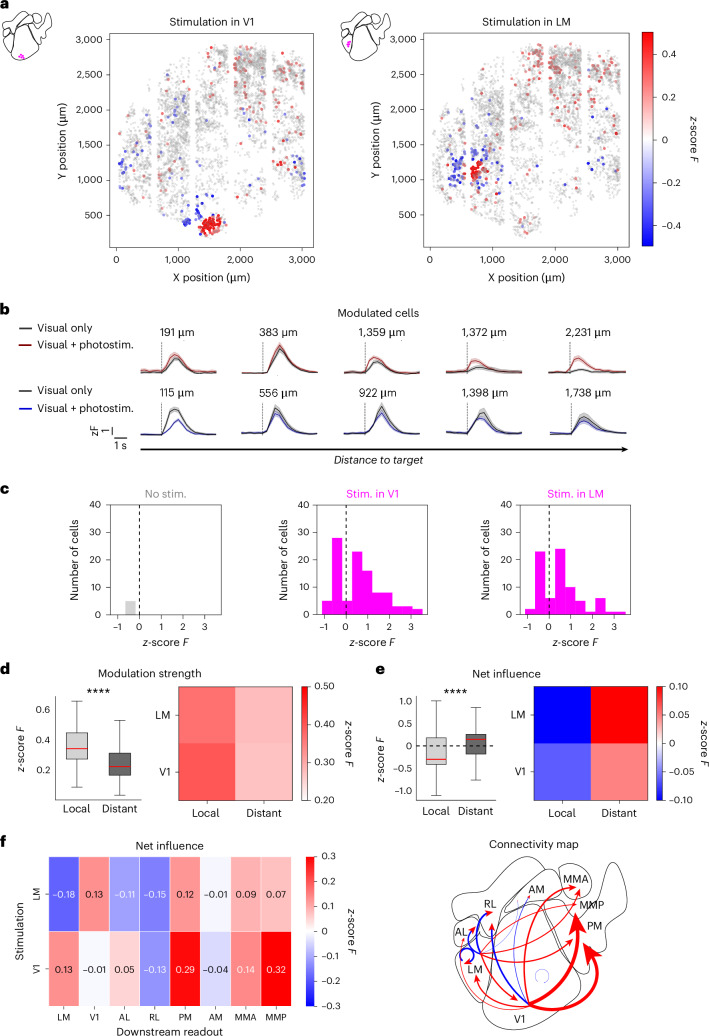
Mesoscale causal probing of connectivity across cortical areas. **a**, Example mesoscale holographic ‘influence’ maps measured in response to activation of an ensemble of V1 neurons (left) and an ensemble of LM neurons (right). Significantly modulated cells (including targets) are colored according to the amplitude difference between their calcium responses in photostimulation trials and their calcium responses in control (no photostimulation) trials. Non-modulated cells are displayed in gray. Experiment performed on a mesoscopic 3.1 mm × 3.1 mm FOV recorded at 3 Hz, in a *CaMK2*-tTA; tetO-GCaMP6s transgenic mouse injected locally with viral pAAV-hSyn-GCaMP6m-p2A-ChRmine-Kv2.1-WPRE at discrete locations. Photostimulation with ten pulses (10 ms, 30 Hz, 9 mW). **b**, Example average calcium responses of individual modulated cells. Red and blue represent average responses on photostimulation trials (*n* = 100 trials). In this example, photostimulation is combined with visual stimulation (Gaussian-modulated noise; see Methods). Black represents an average response on control trials (visual stimulation with no holographic photostimulation, *n* = 100 trials). Error bands, 95% confidence intervals around the mean, computed by bootstrapping. **c**, Distribution of post-stimulation modulation in targets (*n* = 10) and follower cells. Post-stimulation modulation is defined as the difference between responses in photostimulation and no-power control trials. Follower cells are defined as reliably modulated cells across non-overlapping trial sets (see Methods). **d**, Modulation strength of the holographic perturbation on the non-targeted network. Modulation strength is defined as the average absolute value of the mean response across all follower cells, regardless of response polarity. Both excitatory and inhibitory modulations are therefore counted as positive values (see Methods). For LM photostimulation, the local network corresponds to all follower cells within LM and farther than 100 µm from the targets. The distant network corresponds to all follower cells outside of LM. For V1 photostimulation, all target holograms were located in posterior V1. The local network corresponds to all follower cells within 100 µm to 1 mm from the targets. The distant network corresponds to all follower cells outside V1. Local modulation strength: 0.39 ± 0.01 (mean ± s.e.m.), *n* = 287 cells, six sessions, two mice. Distant modulation strength: 0.28 ± 0.01 (mean ± s.e.m.), *n* = 607 cells, six sessions, two mice. Two-sided Mann–Whitney *P* = 7.14 × 10^−28^. Stimulated ensemble size varied between ten and 60 cells stimulated per hologram per session. For each session, the hologram size was matched between LM and V1 stimulations. Box-and-whisker plots are formatted as follows: red centerline, median; box limits, upper and lower quartiles; whiskers, 1.5× interquartile range. **e**, Net influence of the holographic perturbation on the non-targeted network. Net influence is defined as the average signed value of the mean response across all follower cells (see Methods). Local net influence: −0.19 ± 0.02 (mean ± s.e.m.), *n* = 287 cells, six sessions, two mice. Distant net influence: 0.1 ± 0.01 (mean ± s.e.m.), *n* = 607 cells, six sessions, two mice. Two-sided Mann–Whitney *P* = 4.97 × 10^−26^. Box-and-whisker plots are formatted as follows: red centerline, median; box limits, upper and lower quartiles; whiskers, 1.5× interquartile range. **f**, Inter-areal causal mapping of net influence across cortical areas computed from post-stimulation responses of follower cells. *n* = 6 sessions, two mice, including *n* = 4 sessions, two mice with no visual stimulation and *n* = 2 sessions, two mice with concurrent visual stimulation. Area boundaries are defined using online 2p retinotopic mapping (see Supplementary Fig. 11 and Methods). Left, connectivity matrix of net influence. Right, inter-areal connectivity map. Arrow size is proportional to net influence. Blue or red indicates either inhibitory or excitatory influence, respectively. See functional connectivity statistics in Supplementary Table 3 and Supplementary Note 4. Source data

**Extended Data Fig. 8:**
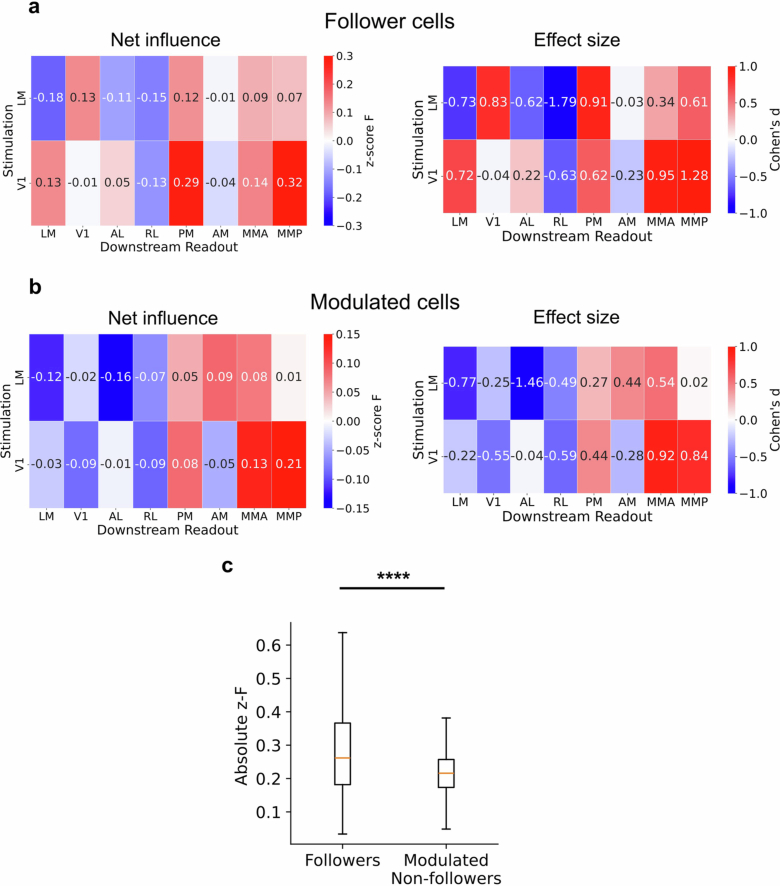
Inter-areal functional connectivity maps from follower cells and modulated cells. **a**) Left: Inter-areal causal mapping of net influence across cortical areas computed from post-stimulation responses of follower cells (a subset of highly reliable modulated cells, see Methods). N=6 sessions, 2mice. Area boundaries are defined using online two-photon retinotopic mapping (see Supplementary Fig. 11 and Methods). This connectivity matrix hence corresponds to a functional connectivity matrix of strong and sparse connections. Right: Statistical effect size across n=6 sessions. The effect size is measured here by the Cohen’s d metric, defined in this case as $\frac{{mean}}{{std}}$ across all sessions. A cohen’s d of 0.2 represents a small effect, 0.5 a medium effect and 0.8 and above a large effect. **b**) Left: Same as a) but computed from post-stimulated responses of modulated cells (all cells significantly responsive to photostimulation, see Methods). This connectivity matrix hence by definition also includes weaker connections. Right: Statistical effect size for the net influence map computed from modulated cells. **c**) Comparison of absolute response amplitude of follower cells and modulated non-follower cells. Followers: mean 0.31 (n = 890 cells), minimum 0.034, maximum 2.81, median center 0.26, box lower Q1 25^th^ 0.18, box upper Q3 75^th^ 0.37, lower whisker 0.034, upper whisker 0.64, 95^th^ percentile 0.61.Non-followers: mean 0.22 (n = 3460 cells), minimum 0.045, maximum 0.79, median center 0.22, box lower Q1 25^th^ 0.17, box upper Q3 75^th^ 0.26, lower whisker 0.049, upper whisker 0.38, 95^th^ percentile 0.34. Boxes boundaries indicate lower quartile Q1(25^th^ percentile) and upper quartile (75^th^ percentile), orange bar indicates median. Whiskers indicated 1.5 x interquartile range. One-sided Mann-Whitney U test, p-value: 2.1 × 10^−36^. Source data

**Extended Data Fig. 9:**
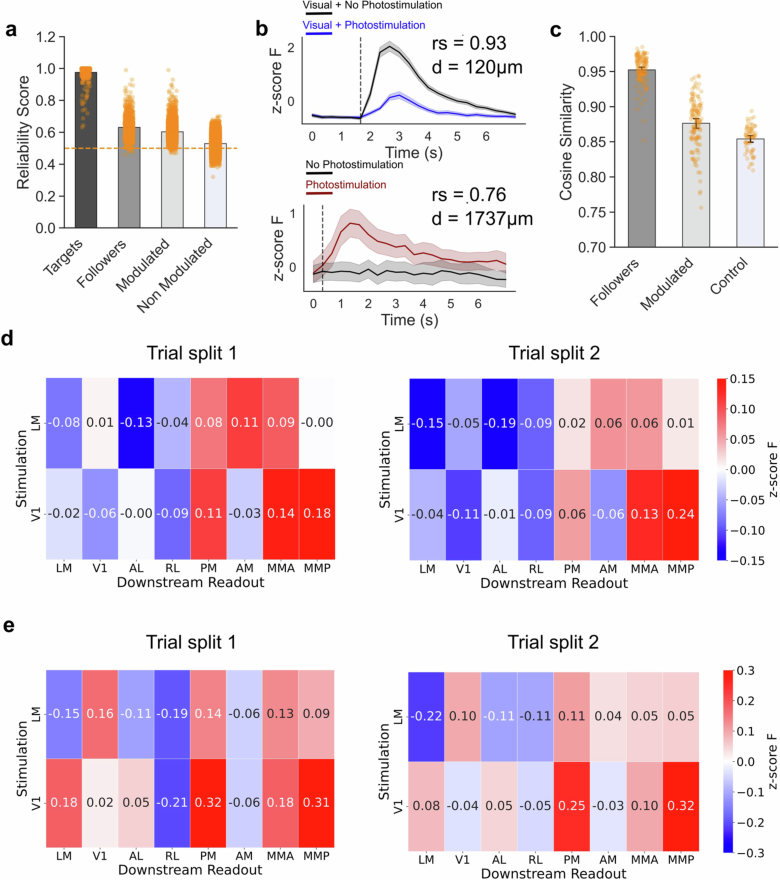
Reliability of post-synaptic responses across trials. **a**) Single-trial reliability of target, follower, modulated, and non-modulated cells. Modulated cells were located >100 µm from the nearest target and showed significant responses to photostimulation (p < 0.05; see Methods). Follower cells were defined as a subset of highly reliable modulated cells (see Methods). Reliability was quantified as the fraction of trials in which the sign of post-stimulus modulation matched that of the average modulation. Post-stimulus modulation was computed as the difference between responses in photostimulation and control (no-power) trials. *n* = 417 (targets), 931 (followers), 4,298 (modulated), and 50,629 (non-modulated), pooled across 6 sessions from 2 animals. Mean reliability scores: 0.98 (targets), 0.63 (followers), 0.6 (modulated), 0.53 non-modulated. Error bars indicate bootstrapped 95% confidence interval. Dashed line indicates chance level. To avoid sample size bias, 1,000 cells were randomly subsampled from each non-target group for statistical testing. Two-sided Mann-Whitney U tests: Followers vs. Non-modulated, p = 3.14x10^−140^; Followers vs. Non-modulated, p= 3.1×10^−225^; Modulated vs. Non-modulated, p = 6.2×10^−218^. **b**) Example calcium responses from follower cells with high reliability. Average calcium traces from two follower cells with reliability scores of 0.93 and 0.76. Blue or red traces show responses during photostimulation trials; black traces indicate responses during control (no-power) trials. The shaded area represents the bootstrapped 95% confidence interval. **c**) Similarity of population response vectors across bootstrapped trial splits. For each experiment, photostimulation trials were randomly divided into two non-overlapping subsets, repeated over 10 bootstrap iterations. Population vectors of post-stimulus modulation were computed separately for each subset and condition: follower cells, modulated cells, and a control group consisting of significantly modulated cells in no-power trials. Both modulated and follower cells showed significantly higher cosine similarity than control cells (Two-sided Mann–Whitney U test: modulated vs. control, *p* = 8.4×10^−7^; follower vs. control, *p* = 3.5E-27). *n* = 120 (modulated), 120 (follower), and 60 (control). Mean similarity: 0.95 (follower), 0.88 (modulated), and 0.85 (control). The distribution of follower cell cosine similarity was entirely non-overlapping with control values. Error bars indicate bootstrapped 95% confidence interval. **d**) Inter-areal functional connectivity maps from trial-split population responses. Connectivity maps were computed from modulated cells using population vectors derived from two non-overlapping subsets of both photostimulation trials and control (no-power) trials. N=2 animals, 6 sessions. **e**) Inter-areal functional connectivity maps from trial-split population responses. Connectivity maps were computed from follower cells using population vectors derived from two non-overlapping subsets of both photostimulation trials and control (no-power) trials. N=2 animals, 6 sessions. Source data

One of the major advantages of mesoscale 2p holographic optogenetics could be the ability to map functional interactions between multiple areas simultaneously with near-cellular resolution. To test this possibility, we stimulated ensembles of neurons in either primary visual area V1 or visual area LM, while recording from neurons across more than seven other cortical areas (Fig. 5a and Supplementary Fig. 11) in a total 3.1 mm × 3.1 mm FOV recorded at 3 Hz. We detected downstream neurons both activated and suppressed upon photostimulation (Fig. 5b), located both within the same visual area as the targets and at distances of hundreds of microns to millimeters away. For connectivity analyses, we defined follower cells as highly reliable connections, whereas modulated cells encompassed all significantly responsive cells to photostimulation (see Methods). We additionally found that follower cells exhibited stronger response amplitudes than non-follower modulated cells (Extended Data Fig. 8), which indicates that follower cells represent strong functional connections while modulated cells encompass both strong and weaker connections. As a control, we compared the distribution of follower cells in photostimulation trials to that of statistical ‘follower cells’ identified in no-power (0 mW) control trials (Fig. 5c), confirming that holographic stimulation induces postsynaptic modulation above the false positive rate (Fig. 5c and Supplementary Figs. 12 and 13). Importantly, we found that these downstream modulations were consistent across trial splits, suggesting a deterministic impact of the photostimulation on the network despite the variability of ongoing spontaneous activity (Extended Data Fig. 9).

**Extended Data Fig. 10:**
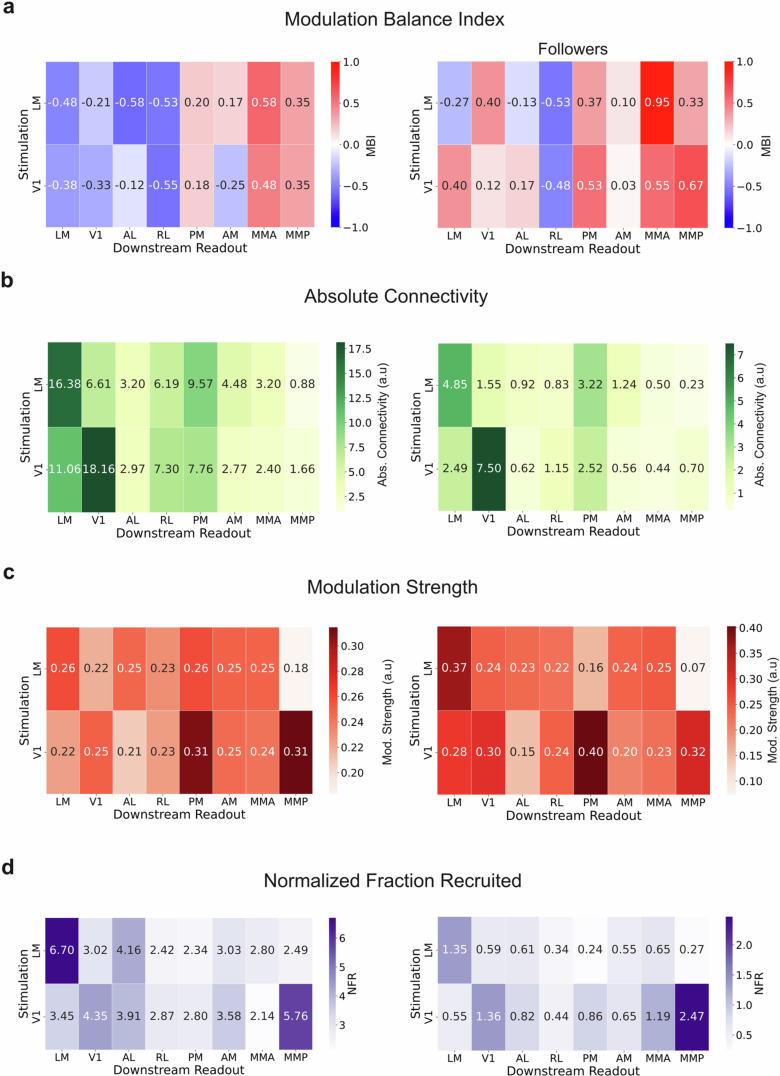
Inter-areal functional connectivity metrics for photostimulation in V1 and LM. The modulation balance index quantifies the relative dominance of excitation vs. inhibition among significantly modulated downstream neurons (left) or follower neurons (right). It is defined as Modulation Balance Index = $\frac{{N}_{{pos}}-{N}_{{neg}}}{{N}_{{pos}}+{N}_{{neg}}}$ where N_pos_ and N_neg_ are the numbers of significantly positively and negatively modulated cells, respectively. The index ranges from -1 (pure inhibition) to +1(pure excitation), with 0 indicating a balanced mix. **b**) The absolute connectivity metric quantifies the total strength of downstream modulation elicited by photostimulation, regardless of polarity. It is calculated as the sum of the absolute modulation responses across all significantly modulated neurons (left) or follower neurons (left). Absolute Connectivity = ${\sum }_{i=1}^{n}\left|{R}_{i}\right|$ where the sum is taken over all n significantly modulated cells. This metric reflects the overall magnitude of the network response. **c**) The modulation strength metric quantifies the average amplitude of photostimulation-induced responses among significantly modulated neurons (left) or follower neurons (right). It is computed as the sum of the absolute modulation amplitudes divided by the number of significantly modulated cells N_mod_. Modulation Strength = $\frac{{\sum }_{i=1}^{n}\left|{R}_{i}\right|}{{N}_{\mathrm{mod}}}$. This provides a polarity-independent measure of the average effect size per modulated neuron, allowing comparison of response strength across conditions or cortical areas regardless of the number of recruited cells. All metrics were computed from significantly modulated cells within each visual area (n = 6 sessions, 2 mice). d) The Normalized Fraction Recruited metric is computed as the number of recruited cells (followers or modulated) divided by the number of stimulated cells and separately divided by the number of detected cells in each area. See functional connectivity statistics in Supplementary Table 3 and Supplementary Note 3.

We first quantified the strength of holographic perturbations on both local and distant networks (Fig. 5d). We defined the local network as cells located in the photostimulation region 100–1,000 µm from the nearest target, and distant neurons as all neurons in other areas. We computed the modulation strength as the sum of the absolute modulation amplitudes of follower cells divided by the number of follower cells (see Methods) to quantify the average amplitude of photostimulation-induced responses independently of polarity. We found that holographic perturbations produced significantly stronger modulation within the targeted cortical area compared to distant areas (Mann–Whitney *P* = 7.14 × 10^−28^), consistent with the high density of local recurrent connections in visual cortex^25,26^. Next, we examined the net modulation impact of holographic photostimulation in V1 and LM. In both areas, we observed a significant inhibitory effect on the local network (Fig. 5e), confirming and extending previous findings^3,5,27,28^. Notably, in downstream areas, the net effect in follower cells was excitatory (Fig. 5e). To more clearly reveal these long-range modulatory effects, we constructed functional connectivity maps based on the responses of follower cells to holographic perturbations per area, both for stimulation in V1 and in LM (Fig. 5f). Although an exhaustive mapping of functional connectivity in the visual cortex is beyond the scope of this paper and no mechanistic claim on functional connectivity can be made from this demonstration dataset, these initial perturbation experiments suggest that our platform can detect structured connectivity patterns across cortical areas above noise (Extended Data Figs. 9 and 10, Supplementary Note 4 and Supplementary Table 3).

### Rapidly interleaved photostimulation of remote neural ensembles in different cortical areas

**Fig. 6:**
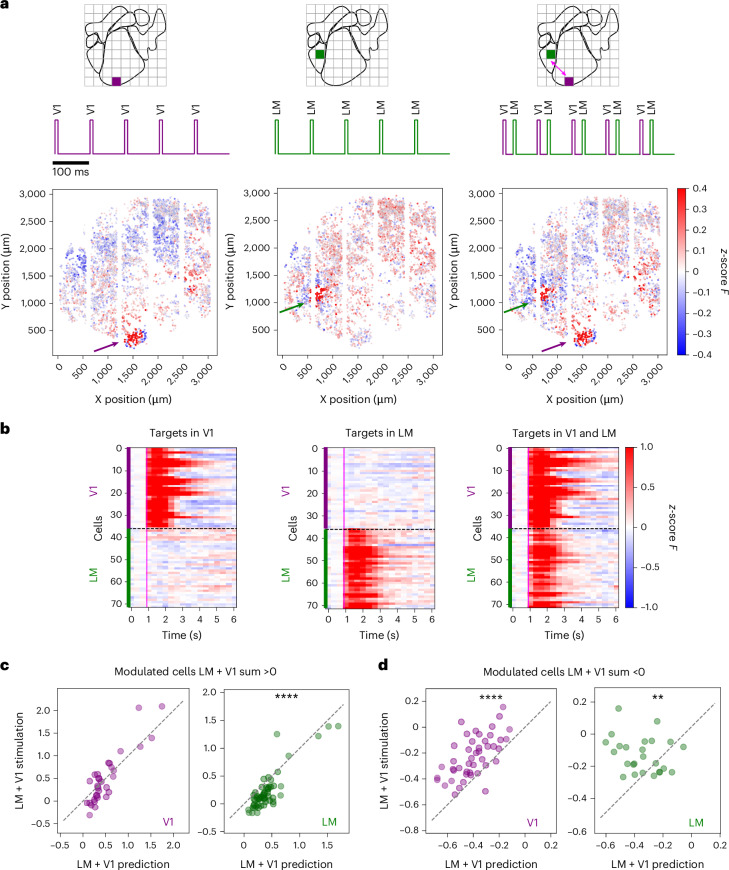
Rapidly interleaved photostimulation of distant neural ensembles. **a**, Top, principle of rapidly interleaved photostimulation on the mesoscope with hologram interleaving: 10 Hz pulsed stimulation in V1 with hologram 1 (left), 10 Hz pulsed stimulation in LM with hologram 2 (center) and fast interleaved 10 Hz hologram 1 and hologram 2 stimulation (right) at 52.6 Hz galvanometer interleaving rate (that is, 19 ms between end of stimulation pulse for hologram 1 and start of stimulation pulse for hologram 2). Bottom, mesoscopic influence maps for single-hologram perturbation in V1 (left), single-hologram perturbation in LM (center) and joint interleaved holograms in V1 and LM (right). All cells detected in the FOV are displayed with a color displaying their relative response to photostimulation. Arrows indicate holographic perturbations. In total, 35 cells were photostimulated in LM and 35 cells were photostimulated in V1. **b**, Average post-stimulus calcium responses when stimulating one single neural ensemble in V1 (left), one single neural ensemble in LM (center) and both co-activated neural ensembles (right). Experiment performed on a mesoscopic 3.1 mm × 3.1 mm FOV recorded at 3 Hz, in a *CaMK2*-tTA; tetO-GCaMP6s transgenic mouse injected locally with viral pAAV-hSyn-GCaMP6m-p2A-ChRmine-Kv2.1-WPRE at discrete locations. Photostimulation with ten pulses (10 ms,10 Hz, 12.5 mW). **c**,**d**, Scatter plots showing for every modulated cell by V1 perturbation (purple) or LM perturbation (green), the calcium response to the joint V1 + LM holographic photostimulation against the predicted calcium response computed from summing responses to single V1 stimulation and LM stimulation. Given the non-normality of the distribution, responses are split depending on whether the predicted sum V1 + LM is positive (**c**) or negative (**d**). *n* = 2 mice, three sessions. In **c**, the left panel shows *n* = 32 modulated cells from V1 stimulation, fraction of sublinear cells 0.59, *P* = 0.50 (two-sided Wilcoxon signed-rank test); the right panel shows *n* = 56 modulated cells from LM stimulation, fraction of sublinear cells 0.91, *P* = 4.49 × 10^−9^ (two-sided Wilcoxon signed-rank test). In **d**, the left panel shows *n* = 45 modulated cells from V1 stimulation, fraction of sublinear cells 0.044, *P* = 3.74 × 10^−8^ (two-sided Wilcoxon signed-rank test); the right panel shows *n* = 25 modulated cells from LM stimulation, fraction of sublinear cells 0.28, *P* = 0.001815 (two-sided Wilcoxon signed-rank test). Source data

Finally, we took advantage of the high speed of the galvanometer scanners and the rapid refresh rate of the SLM to synchronize the phase mask to the galvo position for every optogenetic stimulation pulse, enabling the interleaving of two remote holograms (Fig. 6a). With a 10 × 10 ms pulse stimulation at 10 Hz, the two remote neural ensembles would be coactive within a 950 ms time window. Using this strategy, we stimulated a single neural ensemble in V1, a separate single ensemble in LM and then co-activated both ensembles (V1 + LM) with rapidly interleaved holograms. The resulting average post-stimulus histograms showed that in the latter case, both ensembles were effectively activated simultaneously (Fig. 6b). Lastly, we leveraged this experiment to demonstrate how this fast scan capability can enable new experimental paradigms for investigating inter-areal computations. In particular, it is unknown whether the functional impacts of two long-range projection pathways that converge on a single downstream area will summate linearly or non-linearly. In our demonstration dataset, we compared the net impact of photo-stimulating ensembles in V1 and LM simultaneously to the predicted sum of the effects when each was stimulated in isolation (Fig. 6c,d). We found that, anecdotally, the net effect of remote ensemble coactivation yielded modulations on individual downstream neurons that were less than the predicted sum, potentially indicative of normalization mechanisms^29^. In V1-modulated cells, we also identified a small number of highly modulated ‘amplifier’ cells that showed the opposite trend and exhibited supra-linear summation. Of note, these observations are mostly intended to illustrate the types of mechanistic hypotheses that can be probed with this approach rather than establish definitive biological conclusions. Overall, these findings illustrate how the holographic 2p mesoscope platform enables new classes of experiments that have until now been completely inaccessible with existing technologies.

## Discussion

We designed, built and validated what is, to our knowledge, the first multiphoton holographic mesoscope. This technology offers targeted photostimulation approximating single-cell resolution, with simultaneous large-scale recording of neural activity across several cortical areas in a contiguous mesoscopic FOV. We demonstrated that engineering a scanning module within the photostimulation system increases the accessible photostimulation FOV by an order of magnitude and enables rapidly interleaved photostimulation of spatially remote neural ensembles. We established the new system’s ability to co-activate user-defined groups of neurons, selected based on their spatial or functional properties, while simultaneously recording neural activity from several downstream areas. Notably, we revealed that specific functionally relevant information can be holographically written in and propagated to downstream networks. We could identify individual downstream neurons that displayed time-locked excitatory or inhibitory responses hundreds or even thousands of microns away. We showed that this approach provides a foundation for constructing inter-areal cortical connectivity maps based on targeted causal perturbations. Overall, this demonstrates that one can use this new technology to probe how both local and long-range functional interactions relate to specific neuronal computations, all within the same experiment.

We developed the mesoscale read–write platform based on a commercially available 2p mesoscope (Thorlabs), a widely used mesoscope platform^30–34^ that has enabled a number of other recent technological advances^35–37^. We expect that adapting a commercially available system will facilitate the adoption of the technology across both individual labs and new neurotechnology platforms. In SLM-based holographic systems, the size of the holographic FOV is fundamentally limited by the pixel count and pixel size of the SLM. Ongoing advances in high-resolution SLM technology—including increased pixel density and reduced pixel size—may eventually enable large-FOV photostimulation using a single SLM. However, such approaches will continue to be limited by reduced power efficiency at high diffraction angles and potential inter-pixel crosstalk, both of which can constrain the effective usable FOV. Our random-access 3D-SHOT strategy is inherently compatible with and can further amplify the benefits of these future SLM developments, enabling even larger and more flexible photostimulation fields. The concept of expanding the SLM FOV using galvanometer scanning has been previously explored for holographic imaging^38^ and proposed conceptually for photostimulation^39^. However, to our knowledge, this is the first implementation of such an approach at the mesoscale, integrated within a constrained multi-4*f* holography and temporal focusing layout (3D-SHOT). Prior studies have demonstrated random-access holographic imaging over 0.15 mm^2^ (ref ^38^) and structured illumination across 1.64 mm^2^. By contrast, our platform enables 2p photostimulation across more than 10 mm^2^, while simultaneously recording from thousands of neurons distributed across multiple cortical areas.

Recent studies have initiated the use of 2p optogenetics to probe inter-areal communication, but they remain constrained to interactions between immediately adjacent cortical regions^20,27^. One recent study investigated inter-areal communication using 2p optogenetics on a conventional 2p microscope by imaging across the boundary of two contiguous cortical regions^27^, and another, more recent study also enabled inter-areal interrogation between two contiguous areas, using a low NA ×10 objective to achieve a 2 mm × 2 mm imaging FOV^20^. In terms of imaging capability, our platform enables recording of 18-fold more cortical surface than the first study^27^, and significantly higher axial resolution with four times more cortical surface than the second study^20^. Importantly, the full 5 mm × 5 mm nominal FOV of our platform enables random-access selection of imaging regions of interest (ROIs) anywhere within the 25 mm^2^ FOV. In terms of photostimulation capability, our secondary large-FOV design expands the photostimulation FOV by tenfold compared to any other published work. A limitation of the current design is the trade-off between resolution and large-FOV access. However, the observed resolution degradation is primarily caused by off-axis scan aberrations and could be substantially mitigated with a custom large-aperture scan lens, offering a potential route for improving performance in future implementations.

Our large-scale holographic perturbation experiments provide a demonstration that mesoscale 2p optogenetics can be used to map functional connectivity across multiple cortical areas with near-cellular resolution. By selectively stimulating neural ensembles in V1 and LM while recording activity across the dorsal visual cortex, we revealed distinct patterns of excitatory and inhibitory influence. Notably, local networks showed net suppression, whereas downstream areas often exhibited net excitation, suggesting divergent circuit motifs within and across areas. Importantly, these results are presented for demonstration purposes, and more extensive functional connectivity studies with substantially larger and more highly powered datasets will be required to establish mechanistic and biological interpretations. Nonetheless, these findings highlight mesoscale holographic optogenetics as a powerful approach for dissecting inter-areal functional influences with high spatial precision, enabling systematic investigation of long-range cortical interactions.

Although there is substantial work detailing how individual cortical areas represent and encode information, how neural representations are transformed across cortical areas remains elusive. Most studies addressing cortico–cortical communication combined multi-site electrode recordings with correlative analyses^40–42^, or electrical or one-photon optogenetic stimulation. However, electrical microstimulation provides limited spatial control and often (and perhaps preferentially) activates axons of passage^43^. One-photon optogenetic stimulation has cell-type-specific control but can not readily activate functionally defined subsets of neurons (such as neurons with the same feature preference). Molecular approaches such as c-Fos and TRAP^44–46^, when combined with one-photon optogenetics, offer some degree of functional specificity but within a far less flexible framework than 2p optogenetics, as they do not permit the selection of cells based on multiple functional features. Neither microstimulation nor one-photon optogenetic stimulation can recreate precise spatiotemporal sequences of activity, nor can they drive different user-defined firing rates into different neurons to recreate specific population activity vectors that might preferentially drive cortico–cortical communication^41^; 2p holographic optogenetics can achieve these goals^17,19^, and this new 2p holographic mesoscope can implement these perturbations while simultaneously measuring both local and downstream neural activity. Thus, 2p mesoscale holography is set up to experimentally test major hypotheses and theories of inter-areal communication, such as how cortico–cortical feedback implements generative models of the external world, such as in the framework of predictive coding^47–50^.

The 2p holographic mesoscope could also guide the design of more effective brain–machine interfaces^51^. Access to intermediate processing stages between targeted neural perturbations and behavioral output will be essential for interpreting cases in which stimulation fails to produce the expected outcome. Understanding and optimizing signal propagation across areas may represent a critical strategy for enhancing the efficacy of holographic interventions in driving behavior^6,8,27,52^.

Finally, the large accessible FOV of the mesoscope will help scale 2p optogenetics to species with larger brains, such as primates. We expect mesoscale 2p holographic optogenetics to become a key technology in systems neuroscience, as it can establish whole new classes of perturbative experiments to motivate and test previously untestable theories of brain function.

## Methods

### Animals

All experiments on animals were conducted with approval of the Animal Care and Use Committee of the University of California, Berkeley. All experiments were performed in mice of both sexes, aged 2 months and older. Three transgenic mouse lines were used: either Ai203 transgenic lines^19^ crossed in-house with *Vglut1*-Cre mice, or double (*CaMK2*-tTA; tetO-GCaMP6s) or triple (*emx1*-Cre; *CaMK2*-tTA; tetO-GCaMP6s) mice, although no Cre-dependent viruses were used in this study, except in Supplementary Fig. 13. Transgenic mice were obtained by crossing the corresponding lines in-house (JAX stock nos. 005628, 003010 and 024742). Mice were housed in groups of five or fewer in a reverse light–dark cycle of 12:12 h. Experiments were conducted during the dark phase.

### Surgery

Mice were anesthetized with isoflurane (2%) and given buprenorphine as an analgesic (0.05 mg kg^−1^) and dexamethasone (2 mg kg^−1^) to reduce brain swelling. Mice were then placed in a stereotaxic frame (Kopf) over a heating pad. The scalp was removed, the fascia pushed to the sides and the skull lightly scratched for better cement adhesion. A 4 mm or 5 mm craniotomy centered around V1 was made using a biopsy punch (Robbins Instruments) and/or a dental drill (Foredom). Bleeding was controlled with cold PBS and Gelfoam (Pfizer). For mice that required viral injection (double and triple transgenics), the virus preparation (pAAV-hSyn-GCaMP6m-p2A-ChRmine-Kv2.1-WPRE or AAV9-CAG.DIO.ChroME2s-FLAG-ST.P2A.H2B.mRuby3.WPRE.SV40) was injected using a beveled glass pipette at four to five locations across the visual cortex. A cranial window, made of 7 mm and 5 mm diameter glass coverslips or 6 mm and 4 mm diameter glass coverslips for 5 mm and 4 mm craniotomies, respectively, was then placed onto the craniotomy and held in place with Metabond (C&B). Finally, a titanium headplate was fixed to the skull with Metabond (C&B0 and Ortho Jet (Lang). All craniotomies were performed on the left hemisphere of the mouse brain. Animals were allowed to recover in a heated recovery cage before being returned to their home cage.

### 2p-RAM mesoscope with a temporally focused holographic path

The mesoscale read–write platform was custom-built around a 2p random-access fluorescence mesoscope previously described in detail^10^ and now commercialized by Thorlabs. The system had a nominal 5 mm FOV accessed through a 0.6 NA objective that can be rapidly and flexibly scanned across with a set of four conjugated scanners, one 12 kHz resonant scanner and three large-angle galvanometers. The system was also equipped with a voice-coil remote-focusing module^54^ for 3D multi-plane imaging. In addition, the mesoscope was built on a vertical breadboard, and the whole system was motorized in four dimensions (X, Y, Z translation and one axis of rotation) to provide maximal flexibility for the user. Given that the microscope moved with respect to the incoming laser beam, the beam was injected through a six-stage periscope for alignment invariance.

The 2p mesoscale imaging path relied on a titanium–sapphire laser (Mai Tai, Spectra Physics) used at 920 nm for calcium imaging. External power control was achieved through a Pockels cell (Conoptics). A prism-based group delay dispersion compensation module^55^ was installed to compensate for dispersion introduced by the mesoscope imaging path optics (estimated at 25,000 fs^2^)^10^. The 2p holographic path relied on a 1,030 nm femtosecond fiber laser (Aeropulse 50, NKT Photonics) with internal power control.

The holographic path was built on a 33 inch × 24 inch breadboard attached to the main mesoscope frame with three custom mounts (see Supplementary CAD). The holographic beam was merged with the imaging beam with a 1,000 nm cutoff dichroic (DMSP1000R, Thorlabs) and rigorously co-aligned with the imaging beam through the system’s periscope to maintain alignment invariance with translation. The beam was then injected onto the 3D-SHOT optical path (see Supplementary Table 1 and Supplementary CAD) with specific care to maintain the beam horizontality to achieve alignment invariance with rotation (see Supplementary Note 1).

To expand the holographic FOV while preserving hologram resolution at the center (Fig. 4), a compact 1:2 Galilean telescope was inserted into a collimated section of the optical path after the SLM zero-order (between lenses L5 and L6). Large-aperture 30 mm XY galvanometer scanners (QS30XY-AG, Thorlabs) were added to maintain optical conjugation with both the SLM plane and the objective’s back aperture. The original L6 lens was replaced with a 100 mm *f*-theta scan lens (FTH100-1064, Thorlabs) to restore axial hologram resolution, and the recombination dichroic (D3) was replaced with a larger version to accommodate the large beam scanning. Finally, the remote-focusing voice coil used for imaging was adjusted to achieve coplanarity between the imaging and holographic planes at the center of the FOV.

The imaging path hardware was controlled with ScanImage software S2018B (Vidrio Technologies). Custom MATLAB code was used for control of the photostimulation path hardware, synchronization with imaging, synchronization of galvo scanners and SLM phase masks, and control of the visual stimulation.

### SLM stimulation FOV

For SLM nominal FOV calculation, the maximal lateral deflection from the SLM is achieved for the smallest effective ‘slit’ size on the SLM, which would correspond to two pixels (the pattern on the SLM would then be a black and white grating pattern with a pixel-sized stripe pattern). Based on the grating equation and taking into account that the effective pixel size at the objective back aperture is magnified by the microscope optics, the nominal maximal FOV is given by:

FOV = 2× $\frac{\lambda \times {f}_{\mathrm{obj}}}{2\times {p}_{\mathrm{BA}}}$, where λ is the laser wavelength, ${f}_{\mathrm{obj}}$ is the objective focal length and ${p}_{\mathrm{BA}}$ is the effective SLM pixel size at the back aperture (${p}_{\mathrm{BA}}=p\times \frac{f{\rm{L}}5}{f{\rm{L}}4}\times \frac{f\mathrm{TL}}{f{\rm{L}}6}$ where *p* is the SLM pixel pitch and *f*L4, *f*L5, *f*L6 and *f*TL are the focal lengths of L4, L5, L6 and the tube lens, respectively).

In the single FOV 3D-SHOT mode, we experimentally obtained an SLM FOV size matching the theoretical expectations (950 µm × 990 µm). In the random-access mode, the maximum SLM stimulation FOV we obtained on our system is about 700 µm × 700 µm. For our experiments, we calibrated the system on a 400 µm × 400 µm FOV, as this range provided the best compromise for high diffraction efficiency and minimal aberrations. In practice, we used a 350 µm × 350 µm grid, given that this corresponded to rounded galvo voltage offsets (350 µm in our system maps to 0.5 V galvo offset), which simplified the experimental pipeline.

The reduction in FOV size and stronger off-axis aberrations in the random-access mode are probably caused by a combination of factors: addition of extra lenses (out-of-4*f* beam reducer, commercial scan lens with limited aperture), the large size of the galvo set, which introduces some inherent beam walk-off, and residual conjugation mismatches imposed by steric constraints.

In our experiments, we used a 350 µm × 350 µm grid partitioning to maintain diffraction efficiency. Users who need a larger stimulation area can either interleave holograms at high speed between adjacent FOVs or use the full 700 µm × 700 µm FOV and compensate for the low diffraction efficiency spots with increased laser power.

### Hologram characterization and system calibration

To characterize the generated holograms, 2p fluorescence was recorded from a thinly coated fluorescent slide with a substage objective (×4, Zeiss) coupled to a camera (Basler) with an *f* = 125 mm achromatic doublet, which provided a 1.3 × 1.3 mm characterization FOV. The whole substage system (fluorescent slide, objective tube lens, camera) is mounted on an XYZ motorized stage (Sutter Instruments) to enable hologram characterizations without moving the mesoscope. For successful multiphoton optogenetics experiments, precise co-alignment of the imaging and the photostimulation beams is required. To achieve this precision, hundreds of holograms were generated in 3D, and any arbitrary curvature or inhomogeneity on the imaging remote-focusing planes or the SLM planes was taken into account in the computation of the hologram-to-real-space transform matrix (Supplementary Fig. 1), in a fully automated procedure^5^. The holographic FOV was then remapped onto the entire mesoscopic FOV so that photostimulation targets could be flexibly selected in any imaging ROI configuration. Given that the nominal accessible FOV of the mesoscope spans 5 mm × 5 mm and acquisitions are performed in a random-access manner, appropriate coordinate transformations are required to convert relative positions within a given imaging ROI to absolute spatial locations within the full mesoscopic FOV. The recorded holograms were also used as a reference to correct for diffraction efficiency fall-off and deliver homogeneous powers across the FOV in vivo. To characterize hologram position invariance with translation and rotation, 2D point-cloud holograms were generated to ‘burn holes’ (that is, photobleach a fluorescent slide). The 2p fluorescence signal on the slide was then recorded with the mesoscope photomultiplier tubes (PMTs).

To characterize the impact of galvanometer scanning on hologram calibration for large-FOV photostimulation, we first recorded 3D point-cloud holograms at the (0 V, 0 V) galvanometer configuration (central FOV), and then re-recorded the same holograms while varying galvanometer positions (Supplementary Fig. 8) and translating the substage system accordingly. We found that the absolute hologram coordinates shifted linearly with galvanometer deflection, indicating that the SLM-to-real-space calibration established at the central FOV can be extended to other mesoscopic FOVs with a simple global offset adjustment. For each off-axis FOV, lateral offsets were determined by targeted photobleaching on a fluorescent slide (hole burns).

### Visual stimulation and retinotopy

Visual stimuli were displayed on a 2,048 × 1,536 Retina iPad LCD monitor (Adafruit Industries) placed 10 cm from the right eye of the mouse and computed using Psychtoolbox-3 and custom MATLAB code. Visual stimuli were presented to map retinotopy and to identify functionally defined ensembles (Fig. 3d–h). In Figs. 3a,b, 4 and 6, no visual stimulus was presented to the mouse during simultaneous imaging and photostimulation experiments. In the functional connectivity experiments (Fig. 5), four out of the six datasets were collected without concurrent visual stimulation and the remaining two were collected with simultaneous visual and holographic stimulation.

Retinotopy mapping was used primarily to identify visual cortical area boundaries. To map retinotopy, we adapted a previously published procedure^56,57^ for the 2p mesoscope (Supplementary Fig. 11). In brief, we imaged a 3.1 ×3.1 mm FOV 100–200 µm below the dura at 3 Hz when mice were viewing noise bars drifting in the horizontal or vertical directions. The bars were 10 degrees in width and spanned the vertical or horizontal extents of the screen, respectively. The noise was drawn from a Gaussian distribution with a spatial frequency of 0.03 cycles per degree and a temporal frequency of three cycles per degree. Each type of bar swept the screen 30 times with a period of 9 s, for a total stimulation duration of 10 min. We smoothed the recorded 2p calcium frames with a four-to-eight-pixel square filter and downsampled them four to eight times. For each pixel in the reduced image, we computed the preferred azimuth and elevation by identifying the average horizontal and vertical positions of the bar at which the pixel’s response was maximal. This generated azimuth and elevation preference maps for the full FOV. We then computed the sign of the gradients between the two maps to generate a visual field sign map^58^ and used sign flips in this map to identify area boundaries^59,60^.

### In vivo mesoscale 2p imaging and photostimulation

For the in vivo 2p mesoscale calcium imaging, mice were head-fixed and allowed to run on a freely moving circular treadmill. Mesoscale Imaging ROIs were set to 2.4 mm × 2.4 mm to 3.6 mm × 3.6 mm FOVs, with recording rates between 3 Hz and 5 Hz. The imaging laser was set at 920 nm. Opsin-expressing cells were automatically segmented online based on either the green channel signal (Ai203 mice or transgenic mice virally injected with ChRmine-GCaMP6m) or the red nuclear mRuby2 (virally injected mice with ChroME2s-mRuby2). To minimize the photostimulation artefact induced by the stimulation laser on the imaging, we use an Arduino Mega 2560 board to gate the photostimulation laser on the imaging resonant galvanometer and only stimulate at the edge of the resonant galvanometer scan. The corresponding edge stripes are excluded from analysis. Despite this gating strategy, given the large detection etendue of the 2p-RAM mesoscope, we noticed that multi-target photostimulation generates an artefact on the imaging outside of the gating window when using mice expressing red fluorescent proteins. For these experiments (Fig. 3), we conservatively subtracted post-stimulus frames for analysis and only used the tail of the calcium traces for analysis. This caveat is completely solved when using mice not expressing any red fluorescent protein (Figs. 4–6), and the full trace can be used for analysis. Experiments shown in Figs. 2 and 3 were performed with a standard Hamamatsu H10770A (PMT). Experiments shown in Figs. 4–6 were performed with a gated PMT (H11706-40, Hamamatsu) used in non-gated mode.

### Optical PPSF measurements

To estimate the PPSF in the Ai203; *Vglut*-Cre mouse, we first identified photoactivatable neurons near the center of the holography FOV and selected eight targets for multi-target stimulation. We generated 50 eight-target holograms around and including the central holograms: 17 for the lateral PPSF and 33 for the axial PPSF. For the lateral PPSF, each hologram was offset in the [−40,40] μm range with 5 μm increments along the X axis. For the axial PPSF, each hologram was offset in the [−80,80] μm range with 5 μm increments along the Z axis. Each hologram was illuminated with five 5 ms pulses delivered at 30 Hz and 25 mW per target, with ten repeats per hologram. The responses of the targeted neurons were averaged over each condition’s repeats to compute the PPSF.

For the PPSF experiment sessions in mice virally expressing the ChRmine opsin, we first ran a photoactivatability experiment at a series of powers (range, 0.5–10 mW per target) on a set of 40–80 putative opsin-expressing neurons with the multi-target hologram centered (that is, lateral and axial offsets set to zero). These experiments were performed with the 2p-RAM configuration with galvos set at the central (0,0) position, which we have shown generates similar optical PSFs to the no-galvo 3D-SHOT implementation (Fig. 4). The stimulation was delivered as a train of ten 10 ms pulses at 30 Hz. We computed the power-response relationship for individual neurons and selected 10–20 neurons activated at intermediate powers and exhibiting a high dynamic range of power responses. We then performed the PPSF experiment on this subset of neurons with the corresponding holograms shifted laterally (range, −40 to 40 µm in 5 µm increments) or axially (range, −80 to 80 µm in 10 µm increments), and at low (0.5–2 mW per cell), intermediate (2–5 mW per cell) and high (5–10 mW per cell) powers, repeated ten times for each offset–power combination. For each neuron’s ROI, the extracted calcium fluorescence traces were baseline-subtracted, and the evoked response was computed by averaging the Δ*F* in a 1.5 s window following stimulation onset for each condition. Computed responses at different spatial offsets were also normalized to the centered (zero offset) condition at each power and averaged across neurons. We then fit a weighted Gaussian to the mean spatial profile of responses to compute the PPSF metrics at each power.

### Multi-area targeting with single FOV 3D-SHOT

For the multi-area holography demonstration shown in Fig. 2f, we first identified area boundaries using retinotopy, and picked four visual cortical areas (V1, PM, RL and LM) for same-session sequential targeting. For each area, we moved the objective along both lateral axes until the holographic FOV (fixed with respect to the objective) was positioned over the center of the area. We then adjusted the 2p-RAM scan fields to image a contiguous 3.1 mm × 3.1 mm area covering a fixed portion of the window using the vasculature as fiduciary markers. As a result, at the beginning of each area’s experiment, the imaging FOV was identical but the holography FOV covered a different cortical area. In each experiment, we identified 10–15 of the most photoactivatable neurons with sequential single-neuron targeting. We then targeted these neurons (V1-13, PM-12, RL-11, LM-12 neurons) in each area using a multi-target hologram, driving them with a single 250 ms pulse and 80 mW per target. This configuration requires repositioning the mouse and post hoc registration of the imaging FOV, making it well-suited for experiments that involve probing different cortical areas across separate sessions.

### Holographic stimulation of random multi-target ensembles

For the targeted photostimulation experiments presented in Fig. 3a,b, we used *Vglut1*-Cre; Ai203 mice. We recorded four sessions from two female mice (*n* = 2 sessions each). The holographic FOV was placed over LM. The imaging FOV encompassed V1, LM, RL, anterolateral area (AL), anterior area (A) and anteromedial area (AM) (3,000 µm × 1,800 µm). Each target received 50 mW, and each stimulation consisted of ten pulses of 10 ms duration at 16 Hz. In each session, four distinct holograms were used in addition to a zero-power condition, with 80 trials per condition.

### Decoding photostimuli pairs

To discriminate between pairs of distinct photostimulation patterns, a gradient boosting decision tree algorithm was used. The classifier was trained on 70% of the trials (*n* = 112 trials per pair labeled as either photostimulus 1 or photostimulus 2) and tested on the remaining trials (*n* = 48 trials). The decoding accuracy was defined as the number of correctly predicted trials over the total number of test trials. For each pair of photostimuli, the final reported decoding accuracy result was the average across ten independent draws (cross-validation). For the shuffled control decoding, the classifier was trained on randomly mislabeled trials and tested on the 30% held-out trials.

### Holographic stimulation of functionally defined ensembles

For the co-tuned ensemble stimulation experiments presented in Fig. 3, we used *Vglut1*-Cre; Ai203 mice (Ai203 is a transgenic line expressing the transgene TITL-st-ChroME-GCaMP7s-ICL-nls-mRuby3-IRES2-tTA2 (ref. ^19^)). We recorded 11 sessions from two female mice (five and six sessions for each mouse). The holographic FOV was placed over V1 (white rectangle in Fig. 3a). The imaging FOV encompassed V1, LM, RL, AL, PM and AM (2,400 µm × 2,400 µm). The imaging and holographic FOV was fixed throughout each recording session.

To holographically stimulate neural ensembles defined by their visual response properties, each recording session consisted of three steps. In step 1, we recorded visual responses of neurons in the FOV while the mouse viewed various images on the monitor. To gauge the orientation preference of each neuron, we presented static gratings in four different orientations (0, 45, 90, 135°), at a contrast of 0.75 and a spatial frequency of 0.04 cycles per degree. Gray screen ‘blank’ trials, as well as static gratings in each orientation, were repeated 50 times, with randomized trial order. Each presentation, or trial, lasted for 1 s with 0 s inter-trial intervals. As such, the static grating block lasted 250 s (five trial types with 50 repeats of 1 s duration).

In step 2, we analyzed the 2p imaging data collected in step 1 by running Suite2p^61^ on the computer running ScanImage. Before running Suite2p, we converted the ScanImage tif files into an h5 file by loading the ScanImage tif files, cropping them into a region corresponding to the holographic FOV, keeping only the frames corresponding to the functional channel (for example, every other frame if both red and green PMTs were recorded) and then saving as an h5 file. It was important to convert at least 5,000 time points because Suite2p identifies many more spurious ROIs with shorter recordings. Next, we ran Suite2p on the cropped h5 file. As there is insufficient time to manually curate the ROIs during the online analysis, we analyzed tuning curves for every ROI returned by Suite2p.

In step 3, we holographically stimulated neural ensembles defined in step 2. Each target received 50 mW, and each stimulation consisted of ten pulses of 10 ms duration at 16 Hz. In addition to the co-tuned ensemble stimulation at four orientations, zero-power ‘blank’ trials and mixed-tuned ensemble stimulation trials were interleaved (that is, six trial types). Each trial type was repeated 20 times with an inter-trial interval of 5 s.

To select tuned target neurons to stimulate, we use the area under the receiver operating characteristic curve (AUROC), discriminating between responses to preferred versus orthogonal orientations. Another commonly used metric to characterize orientation selectivity is the orientation selectivity index (OSI), which is typically defined as (*R*_pref_ − *R*_orth_) / (*R*_pref_ + *R*_orth_), where *R*_pref_ and *R*_orth_ are the mean responses at the preferred and orthogonal orientation, respectively. However, because we *z*-scored and baseline-subtracted the calcium fluorescence traces for each cell, *R*_pref_ and *R*_orth_ values could be negative. Moreover, when the denominator *R*_pref_ + *R*_orth_ is close to zero, OSI could have an arbitrarily high value. To circumvent these issues with OSIs, we leveraged the AUROC metric, separating responses to preferred and orthogonal orientations. We compared this metric to the adjusted OSIs, in which visual responses were offset by 1 to avoid negative values. Adjusted OSI is hence defined as OSI_adj_ = $\frac{{R}_{\mathrm{adj\_pref}}-{R}_{\mathrm{adj\_orth}}}{{R}_{\mathrm{adj\_pref}}+{R}_{\mathrm{adj\_orth}}}$, where *R*_adj_pref_ = *R*_pref_ + 1 and *R*_adj_orth_ = *R*_orth_ + 1. We found that both AUROC values and adjusted OSI values are significantly higher for the targeted neurons compared to other V1 neurons (Extended Data Fig. 5). We also found that AUROC and adjusted OSI values are highly correlated with one another (Extended Data Fig. 5).

### Functional connectivity experiments

In the functional connectivity experiments (Fig. 5), four datasets were acquired without visual stimulation (gray or black screen), and two with visual stimulation consisting of full-screen Gaussian-modulated noise patterns at 50% contrast. For these experiments, visual stimulation was triggered synchronously with optogenetic stimulation and was kept on the screen for the remaining trial duration. Data were collected across four independent sessions in two mice. In two of these sessions, photostimulation was performed under both visual and non-visual conditions in a randomized trial structure. For analysis, visual and non-visual trials were treated as separate sessions. For these experiments, we used virally injected transgenic *CaMK2*-tTA; tetO-GCaMP6s mice injected with pAAV-hSyn-GCaMP6m-p2A-ChRmine-Kv2.1-WPRE. Cells with elevated green baseline fluorescence were identified as putative opsin-expressing neurons and selected as photostimulation targets. Compact neural ensembles were selected within LM and V1, with each ensemble assigned a specific SLM phase mask and a set of discrete XY galvanometer positions. The corresponding phase mask and galvo coordinates were updated before each trial start. Imaging was performed at 3 Hz over a 3,100 µm × 3,100 µm FOV encompassing eight contiguous visual areas (V1, LM, AL, RL, PM, AM, medio-medial-anterior area (MMA), medio-medial-posterior area (MMP)), as well as surrounding regions. For functional connectivity experiments presented in Supplementary Fig. 13, we used virally injected transgenic mice (*Emx1*-Cre; *CaMK2*-tTA; tetO-GCaMP6s injected with AAV9-CAG.DIO.ChroME2s-FLAG-ST.P2A.H2B.mRuby3.WPRE.SV40). The holographic FOV was placed over LM. The imaging FOV encompassed V1, LM, RL, PM and AM (2,400 µm × 2,400 µm). Stimulation consisted of long 250 ms pulses, at 0 mW, 30 mW and 50 mW, with 200 trials per power condition to maximize detectability of long-range responses. The 30 mW and 50 mW trials were averaged as the stimulation condition. Functional connectivity metrics separating visual and non-visual conditions are presented in Supplementary Figs. 14 and 15.

### Rapidly interleaved photostimulation of remote ensembles

In rapidly interleaved remote photostimulation experiments (Fig. 6), putative opsin-expressing cells were segmented across the full 3.1 mm × 3.1 mm mesoscopic FOV, as in the inter-areal functional connectivity experiments. Each cell was assigned a specific XY galvanometer position, and neural ensembles were then defined and paired with a corresponding SLM phase mask and XY galvo coordinate. For rapidly interleaved photostimulation, two spatially remote ensembles were targeted by synchronizing SLM phase mask updates with XY galvo movements. The minimal galvo travel time between positions was calculated based on the specified galvo speed (1.2 ms per 0.4° optical deflection for QS30XY-AG Thorlabs scanners, which translates into a 53 µm ms^−1^ speed, therefore 19 ms to travel 1 mm in cortical space). An additional 5 ms buffer was added to define the final time interval between phase mask switches. Photostimulation consisted of ten pulses (10 ms, 10 Hz) with 50 trials per condition.

### Offline analysis of mesoscopic 2p calcium data

Motion correction and source extraction were performed using Suite2p^61^, run through a GPU for faster processing. Calcium sources were manually curated based on morphologically identifiable neurons. In the experiments shown in Figs. 2 and 3, frames with stimulation laser artefacts were discarded for downstream analysis, and neuropil fluorescence was subtracted from the fluorescence traces using a 0.7 neuropil coefficient. Each cell’s fluorescence trace was then *z*-scored (Fig. 2) or the Δ*F*/*F* was computed before *z*-scoring (*z*-scored Δ*F*/*F*). Δ*F*/*F* was defined as (*F* − *F*_med_) / *F*_med_, where *F* is the neuropil-corrected fluorescence trace and *F*_med_ is the median of the neuropil-corrected fluorescence trace across all trials. A baseline of two to five frames before the stimulus onset was then subtracted for every trial (Figs. 3 and 4). Photostimulation target coordinates were remapped into the stitched mesoscale FOV and assigned to the closest suite2P-identified calcium source. In experiments shown in Figs. 4–6, there was no stimulation laser artefact except at the scanning stripe edges; therefore, all frames were kept for analysis. Fluorescence traces were z-scored across the entire experiment, and two frames before the stimulus onset were subtracted for every trial. We found that neuropil subtraction had minimal impact on the causal influence maps (Supplementary Fig. 16). For functional connectivity mapping experiments (Figs. 5 and 6), no neuropil correction was used.

### Definition of modulated and follower cells

To quantify the impact of holographic perturbation across cortical areas, we classified cells based on their trial-by-trial modulation following photostimulation. Modulated cells were defined as cells located >100 µm from the nearest photostimulation target that exhibited a significant change in post-stimulus activity compared to no-power (0 mW) control trials (two-sided Mann–Whitney *U*-test, *P* < 0.05). This category includes both strongly and weakly affected cells and captures the broader network influence of the stimulation. Follower cells were defined as a subset of modulated cells exhibiting high response consistency across trials. To identify them, photostimulation trials were randomly split into two non-overlapping subsets. Modulated cells were independently identified within each split using the same significance criterion (*P* < 0.05). Follower cells were defined as the intersection of the significantly modulated populations across both trial splits. This approach selects cells with consistent stimulus-evoked modulation and minimizes the inclusion of false positives driven by trial-to-trial variability.

### Functional connectivity metrics

The modulation strength metric computed in Fig. 5d quantifies the average amplitude of photostimulation-induced responses among follower neurons. It is computed as the sum of the absolute modulation amplitudes divided by the number of follower cells, *N*_mod_ (modulation strength = $\frac{{\sum }_{i=1}^{n}|{R}_{i}|}{{N}_{\mathrm{mod}}}$). This provides a polarity-independent measure of the average effect size per modulated neuron, allowing comparison of response strength across conditions or cortical areas regardless of the number of recruited cells. The net influence metric computed in Fig. 5 quantifies the net directional impact of photostimulation on a downstream area. It is calculated as the sum of the signed modulation amplitudes across all follower cells, divided by the total number of follower cells (net influence = $\frac{{\sum }_{i=1}^{n}{R}_{i}}{{N}_{\mathrm{mod}}}$). Positive values indicate a net excitatory effect, and negative values indicate a net inhibitory influence. Additional functional connectivity metrics are defined in Extended Data Fig. 10. These metrics were also calculated using a broader pool of putative connections (Extended Data Figs. 8 and 10).

### Statistics and reproducibility

All statistical analyses were performed using Python or MATLAB. The analyses performed were paired *t*-test, Wilcoxon rank-sum and Mann–Whitney *U*-test; tests were two-sided unless stated otherwise. No statistical method was used to predetermine sample size. Trial randomization was used in all applicable experiments (Figs. 2–6). Statistical analyses on functional connectivity metrics are described in detail in Supplementary Note 4 and Supplementary Table 3.

Micrographs shown in Figs. 2a, 3c and 4g are representative of experiments repeated several times across users and mouse preparations. To date, more than 100 independent mesoscale holographic experiments have been performed with the system.

### Reporting summary

Further information on research design is available in the Nature Portfolio Reporting Summary linked to this article.

## Online content

Any methods, additional references, Nature Portfolio reporting summaries, source data, extended data, supplementary information, acknowledgements, peer review information; details of author contributions and competing interests; and statements of data and code availability are available at 10.1038/s41593-026-02350-9.

## Supplementary information

Supplementary Information Supplementary Figs. 1–16, Supplementary Notes 1–4, Supplementary Tables 1–3, Supplementary References.

Reporting Summary

Supplementary Data CAD file of holographic mesoscope design.

## Source data

Source Data Fig. 1 Statistical Source Data

Source Data Fig. 2 Statistical Source Data (MATLAB)

Source Data Fig. 3 Statistical Source Data (XLSX + MATLAB)

Source Data Fig. 4 Statistical Source Data

Source Data Fig. 5 Statistical Source Data

Source Data Fig. 6 Statistical Source Data

Source Data Extended Data Fig./Table 2 Statistical Source Data

Source Data Extended Data Fig./Table 3 Statistical Source Data

Source Data Extended Data Fig./Table 4 Statistical Source Data (Matlab)

Source Data Extended Data Fig./Table 5 Statistical Source Data (Matlab)

Source Data Extended Data Fig./Table 7 Statistical Source Data

Source Data Extended Data Fig./Table 8 Statistical Source Data

Source Data Extended Data Fig./Table 9 Statistical Source Data

## Data Availability

Relevant data for this manuscript are available as source data files. Source data are provided with this paper.

## Code availability

Custom MATLAB (v.2021-2024) and Python (v.3.12-3.14) code to run and analyze holographic mesoscale experiments is available at https://github.com/mesoholo/mesoholo.
