# Supplementary material for: Probing inter-areal computations with a two-photon holographic mesoscope
Source: Nat Neurosci. 2026 Aug 3;29(8):2023–35. doi: 10.1038/s41593-026-02350-9 (PMC13433287; doi:10.1038/s41593-026-02350-9)
Supplement: Supplementary file 1 [file 41593_2026_2350_MOESM1_ESM.pdf]

nature neuroscience

Supplementary information

https://doi.org/10.1038/s41593-026-02350-9

Probing inter-areal computations with a two-photon holographic mesoscope

In the format provided by the authors and unedited

Probing inter-areal computations with a two-photon holographic mesoscope

Lamiae Abdeladim[1,3* †], Uday K. Jagadisan[1,3*], Hyeyoung Shin[1,3,4*], Mora B. Ogando[1,3], Hillel Adesnik[1,2,3 †]
[1]Department of Neuroscience, University of California, Berkeley
[2]The Helen Wills Neuroscience Institute
[3]Department of Molecular and Cell Biology, University of California, Berkeley
[4]School of Biological Sciences, Seoul National University, Seoul, Republic of Korea
* Equal contributions
† Corresponding authors: Lamiae Abdeladim, Hillel Adesnik.
Contact: lamiae.abdeladim@gmail.com, hadesnik@berkeley.edu

Supplementary Information

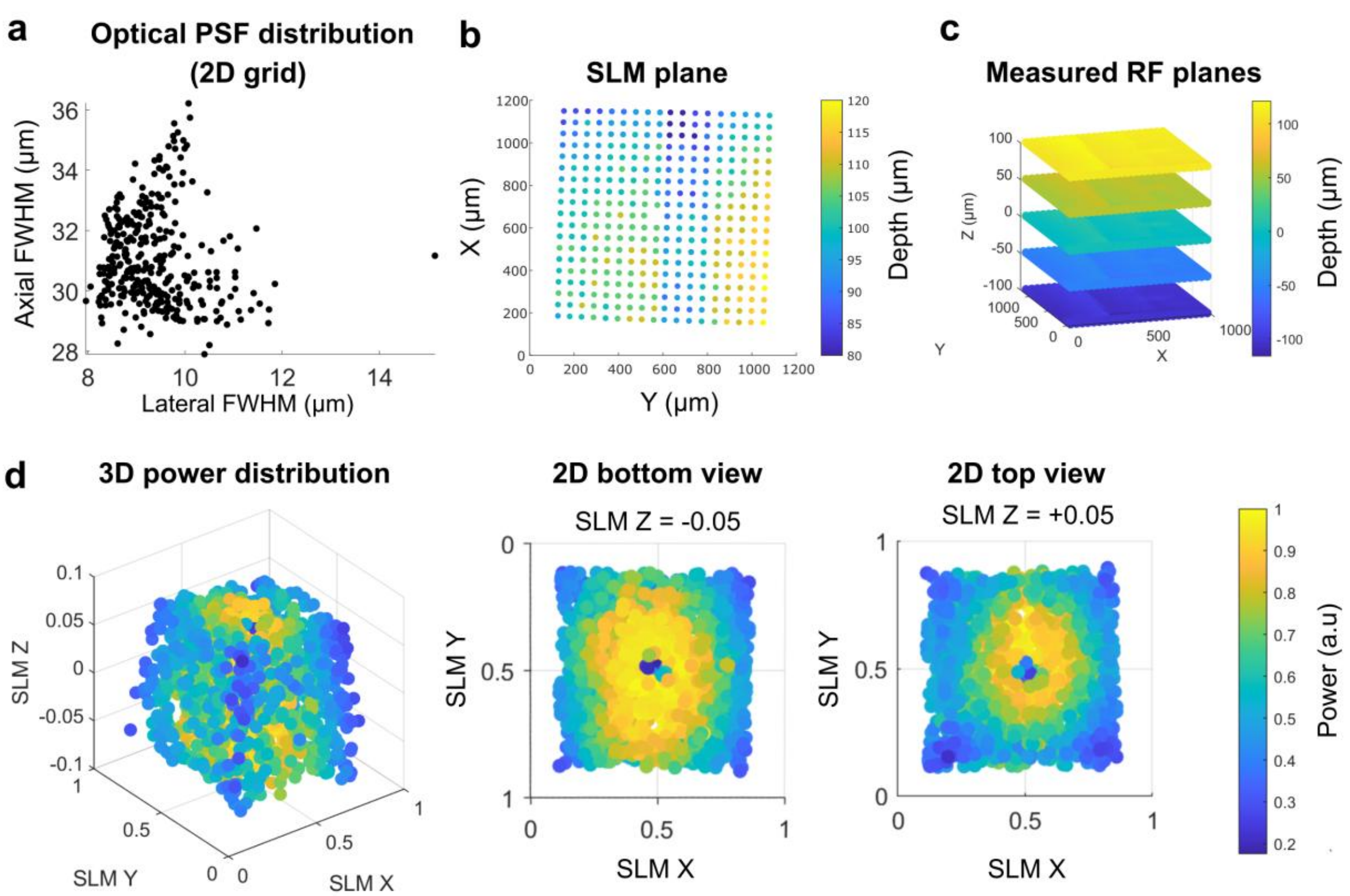

Supplementary Figure 1. Optical characterization of the 2P holographic mesoscope. a) Distribution of optical point-spread-functions of holograms arranged in a 2D grid across the holographic FOV (n=538 holograms). **b)** Axial locations in real space (units: microns, arbitrary origin) of holograms with equi-Z SLM coordinates across the holographic FOV (n=538 holograms). **c)** 3D remote-focusing fluorescence imaging planes in real space measured with an inverted substage camera with voice-coil curvature correction on. The recorded holograms are registered to these recorded planes for accurate 3D calibration. **d)** Power

distribution variation in 3D measured as the normalized square-root of the 2P fluorescence signal for a distribution of holograms randomly positioned in 3D space. This distribution is used to build a 3D model of diffraction efficiency that is used to dynamically correct the power allocated to each hologram as a function of its location in space, thus allowing to use a greater range of the SLM. Right: 2D views of the power distribution from the top (SLMz = +0.05) and the bottom (SLMz = -0.05) across a 200µm axial span.

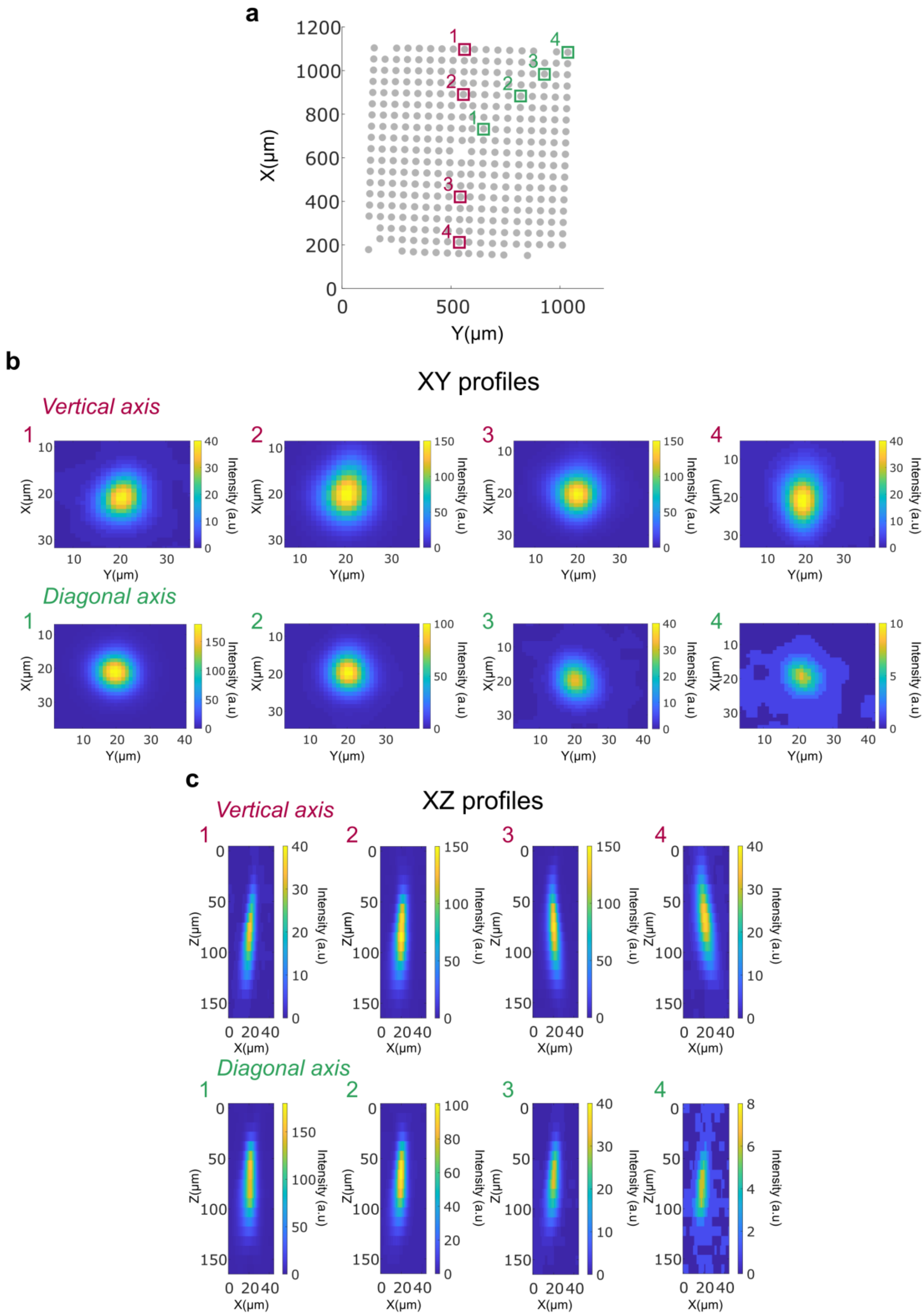
a
X(μm)
Y(μm)
b
XY profiles
Vertical axis
Diagonal axis
Intensity (a.u)
c
XZ profiles
Vertical axis
Diagonal axis
Z(μm)
X(μm)

Supplementary Figure 2. Example hologram intensity profiles across the SLM FOV. a) Detected 2D locations of holograms used for SLM FOV characterization. Cherry and green boxes indicate the locations of the select examples of off-axis holograms shown below. **b)** Lateral 2P fluorescence intensity profiles at select off-axis SLM FOV locations. **c)** Axial 2P fluorescence intensity profiles at select off-axis SLM FOV locations.

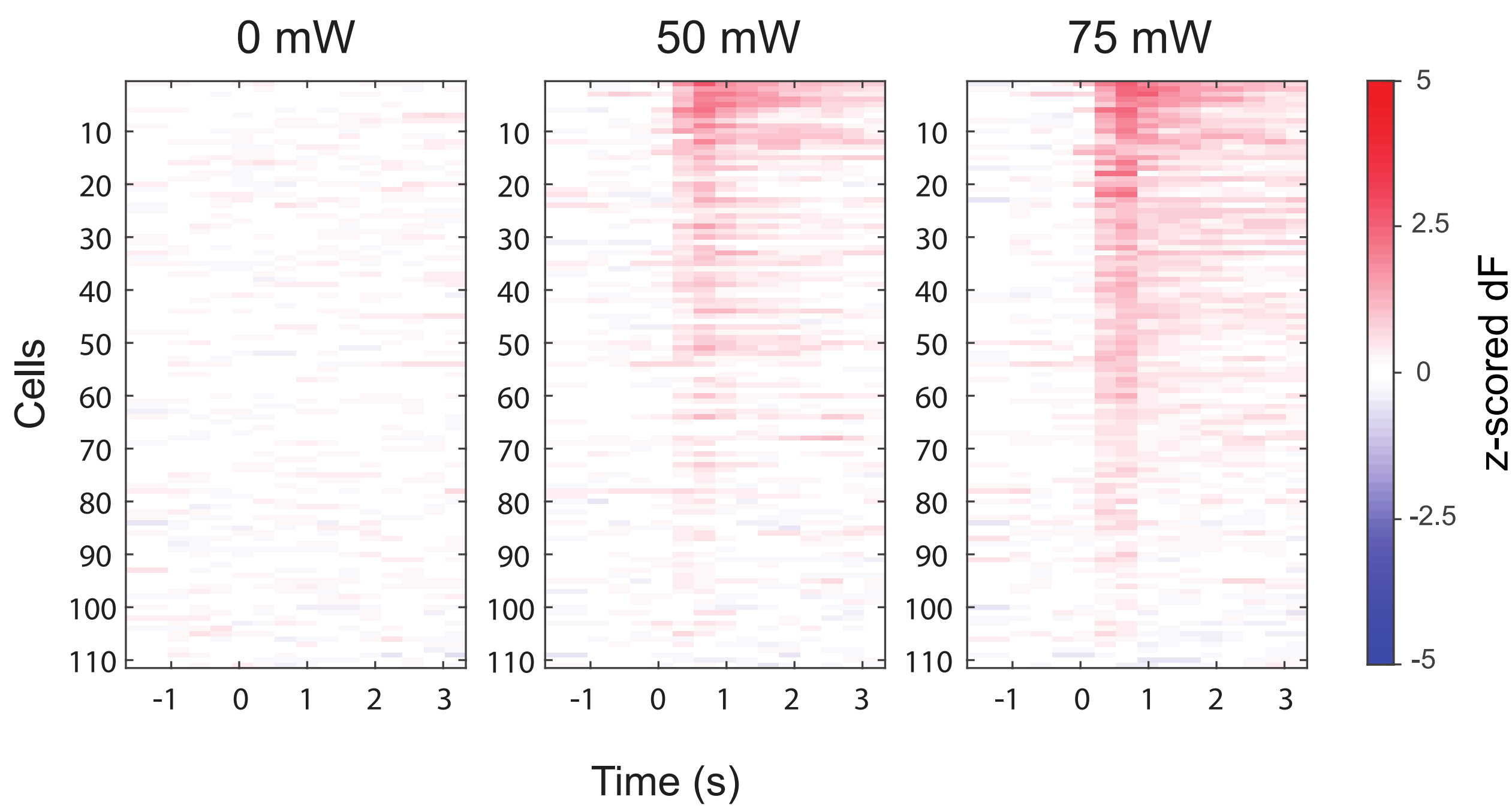

Supplementary Figure 3. Sequential stimulation at different laser powers. Heatmaps of stimulus-aligned calcium responses (z-scored dF) for 111 targeted cells in an example session (Ai203 × VGlut Cre transgenic). 13, 58 and 75 were significantly activated at 0, 50, and 75mW, respectively, signed rank test, one tailed p<0.025).

a

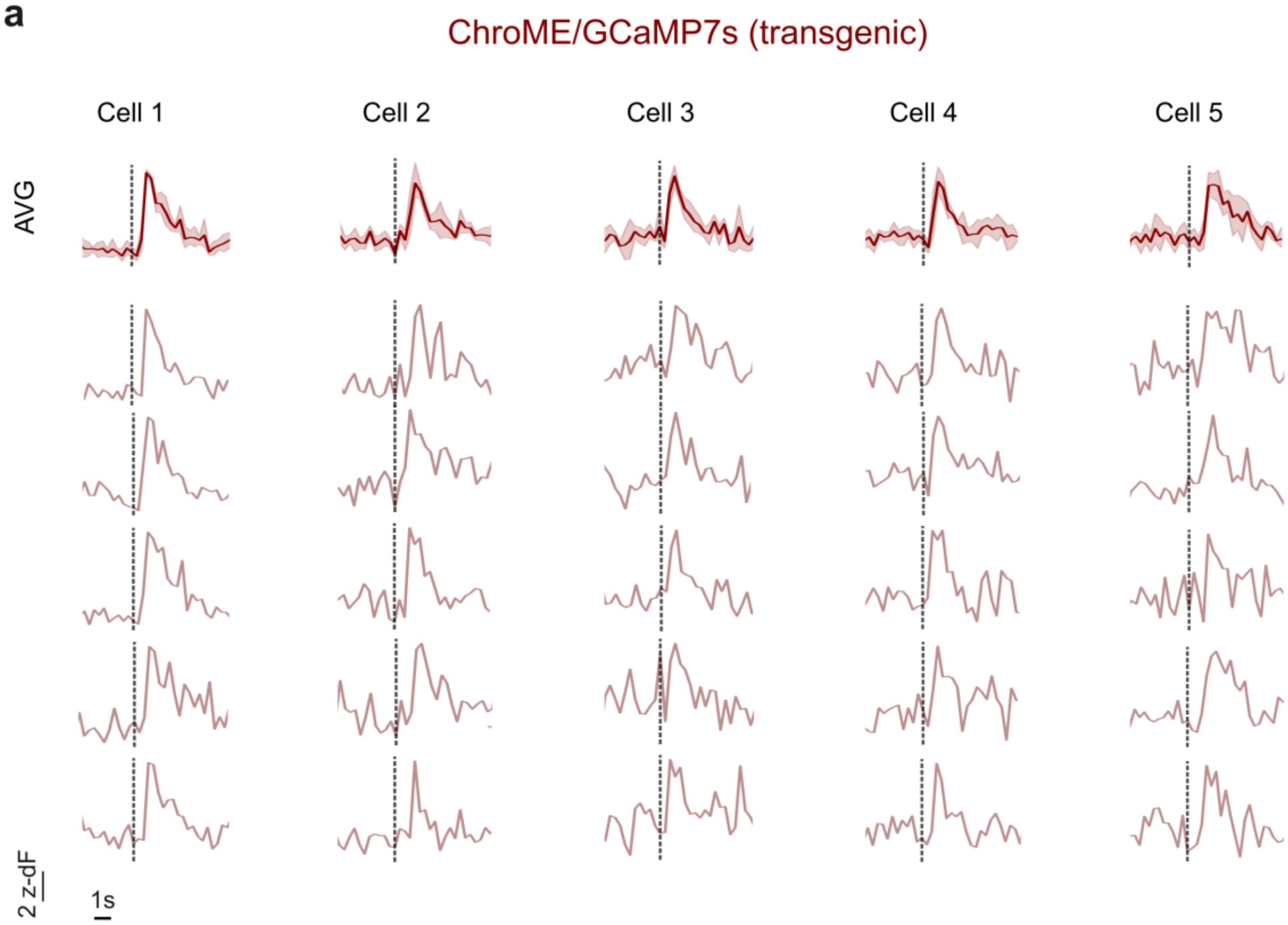

ChroME/GCaMP7s (transgenic)
Cell 1
Cell 2
Cell 3
Cell 4
Cell 5
AVG
2 z-dF
1s

b

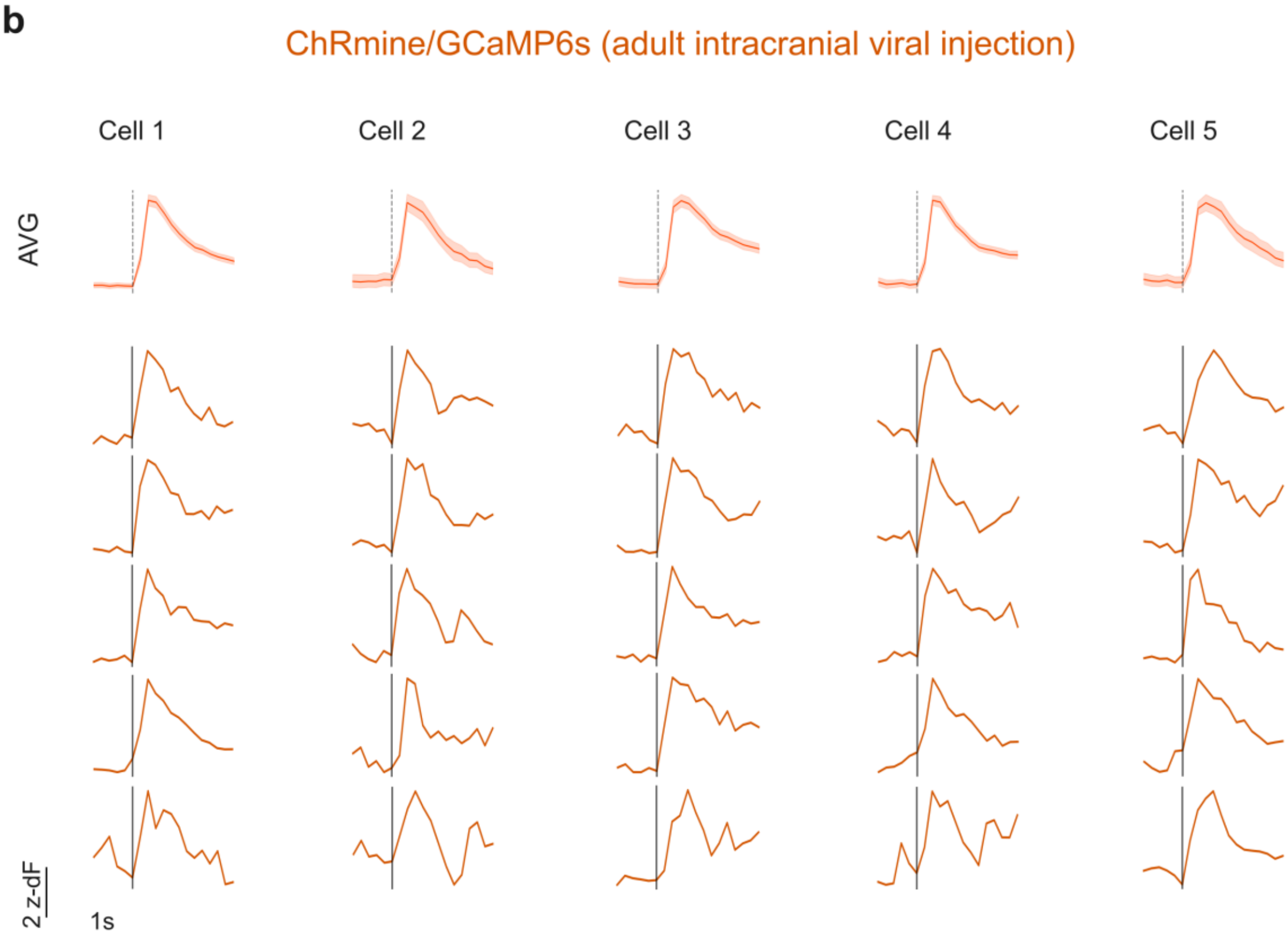

ChRmine/GCaMP6s (adult intracranial viral injection)
Cell 1
Cell 2
Cell 3
Cell 4
Cell 5
AVG
2 z-dF
1s

Supplementary Figure 4. Reliable target photostimulation across trials a) Example average (5trials) and single-trial traces across photostimulation repeats of target neurons co-expressing ChroME and GCaMP7s (Ai203xVglutCre transgenic). Photostimulation at 50mW. The shaded band denotes the bootstrapped 95% confidence interval around the mean. **b)** Example average (100 trials) and single-trial traces (5 randomly selected among 100) across photostimulation repeats of target neurons co-expressing ChRmine and GCaMP6s. Photostimulation at 10mW. The shaded band denotes the bootstrapped 95% confidence interval around the mean.

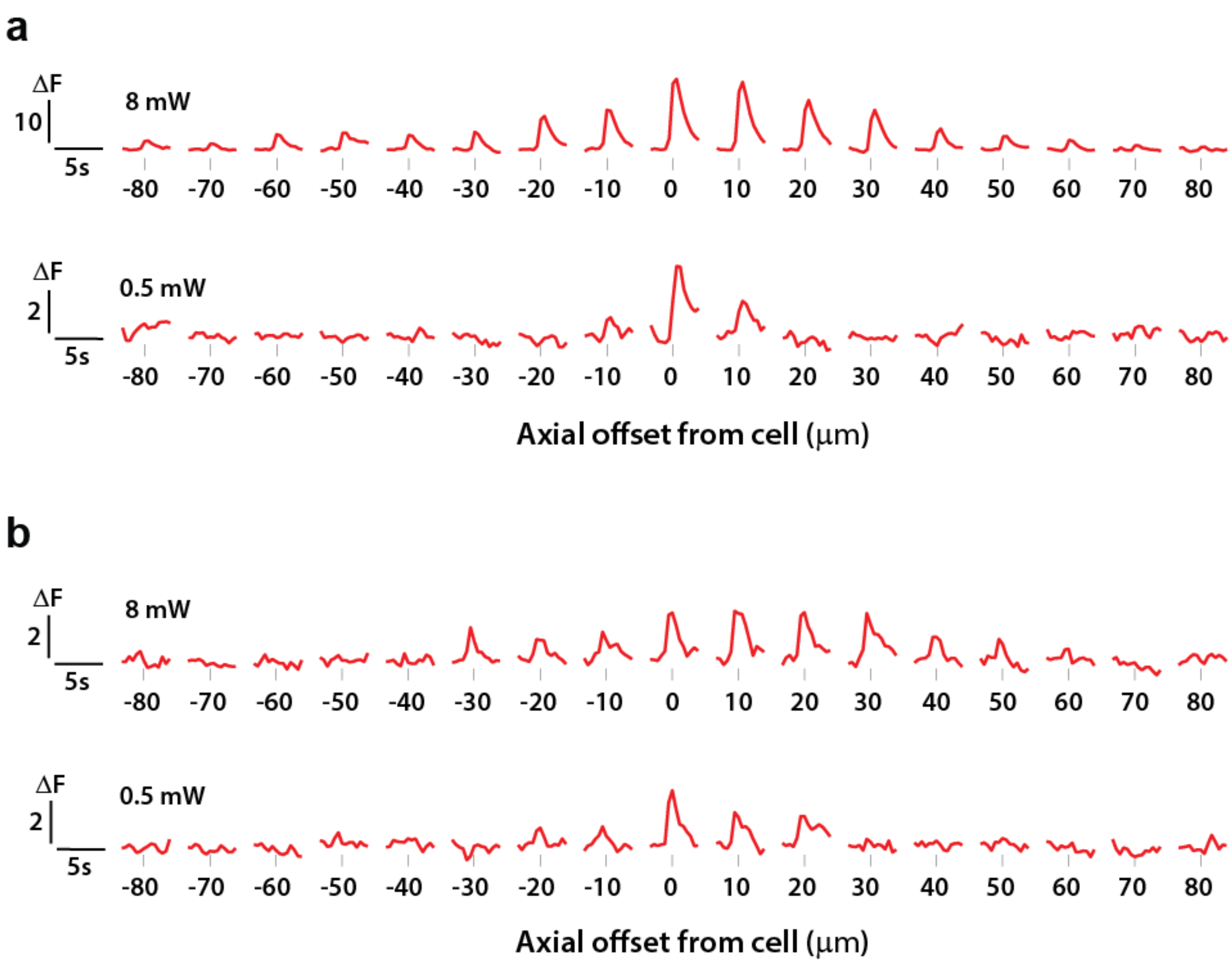

Supplementary Figure 5. Example calcium traces from PPSF experiments. a) Neural activity traces for an example cell at various axial offsets (columns), at high (first row) and low (second row) powers. **b)** Similar to a) for another example cell.

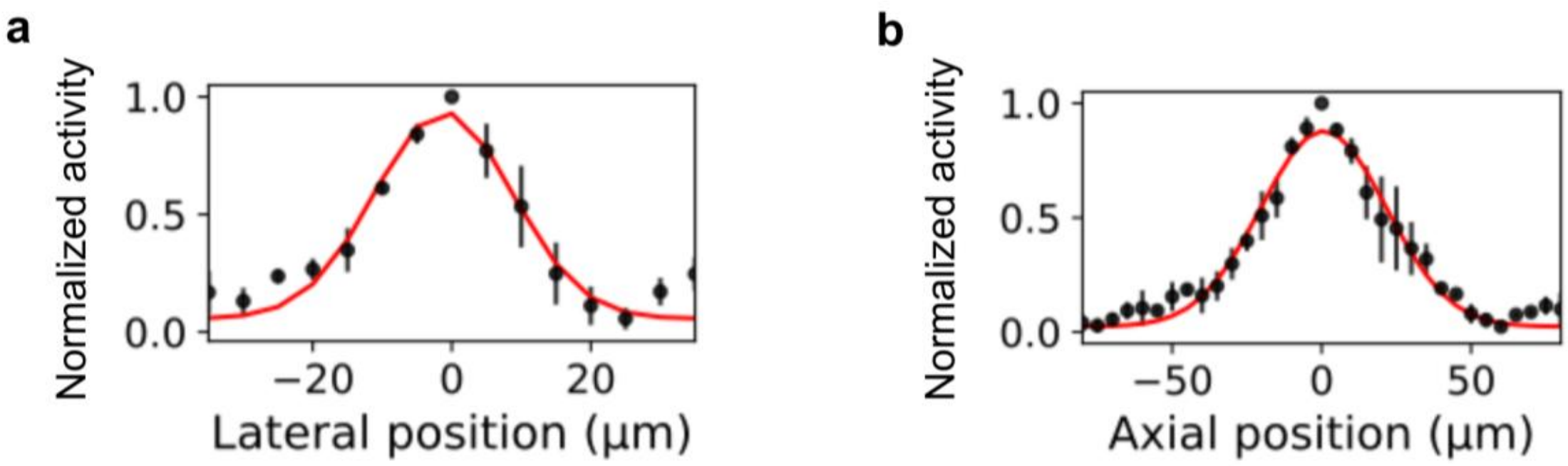

Supplementary Figure 6. Physiological point-spread-function measured in Ai203;VGlut-Cre. *In vivo* optically measured physiological point-spread functions (n=3cells). Cells co-expressing GCaMP7s and ChroME. Error bars: S.E.M. Red curve: Gaussian fit, lateral full-width-half-maximum (FWHM) 23 µm, axial FWHM: 49 µm. Photostimulation with 5x5ms pulses at 30Hz at 25mW.

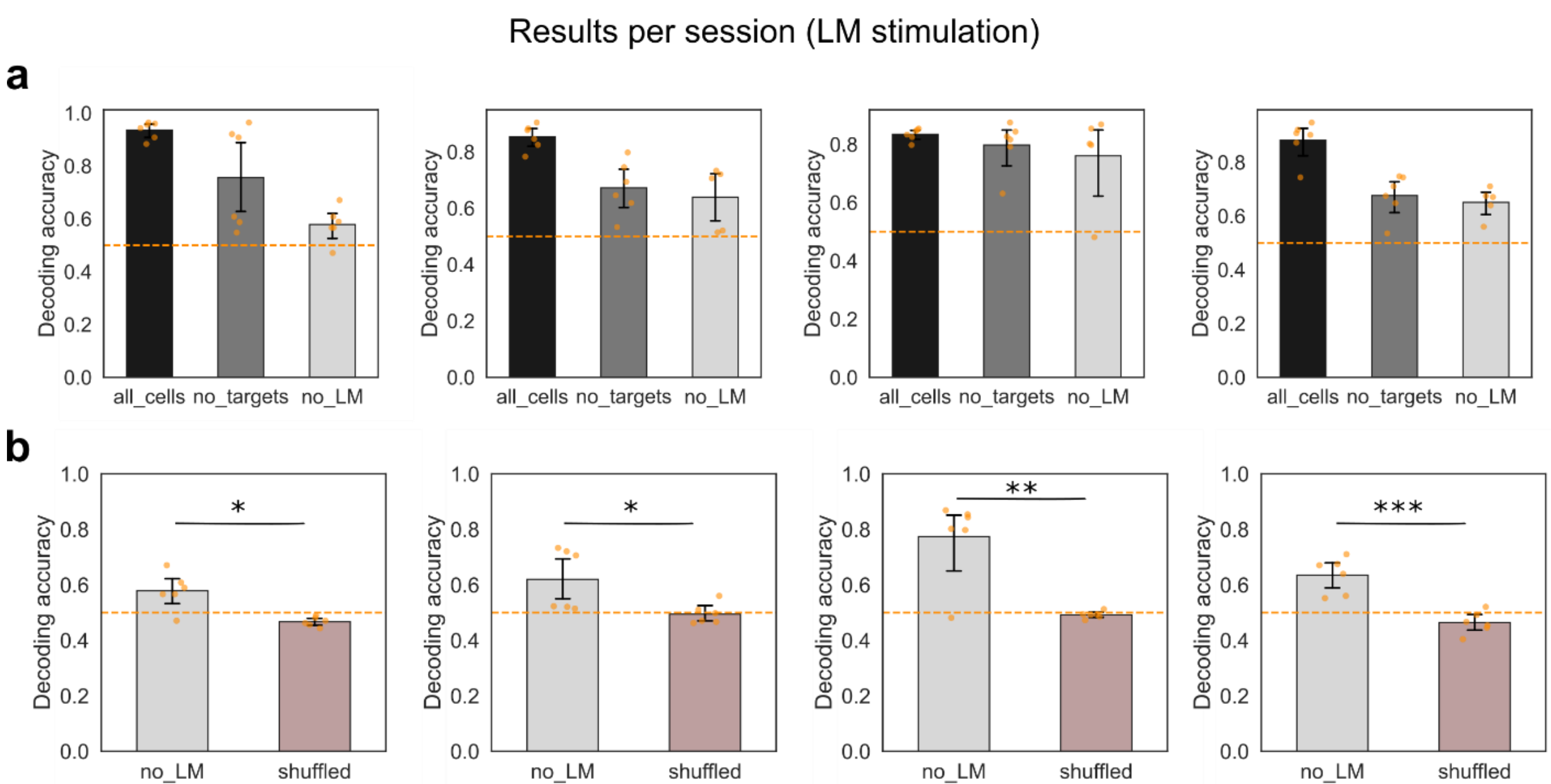

Supplementary Figure 7. Photostimulus decoding results per session. a) Decoding accuracy of a classifier trained to discriminate the holographically induced patterns in LM from different groups of non-targeted cells in the FOV, split per session. 6 pairs of stimuli are presented for discrimination in each session. All cells: decoding from all neurons in the mesoscale FOV. No targets: decoding from all neurons except the targeted cells. No LM: decoding from all neurons outside area LM. Barplots indicate mean +/- s.e.m. b) Decoding accuracy per session of a classifier trained to discriminate the holographically induced patterns in LM from all neurons outside area LM with correct and shuffled labels in the training set. Barplots indicate mean +/- s.e.m. Two-sided Wilcoxon signed-rank test (n=6): Session1/Mouse1: p = 0.013, Session2/Mouse1: p = 0.023, Session1/Mouse2: p=0.005, Session2/Mouse2: p = 0.0006.

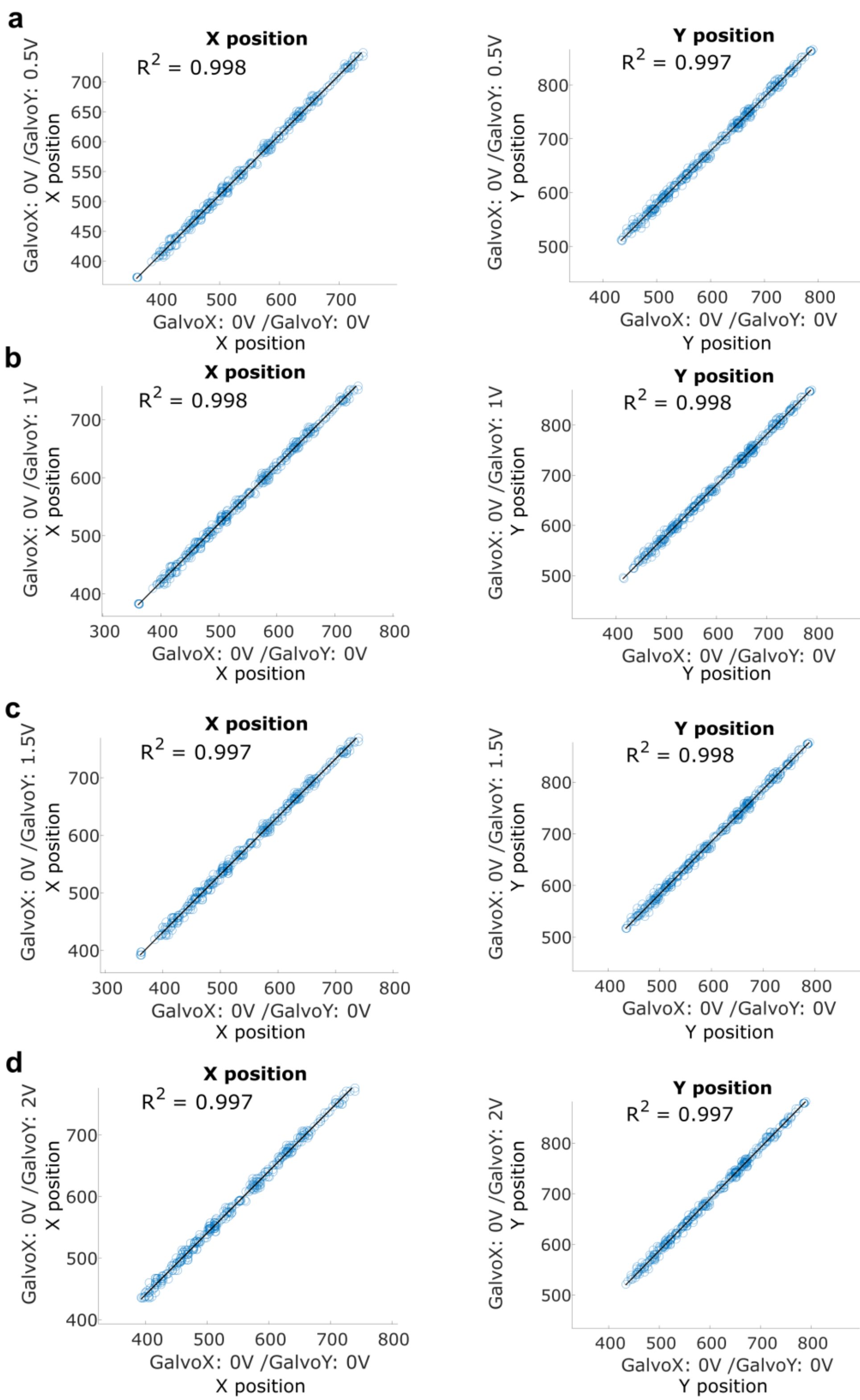
a
X position
R^2 = 0.998
GalvoX: 0V /GalvoY: 0.5V X position
GalvoX: 0V /GalvoY: 0V X position
Y position
R^2 = 0.997
GalvoX: 0V /GalvoY: 0.5V Y position
GalvoX: 0V /GalvoY: 0V Y position
b
X position
R^2 = 0.998
GalvoX: 0V /GalvoY: 1V X position
GalvoX: 0V /GalvoY: 0V X position
Y position
R^2 = 0.998
GalvoX: 0V /GalvoY: 1V Y position
GalvoX: 0V /GalvoY: 0V Y position
c
X position
R^2 = 0.997
GalvoX: 0V /GalvoY: 1.5V X position
GalvoX: 0V /GalvoY: 0V X position
Y position
R^2 = 0.998
GalvoX: 0V /GalvoY: 1.5V Y position
GalvoX: 0V /GalvoY: 0V Y position
d
X position
R^2 = 0.997
GalvoX: 0V /GalvoY: 2V X position
GalvoX: 0V /GalvoY: 0V X position
Y position
R^2 = 0.997
GalvoX: 0V /GalvoY: 2V Y position
GalvoX: 0V /GalvoY: 0V Y position

Supplementary Figure 8. Calibration linearity across holographic field-of-views. Measured X and Y hologram coordinates when the galvo pair is at the central position (GalvoX:0V/ GalvoY:0V) plotted against X and Y coordinates of the same holograms when the galvo pair is at a different location. From top to bottom: n=354,364,362 and 338 holograms respectively. Linearity of these X and Y positions indicates that relative distances between holograms are maintained with galvo scanning. The SLM to real space calibration computed on the central FOV can therefore be linearly translated to other mesoscopic FOV.

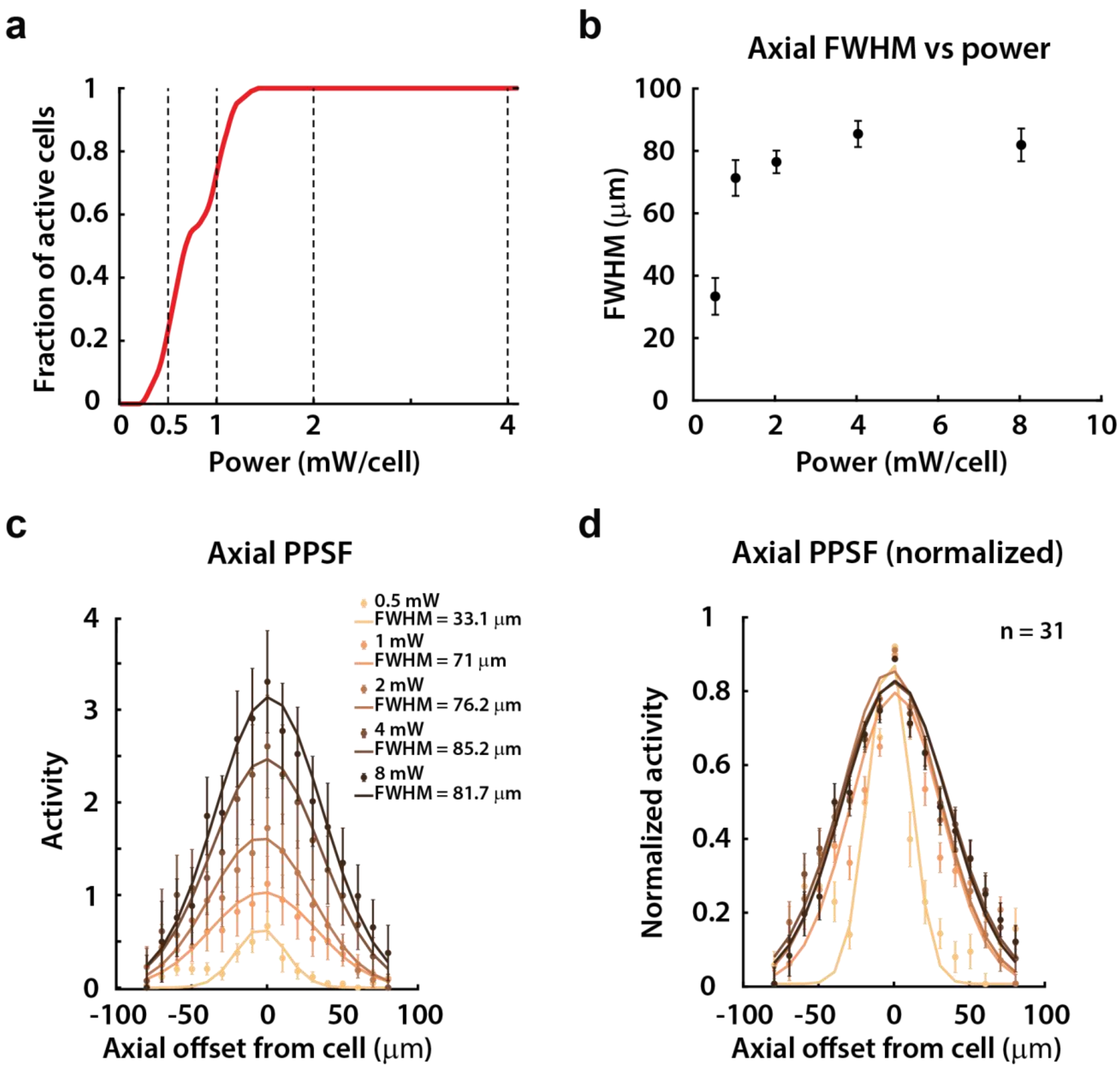

Supplementary Figure 9. PPSFs as a function of power for off-center holography FOV (Galvo offsets: -1V, 0V). a) Cumulative density histogram of the fraction of active cells used in the PPSF experiments (ordinate) that were active above a certain power (abscissa). **b)** Mean +/- s.e.m. axial FWHM across cells as a function of power (n=31 cells). **c)** Axial PPSF profiles for each power condition. The filled circles and error bars are the actual mean +/- s.e.m. across cells (n=31 cells), and the solid traces are weighted gaussian fits. **d)** Normalized axial PPSF profiles for each power condition (n=31 cells), normalized to the

centered hologram location (axial offset = 0). All PPSF experiments were done in CaMK2-tTA; tetO-GCaMP6s mice injected with a pAAV-hSyn-GCaMP6m-p2A-ChRmine-Kv2.1-WPRE virus at discrete locations across visual cortex.

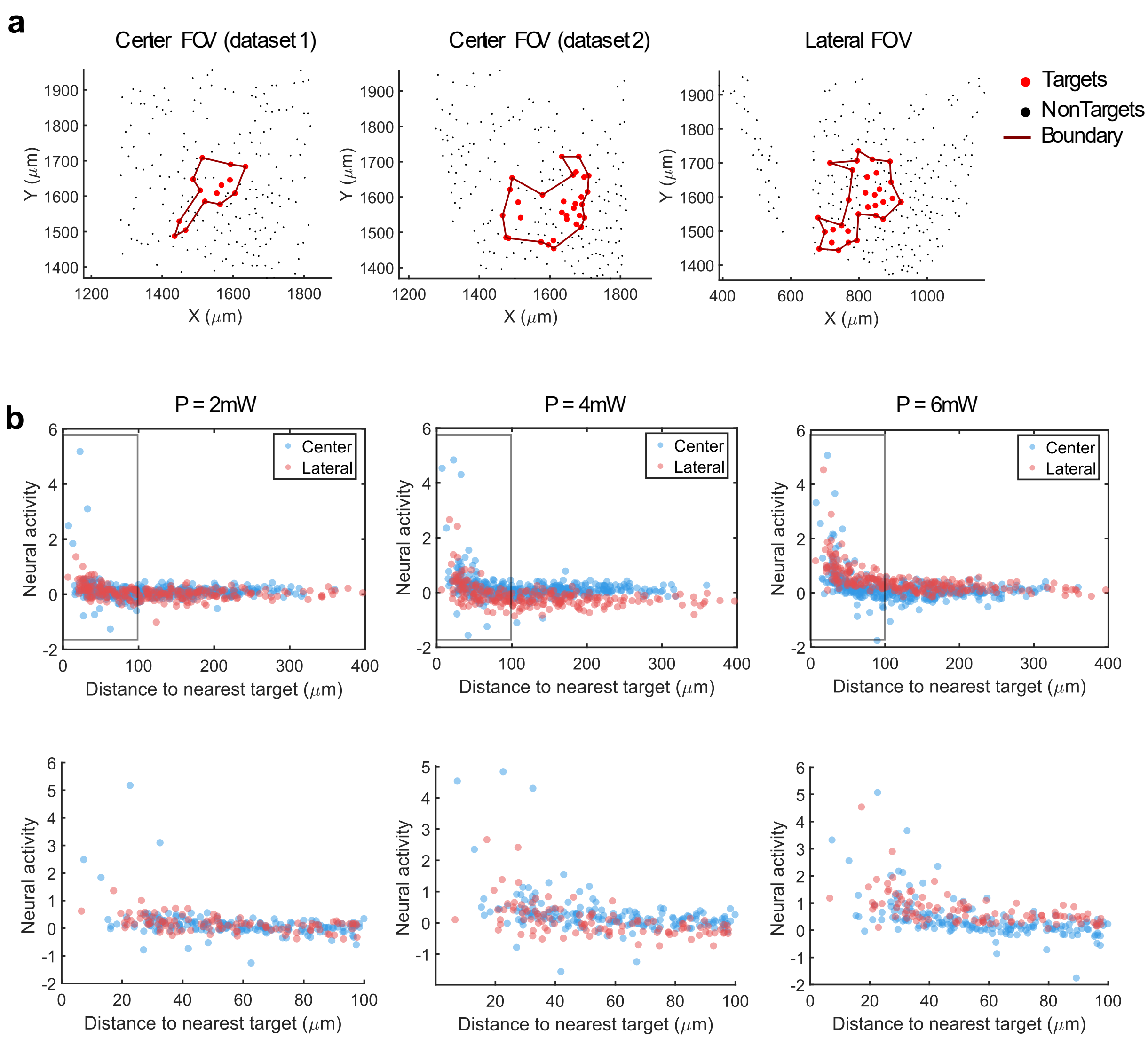

Supplementary Figure 10. Non target responses as a function of distance to target in the central holographic FOV vs. in a lateral holographic FOV. a) Target and non-target spatial locations for center FOV datasets (0V,0V) and lateral FOV dataset (-1V,0V). Because the datasets featured distinct holograms with varying sizes, the comparison was restricted to cells located outside the target boundary (alpha-shape contour, shrink = 0.8). This ensured a fair comparison across conditions, as cells within the target region are subject to multi-cell interactions that differ from one multi-target hologram to another. b) Top: Neural activity as a function of distance to nearest target at different experimental powers. Bottom: Zoom in the 0-100 µm range. Neural activity is calculated as in above PPSF experiments, as deltaF ($F\text{-}F_{baseline}$). Experiments were done in CaMK2-tTA; tetO-GCaMP6s mice injected with a pAAV-hSyn-GCaMP6m-p2A-ChRmine-Kv2.1-WPRE virus.

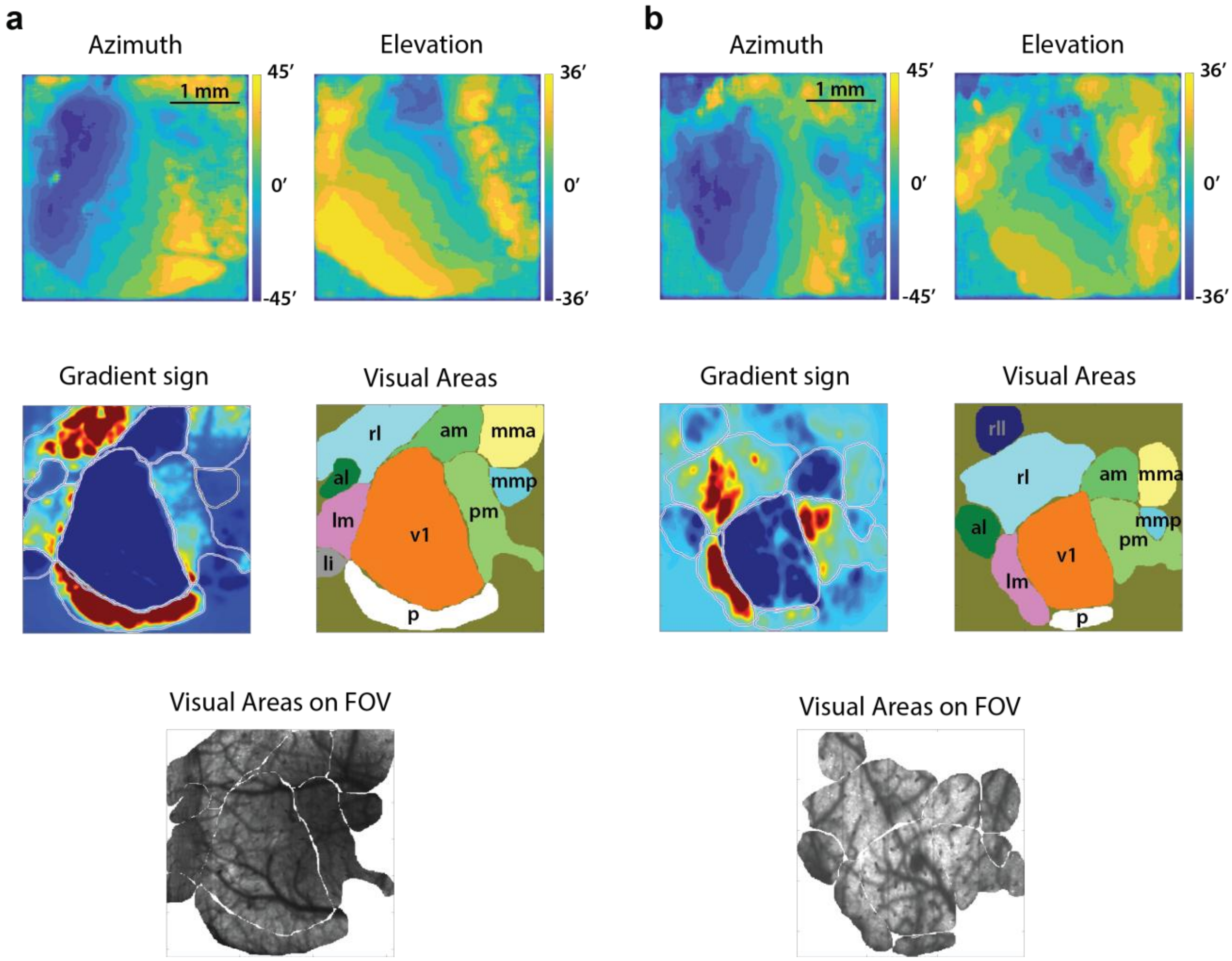

Supplementary Figure 11. Retinotopic identification of visual areas with the 2P-RAM mesoscope. a-b) Two examples of visual areas identified using retinotopy. Top row: Average pixel-wise azimuth (left column) and elevation (right column) preference maps in response to a moving bar stimulus. Middle row: Gradient sign map computed from the retinotopic preferences (left column), and visual areas segmented manually based on transitions in the gradient sign (right column). Bottom row: Segmented visual areas overlaid on the 3 x 3 mm FOV for vasculature-based alignment across sessions.

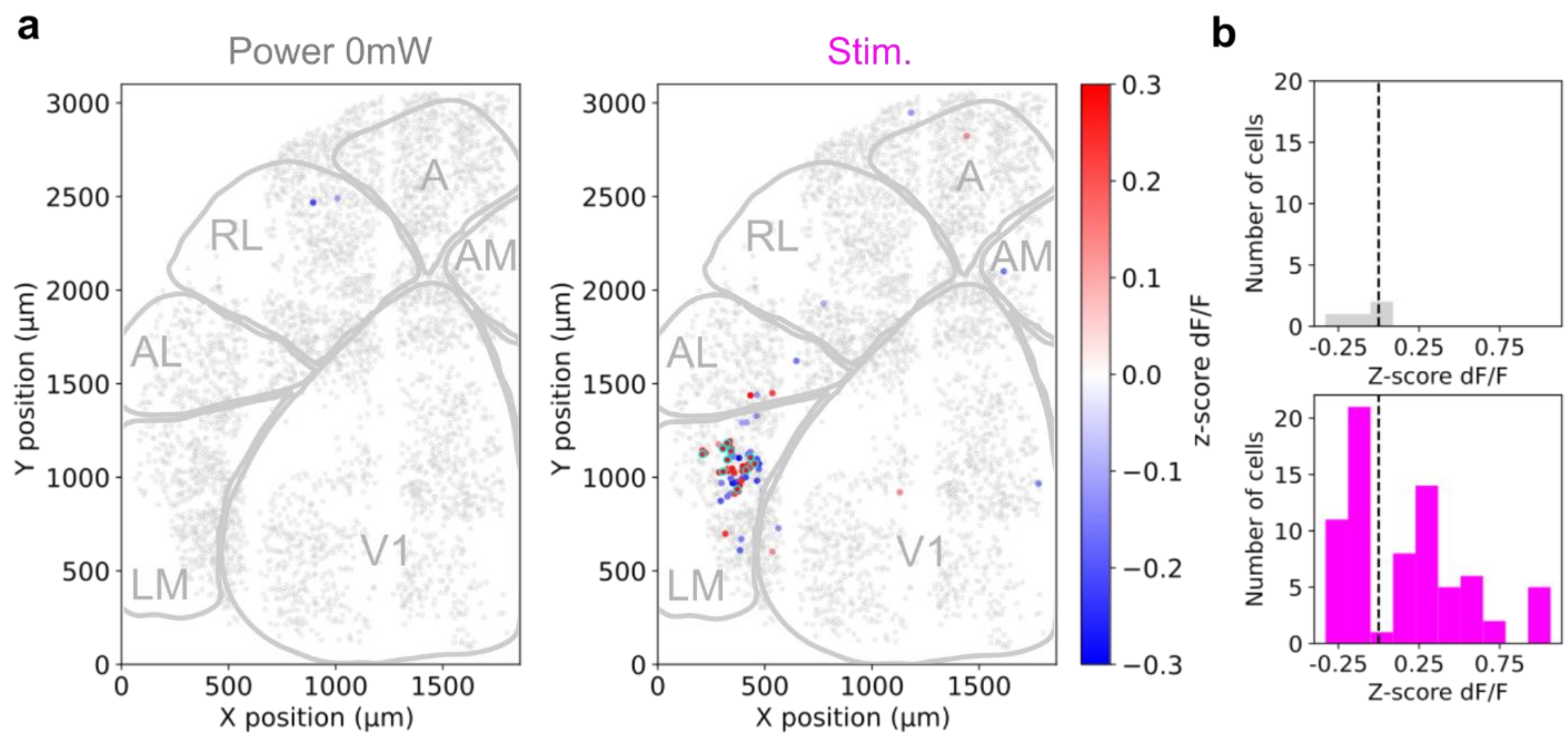

Supplementary Figure 12. Cross-validated influence maps for holographic stimulation and no stimulation control. **a)** Example mesoscale holographic 'influence' maps measured in response to no holographic stimulation (left) and to activation of an ensemble of LM neurons (right). Significantly modulated cells are colored according to the amplitude of their calcium responses. Significantly modulated cells are selected as the intersection of significantly modulated cells (two-sided t-test p-value <0.05) across two random splits of the total number of trials. Non-modulated cells are displayed in gray. Holographic targets are circled in cyan. Mouse: Ai203xVGlutCre. **b)** Distribution of the amplitude of the post-stimulation calcium responses of significantly modulated cells in response to holographic activation (magenta) compared to control trials with no stimulation (grey).

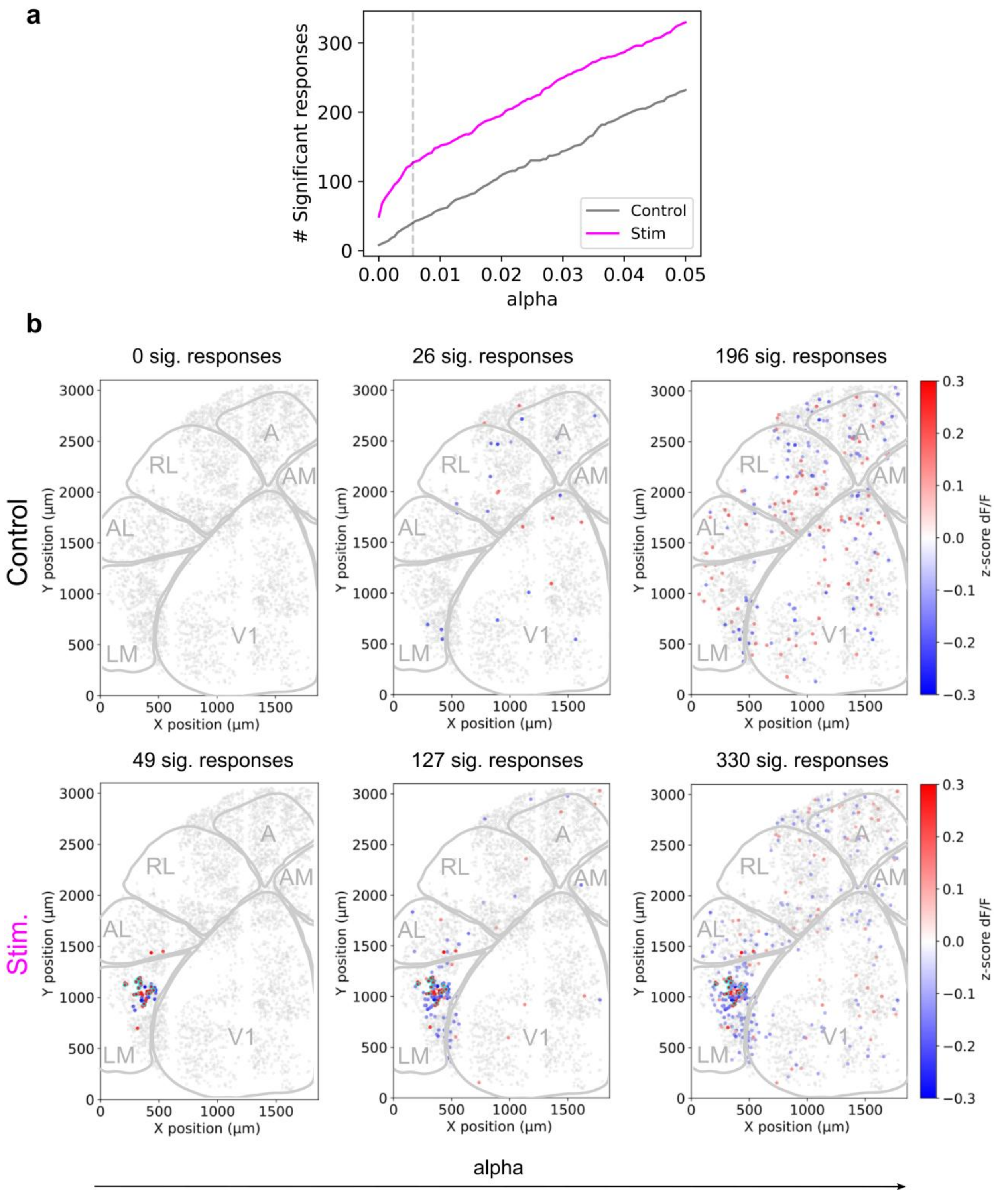

Supplementary Figure 13. Significant modulations and false positive rates. **a)** Number of significantly modulated cells in response to holographic activation and in response to the no stimulation control as a function of the significance level parameter alpha. Holographic stimulation consistently elicits more

significant responses than the false positive rate. Calcium indicator:GCaMP6s, Opsin: ChroME2s. **b)** Maps of significantly modulated cells as a function of the significance level alpha parameter. Alpha parameter from left to right: Bonferroni (1.35×10^{-5}), Inflection point value in a) (5.6×10^{-3}), and 0.05.

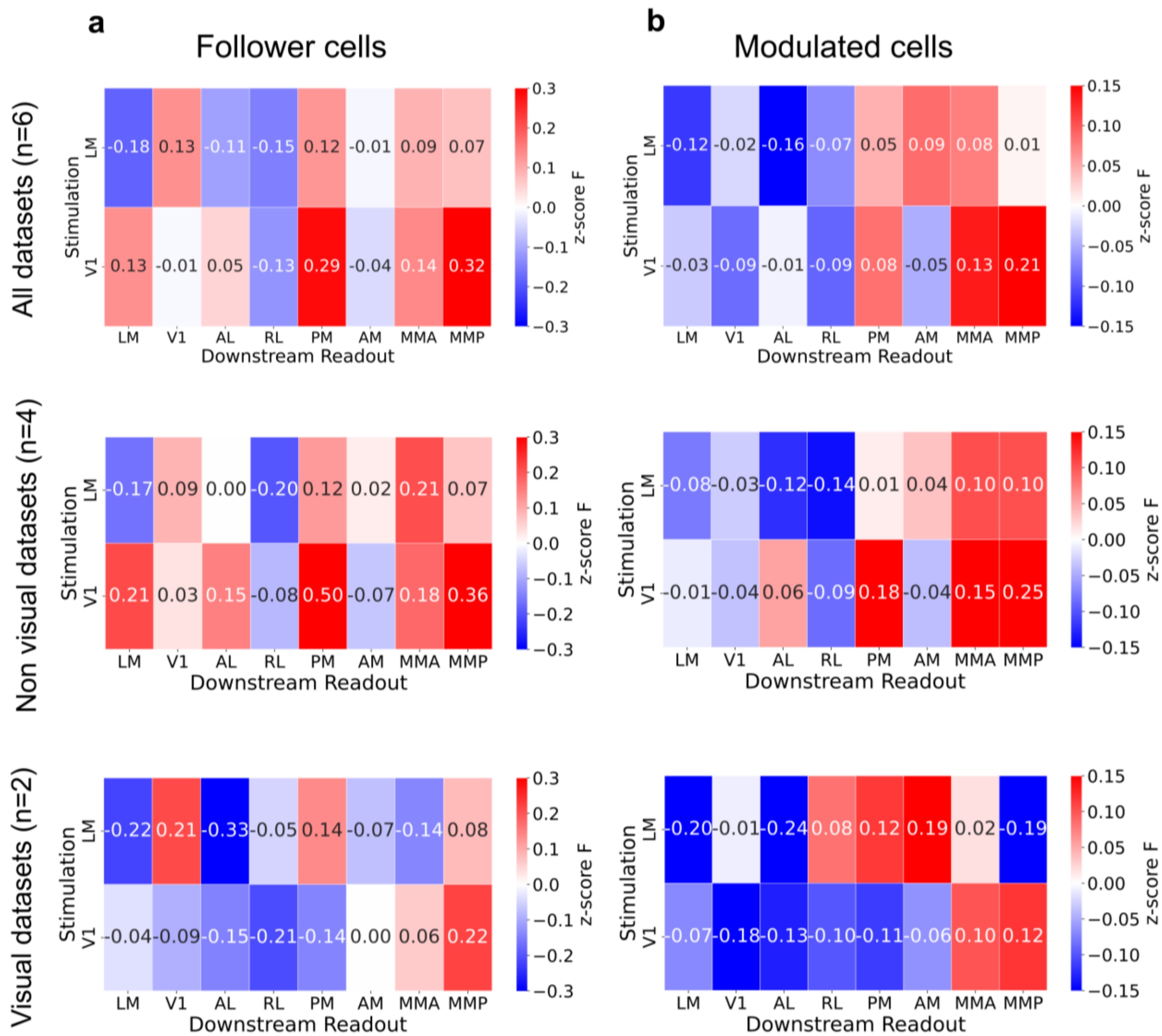

Supplementary Figure 14. Inter-areal functional connectivity maps separated by visual and non-visual conditions. a) Inter-areal maps of net influence computed from post-stimulation responses of follower neurons. Top: All datasets pooled (n=6 sessions, 2mice). Middle: Sessions with holographic stimulation only (n=4 sessions, 2 mice). Bottom: Sessions with concurrent holographic stimulation and visual stimulation (n=2 sessions, 2 mice). b) Inter-areal maps of net influence computed from post-stimulation responses of modulated neurons. Top: All datasets pooled (n=6 sessions, 2mice). Middle: Sessions with holographic stimulation only (n=4 sessions, 2 mice). Bottom: Sessions with concurrent holographic stimulation and visual stimulation (n=2 sessions, 2 mice).

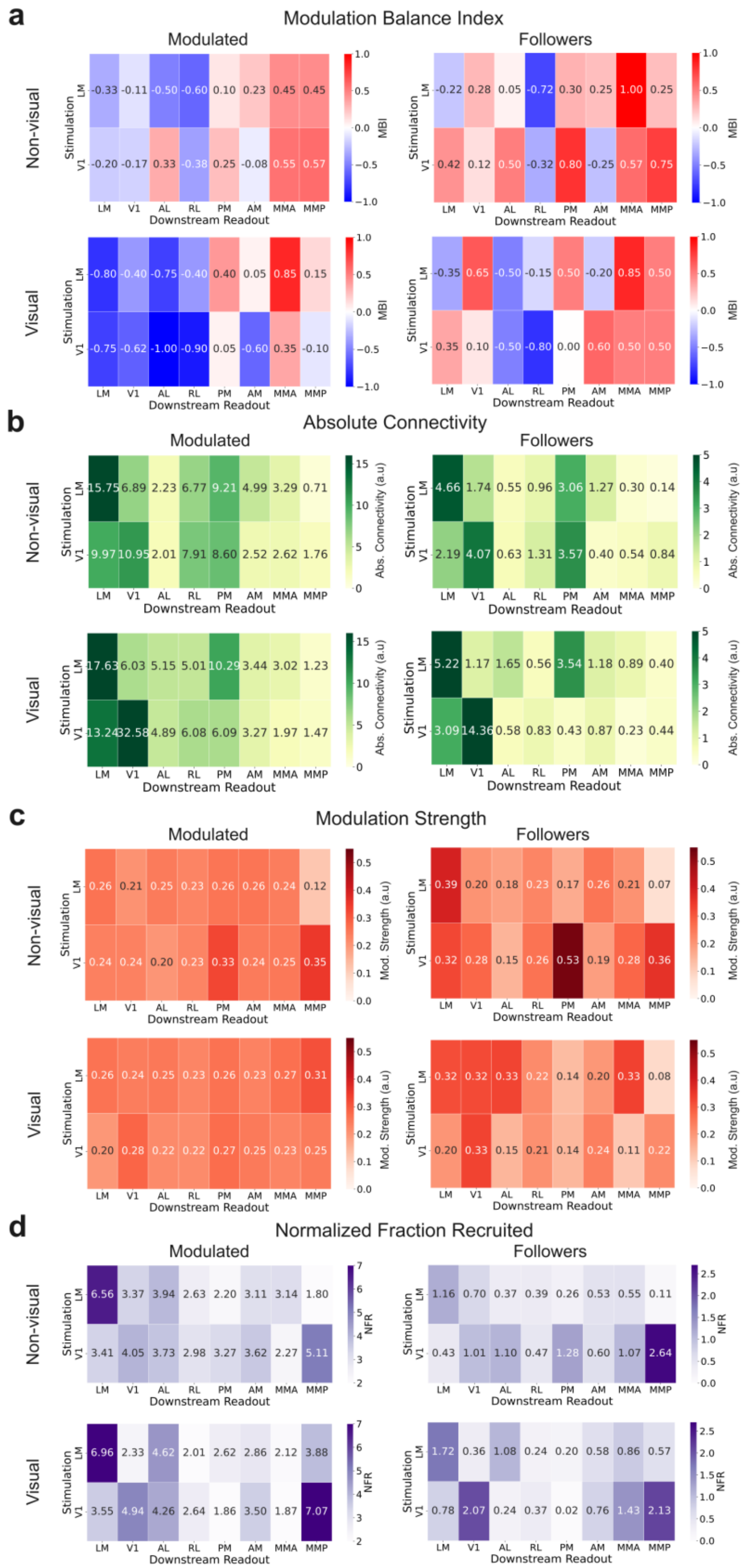
a
Modulation Balance Index
Modulated
Followers
Non-visual
Visual
Stimulation
LM
V1
-0.33 -0.11 -0.50 -0.60 0.10 0.23 0.45 0.45
-0.20 -0.17 0.33 -0.38 0.25 -0.08 0.55 0.57
-0.22 0.28 0.05 -0.72 0.30 0.25 1.00 0.25
0.42 0.12 0.50 -0.32 0.80 -0.25 0.57 0.75
-0.80 -0.40 -0.75 -0.40 0.40 0.05 0.85 0.15
-0.75 -0.62 -1.00 -0.90 0.05 -0.60 0.35 -0.10
-0.35 0.65 -0.50 -0.15 0.50 -0.20 0.85 0.50
0.35 0.10 -0.50 -0.80 0.00 0.60 0.50 0.50
LM V1 AL RL PM AM MMA MMP
Downstream Readout
MBI
b
Absolute Connectivity
Modulated
Followers
15.75 6.89 2.23 6.77 9.21 4.99 3.29 0.71
9.97 10.95 2.01 7.91 8.60 2.52 2.62 1.76
4.66 1.74 0.55 0.96 3.06 1.27 0.30 0.14
2.19 4.07 0.63 1.31 3.57 0.40 0.54 0.84
17.63 6.03 5.15 5.01 10.29 3.44 3.02 1.23
13.24 32.58 4.89 6.08 6.09 3.27 1.97 1.47
5.22 1.17 1.65 0.56 3.54 1.18 0.89 0.40
3.09 14.36 0.58 0.83 0.43 0.87 0.23 0.44
Abs. Connectivity (a.u)
c
Modulation Strength
Modulated
Followers
0.26 0.21 0.25 0.23 0.26 0.26 0.24 0.12
0.24 0.24 0.20 0.23 0.33 0.24 0.25 0.35
0.39 0.20 0.18 0.23 0.17 0.26 0.21 0.07
0.32 0.28 0.15 0.26 0.53 0.19 0.28 0.36
0.26 0.24 0.25 0.23 0.26 0.23 0.27 0.31
0.20 0.28 0.22 0.22 0.27 0.25 0.23 0.25
0.32 0.32 0.33 0.22 0.14 0.20 0.33 0.08
0.20 0.33 0.15 0.21 0.14 0.24 0.11 0.22
Mod. Strength (a.u)
d
Normalized Fraction Recruited
Modulated
Followers
6.56 3.37 3.94 2.63 2.20 3.11 3.14 1.80
3.41 4.05 3.73 2.98 3.27 3.62 2.27 5.11
1.16 0.70 0.37 0.39 0.26 0.53 0.55 0.11
0.43 1.01 1.10 0.47 1.28 0.60 1.07 2.64
6.96 2.33 4.62 2.01 2.62 2.86 2.12 3.88
3.55 4.94 4.26 2.64 1.86 3.50 1.87 7.07
1.72 0.36 1.08 0.24 0.20 0.58 0.86 0.57
0.78 2.07 0.24 0.37 0.02 0.76 1.43 2.13
NFR

Supplementary Figure 15. Inter-areal functional connectivity metrics separated by visual and non-visual conditions. Modulation Balance Index, Absolute Connectivity, Modulation Strength and Normalization Fraction Recruited computed separately for sessions with holographic stimulation only (n=4 sessions, n=2 mice) and for sessions with concurrent holographic and visual stimulation (n=2sessions, n=2mice), shown for both follower and modulated neurons. See Extended Data Fig. 10 for detailed definitions of the connectivity metrics.

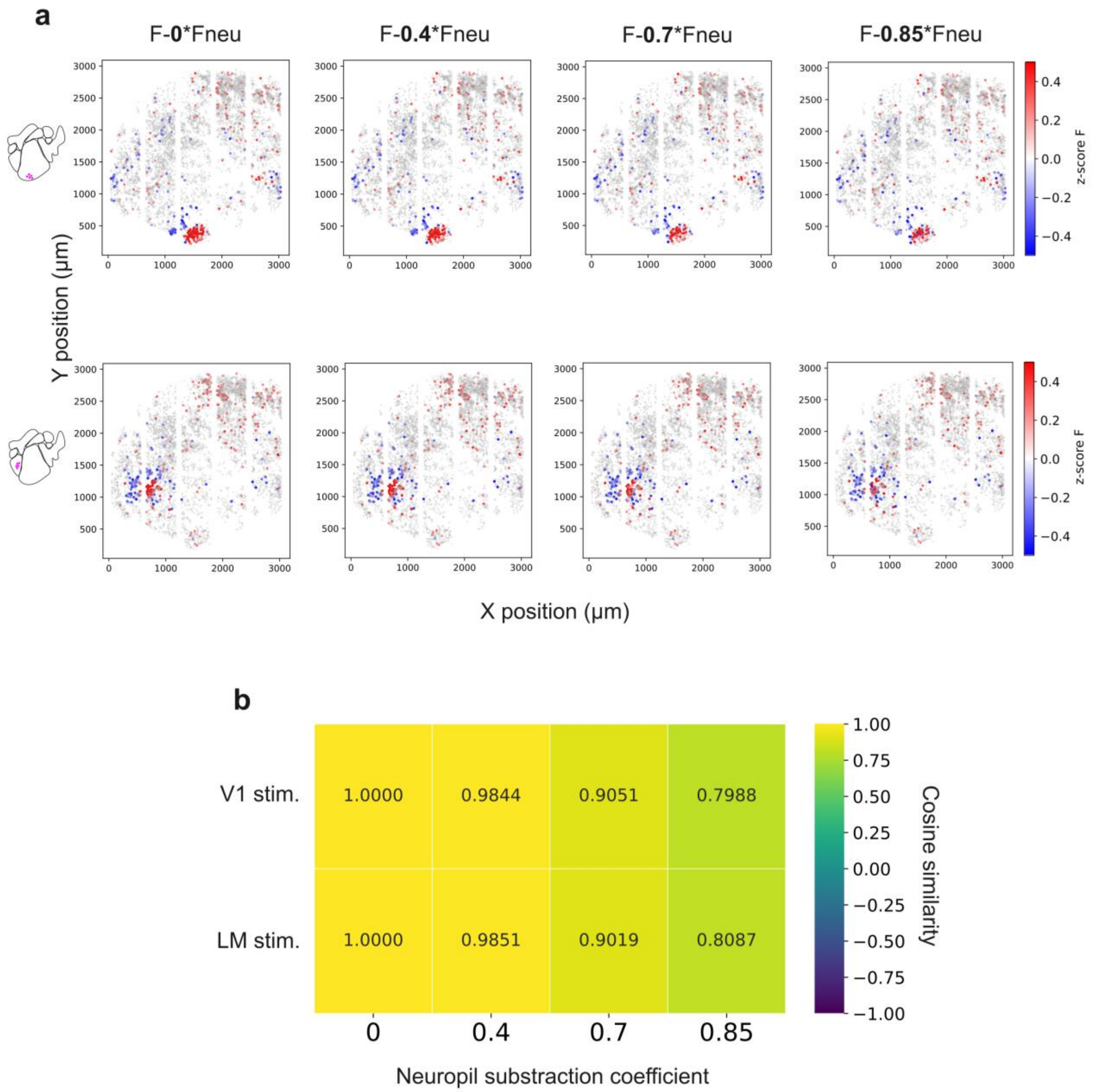

Supplementary Figure 16. Impact of neuropil subtraction on modulated cell responses. a) Example influence maps for photostimulation in V1 (top) and LM (bottom) showing targets and significantly modulated cell responses with different neuropil subtraction coefficients. **b)** Cosine similarity of the population response compared to the uncorrected (no neuropil subtraction) condition.

<u>Supplementary note 1</u>: Assembly and alignment guide

Detailed documentation on mesoscope assembly and alignment is available on the Janelia open wiki https://wiki.janelia.org/wiki/display/mesoscopy/Documentation. We focus below on the specifics for adding a 2P holographic module to the Janelia/Thorlabs mesoscope.

A) <u>Prerequisites</u>:
   Adding a 2P holographic photostimulation path onto the mesoscope required some custom parts and modifications to the original mesoscope parts. Items 1-3 below were designed in collaboration with Thorlabs who manufactured the final parts. All the part designs are available on the CAD file attached. We do however encourage scientists willing to upgrade their system to reach to Thorlabs for assistance with the items 1-3 upgrades.

   1. The third scan relay (scan lens and tube lens assembly) needs to be opened to merge the imaging and photostimulation beams. See CAD file for aperture size and position.

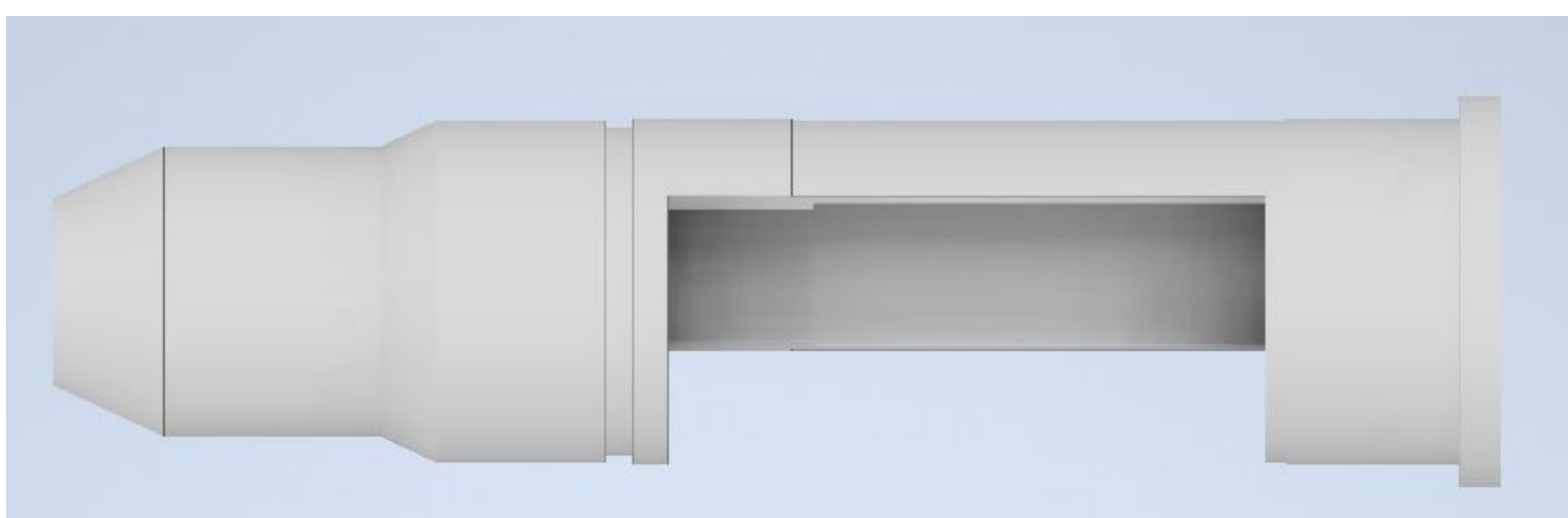

Modified relay mount.

   2. The 2P holographic path is set up on a horizontal custom 33”” × 24”” × 0.5”” aluminum breadboard (Thorlabs) attached to the main motorized mesoscope frame (gantry). The breadboard is attached to the gantry through two custom support clamps in the back (Thorlabs) and one custom L-bracket on the front (Thorlabs). Securing the L-shape bracket in place required drilling 4 ¼”-20 tapped holes on the mesoscope rotation hub.

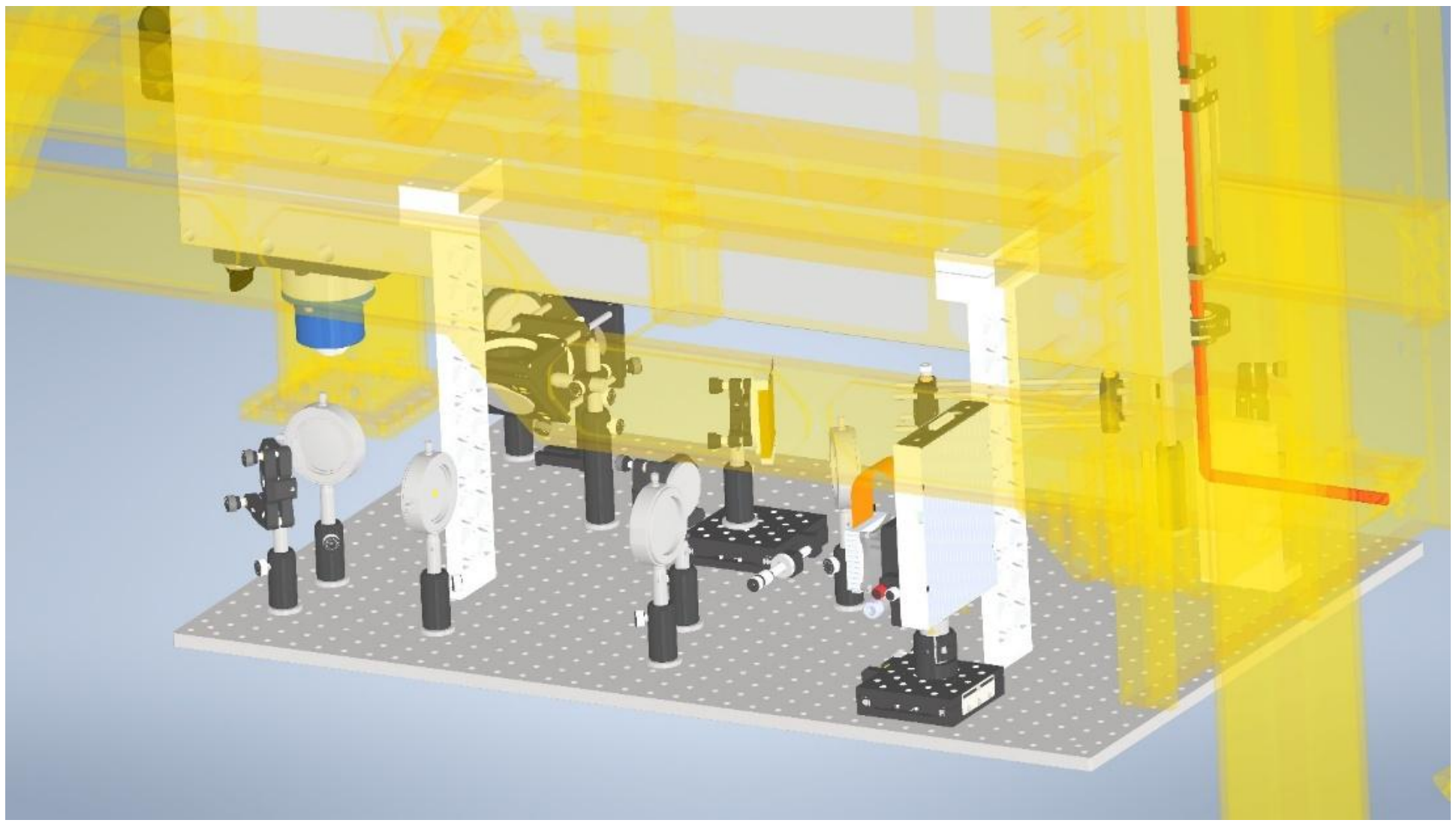

Back support clamps.

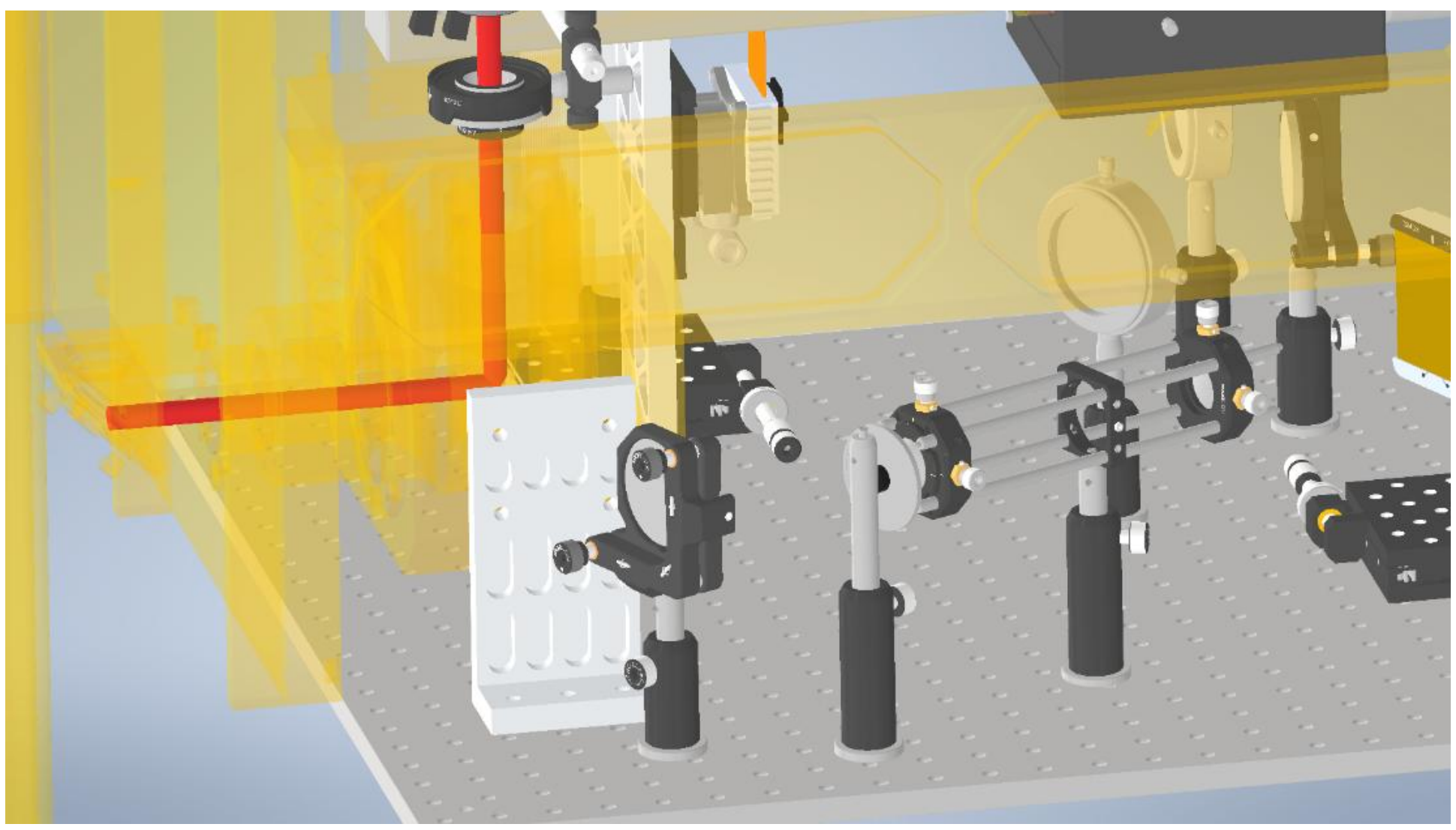

L-bracket attached to the mesoscope rotation hub.

3. A custom threaded bracket (Thorlabs) was used to attach the recombination dichroic D3 onto the vertical mesoscope breadboard.

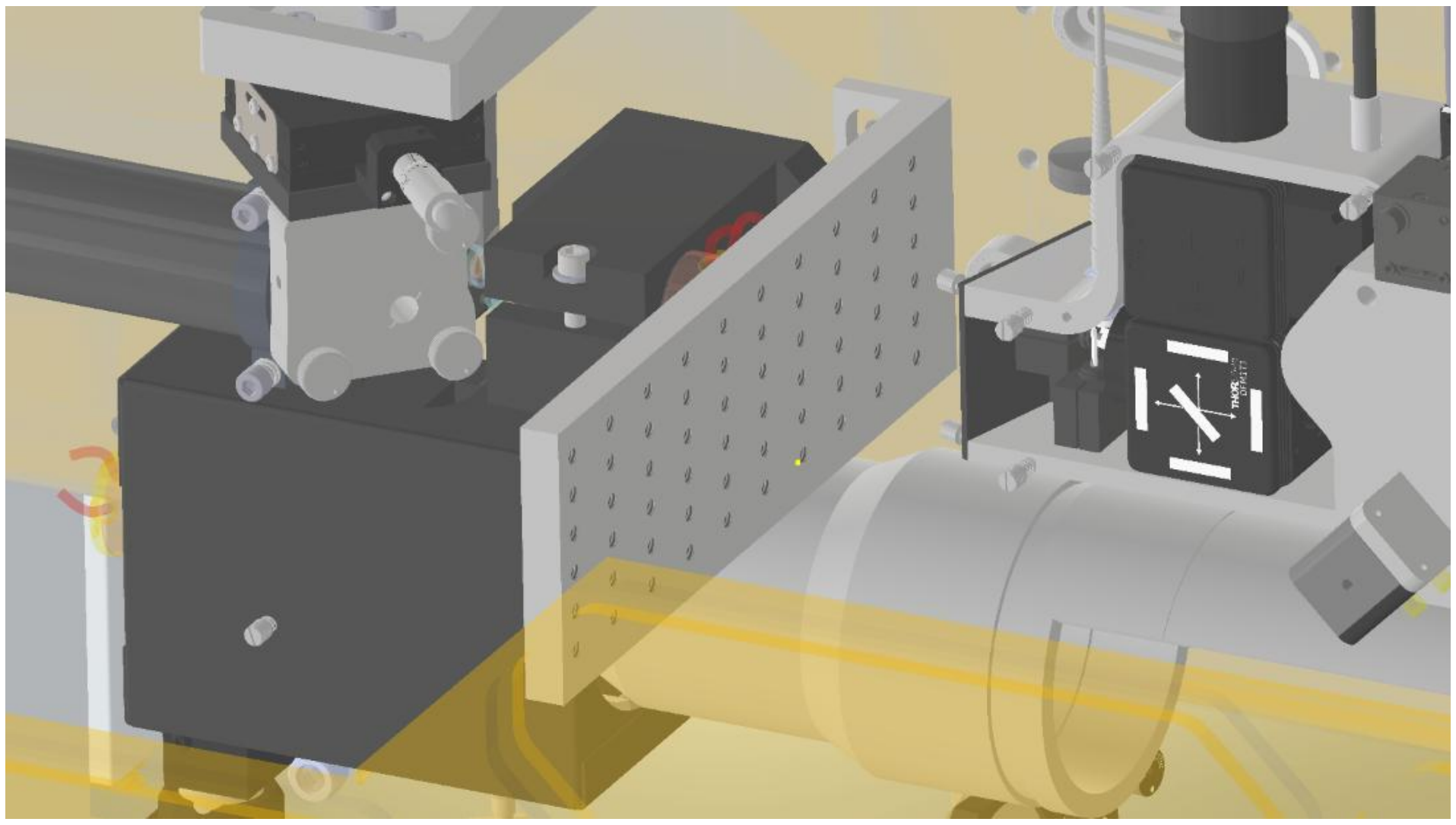

Bracket to attach the recombination dichroic holder to the mesoscope.

4. A custom 3D-printed holder is used to flexibly position the recombination dichroic D3 on the mesoscope scan path. The holder allows flexible positioning across 3 translation directions and one rotation axis and was designed to hold a standard ½" Thorlabs post. The dichroic is secured in place with a FFM1 holder (Thorlabs) secured on a B3C mount (Thorlabs).

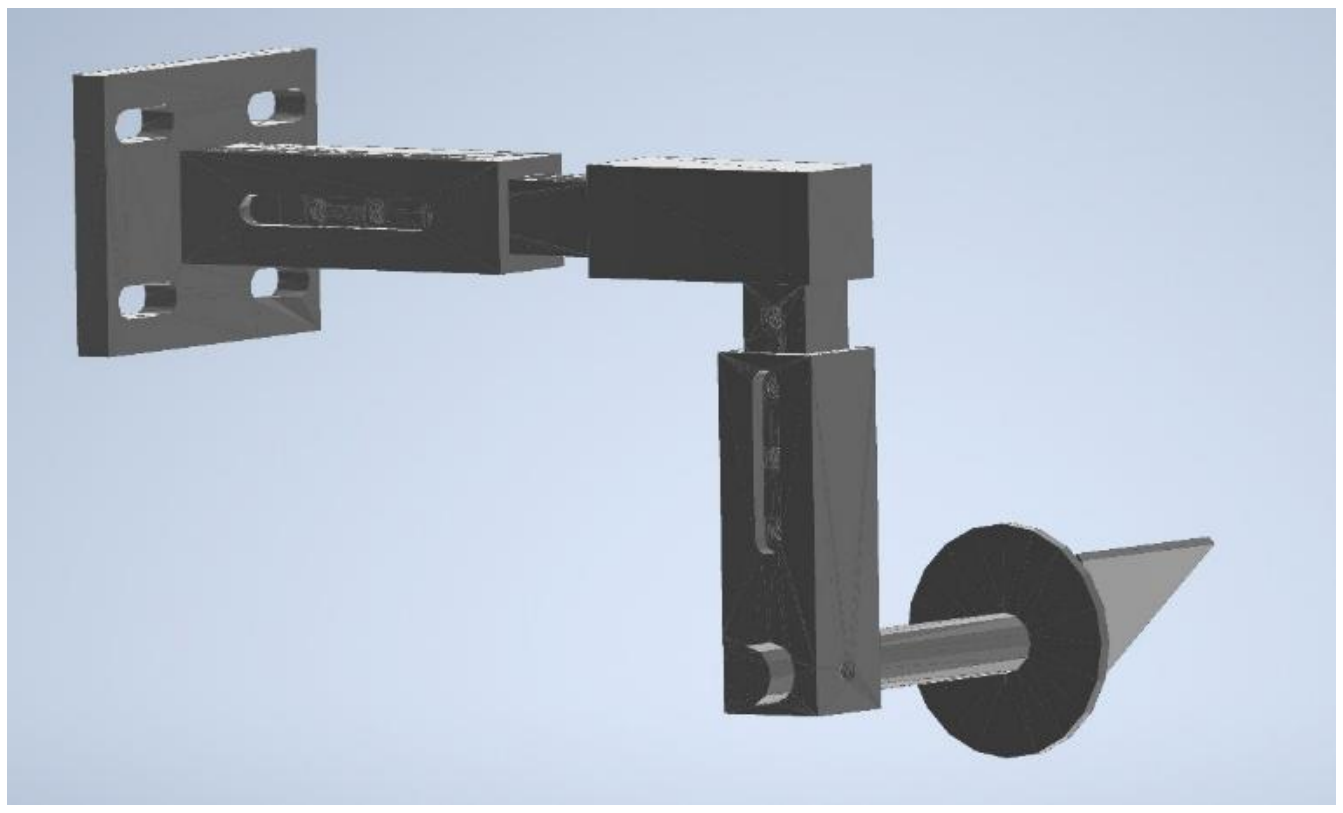

Recombination dichroic holder.

5. To maintain axial alignment, the motorized axial movement of the vertical breadboard relative to the gantry is locked and replaced with an external motorized lab jack (Thorlabs, MLJ150).

B) <u>Assembly and alignment:</u>

1. Once the breadboard is installed, pre-position all breadboard optics to match the 4f requirements using the CAD layout as a guide.

2. Align the imaging beam up until the rotation hub to be centered on each of the gantry periscope mirrors (M1,M2,M3,M4,M5) and perfectly stationary with the gantry's X and Y translation.

3. Remove the last periscope mirror (M6) and place the provided rotation alignment tool. If step 2 is done properly, M5 is enough to steer the beam to be centered and leveled along the rotation axis and to have the beam position invariant with rotation.

4. Inject the stimulation beam onto the periscope so that the beam is co-aligned to the imaging beam i.e centered on all mirrors and invariant with X, Y and rotation. For this step only the D1 dichroic and mirrors external to the gantry periscope can be used. Consider having the recombination dichroic D1 installed on a lateral translation stage to have an additional degree of freedom for co-alignment.

5. Given the natural divergence of the laser beam and the long distances on the mesoscope, the beam should be quite expanded on the rotation alignment tool. To correct for that, we added a 1:1 telescope before the gantry (lenses L01 & L02) to have a more collimated beam impinging on the grating. The lenses of the telescope need to be placed centered on the beam so that the beam remains undeflected. L02 could be placed on a translation stage to allow for easier adjustment of the divergence while maintaining the alignment. Fine adjustments of the imaging/stimulation beam co-alignment can be made after the telescope is installed (again, only with mirrors external to the gantry or with the D1 dichroic).

6. Remove the rotation alignment tool and replace M6 with the D2 dichroic. At this stage, the imaging and stimulation beams can now be aligned independently.

7. Subsequent alignment of the imaging path on the vertical breadboard is now constrained since only D2 can now be used to route the beam straight on the pre-RF vertical axis. Therefore, depending on how the system has been originally set, the whole pre-RF vertical axis might need to be translated horizontally so that the beam can be straight and vertical along that axis.

8. After steps 4 and 5, the stimulation beam should be perfectly leveled impinging on the grating. Adjust the angle of the grating to minimize power lost at higher diffraction orders. Then iterate adjusting the height and position of the relay rail and adjusting the tip/tilt on the grating mount so that ultimately the beam is perfectly centered on L1 and L2.

9. Install the spinning diffuser and adjust the subsequent mirror to stay leveled at the beam height and to hit the SLM injection mirror while being centered and on-axis on the L3 lens.

10. Load a flat phase on the SLM (or simply turn it off). Use the pre-SLM injection mirror and the tip-tilt knobs on the SLM to make the beam centered and leveled on the L4 axis. Having the SLM set on a translation stage can be helpful at this stage since it allows to correct for any clipping issue without changing the angle of the SLM. If the beam is properly centered on the SLM, the beam after L4 should look like a full rectangle beam on an IR card. With subsequent

mirrors, the beam should then be aligned to be centered and leveled on the L5 and L6 axes. Prior to this, the cage rail system holding L6 need to be pre-positioned precisely under the imaging relay aperture so that a 90° beam exiting the cage system is centered on the imaging scan relay vertical axis.

11. Place the recombination dichroic D3 in order to have the stimulation beam centered on the mesoscope objective back aperture. Iterate between the position and angle of D3 and the tip/tilt knob of the last cage mirror to have the beam both centered on the back aperture of the objective and straightly vertical on the objective axis.

All lenses are oriented with the curved face towards the collimated beam to minimize cumulative spherical aberrations.

Supplementary note 2: Hologram axial resolution

The 2P fluorescence signal of a temporally focused gaussian spot at the focus of the objective can be written as:

$$S(r,z,t) = \frac{1}{\tau(z)f} I(r,z,t)^2$$

With τ the pulse width, f the repetition rate, I the intensity profile at the focus, r the radial dimension($r = \sqrt{x^2 + y^2}$) and z the axial dimension, both defined with the center of the gaussian spot as origin. Under the approximation that the linear chirp at the objective back aperture (αΩ, with α a constant proportional to the grating groove density and Ω the FWHM of the pulse frequency spectrum) is significantly smaller than the size of each monochromatic beamlet s ($\sqrt{2ln2}$s the FWHM of each monochromatic beamlet), the pulse width as a function of the axial depth z can be written as[1]:

$$\tau(z) \approx \frac{2\sqrt{2ln2}}{\Omega} \sqrt{1 + \frac{z^2}{z_R^2}}$$

With $z_R = \frac{2f_{obj}^2}{k_0(s^2+\alpha^2\Omega^2)}$, f_{obj} the objective focal length, and k_0 the wavevector of the central frequency of the pulse.

The 2P fluorescence signal can then be written as:

$$S(r,z,t) = \frac{\Omega}{2\sqrt{2ln2} f \sqrt{1 + \frac{z^2}{z_R^2}}} I(r,z,t)^2$$

When injecting into this model numerical values corresponding to a conventional 2P objective (here Olympus 20X 1.0 NA f_{obj} = 9mm and 18mm back aperture size) and values corresponding to the 2P-RAM mesoscope objective (0.6 NA f_{obj} = 21mm, 25mm back aperture size), we were

able to obtain, for a 400 fs pulse width at 1 MHz and at 1.03 µm wavelength, a cut-off threshold at the tail of the distributions corresponding to empirically observed axial hologram sizes: $\sim 30\mu m$ for the 2P-RAM mesoscope and $\sim 20\mu m$ for the conventional 2P microscope[2,3] . The large back aperture of the mesoscope objective seems to partially compensate for the lower NA regarding axial temporal focusing resolution.

Supplementary note 3: Physiological point-spread function

The physiological point-spread-function (PPSF) is the main metric for quantifying single-cell resolution in two-photon optogenetics experiments. It is usually reported as the FWHM (full-width half maximum) of the neuron's response as the optical hologram is digitally displaced along the axial axis. In this manuscript, we use the GCaMP calcium response as a readout for the neuron's response. The effective PPSF can be partially modeled as the convolution of the optical axial point-spread-function with the cell diameter and is affected by several parameters (stimulation power, opsin expression, potency and localization and cell intrinsic properties[4]). We have performed extensive PPSF characterizations in mice expressing the very potent opsin ChRmine (Fig.2, supp Figs. 8,9 and 15) to provide a range of PPSFs to be expected by experimenters using our platform. At photostimulation powers well below saturation, we were able to achieve *in vivo* near single-cell precision with 33-35µm axial FWHMs (supp. Fig. 8 and 15) in both central and off-axis holographic FOVs. At saturating powers, we measured ~70µm axial FWHMs in the central holographic FOV and ~80µm off-axis. These values fall on the higher end but are still within the range of previously reported PPSFs in the field[5,6]. While these high-power conditions were the ones used in the functional connectivity experiments in the present manuscript, it is important to note that for many cells these conditions were on the saturation part of the power curve and the same experiments can be done at lower powers below saturation. Depending on the biological question under investigation, experimenters can choose their photostimulation parameters to optimize the trade-off between larger PPSFs and the number of elicited spikes per neuron for a set stimulation duration. To optimize this trade-off, one can adjust the individual power delivered to each cell (supp Fig 9, see also [7]), at the expense of increasing experimental complexity. Notably, earlier PPSF measurements in Ai203×Vglut-Cre transgenic mice revealed an axial PPSF of 49 µm, well within the range reported for ChRmine-based viral expression. Lateral PPSF measurements ranged from 24 µm to 38 µm across powers and opsins, with the upper end corresponding to saturating photostimulation powers with virally injected ChRmine. (Fig. 2, supp. Fig. 8).

Supplementary note 4: Functional connectivity statistics

(i) Significance of differences across areas: The analyses presented here are intended solely to establish that the connectivity patterns measured by the holographic mesoscope reflect reproducible non-random structure rather than measurement noise. As this manuscript is a technical report, we do not aim to provide a biological interpretation of inter-areal functional connectivity, which would warrant its own independent study. Instead, the purpose is to demonstrate that the method can reliably resolve structure mesoscale connectivity patterns,

thereby motivating future studies that will be required to address biological mechanisms and functional significance. For each row (LM stim. And V1 stim.), we performed a nonparametric permutation test in which area labels were shuffled within each session (10 000 iterations) to create a null distribution. For LM stimulation, the initial repeated-measure ANOVA yielded $F(7,35)=2.69$. This F value was greater than 97.3% of shuffled datasets ($p=0.027$), confirming that the LM functional connectivity map contains a statistically reliable area-specific structure. The same was true for the V1 connectivity map ($p=0.0075$, observed F greater than 99.25% of shuffled datasets). P-values for the two a priori omnibus tests were corrected for multiple comparisons using the Holm–Bonferroni procedure, and remained significant (supp. Table 3).

We have performed the above analysis for both rows (LM stim. And V1 stim.), and for all connectivity metrics presented in this manuscript. The results are summarized in Supp. Table 3. The summary is that Net influence, Modulation Balance Index and Absolute Connectivity maps all show reliable significant cross-area patterns (except for LM stimulation net influence computed with modulated cells, top row Extended data Fig. 8b). By contrast, metrics such as Modulation Strength and Normalized Fraction Recruited for the most part did not reveal significant differences across areas. This likely reflects the fact that modulation strength is generally comparable across downstream areas, with only rare cases of sparse but strong modulation. In the case of NFR, the divisive normalization likely obscures cross-area differences rather than highlighting them.

The above analyses have been done on the pooled dataset (N=6 sessions, 2 mice) with N=4 sessions, 2 mice without visual stimulation and n= 2 mice, 2 sessions with concurrent visual stimulation. For completeness, we present the maps and connectivity metrics computed separately for both conditions in Supp. Figs. 15 and 16. (ii) <u>Follower vs. Modulated Cells</u>:

Our approach to distinguish Follower from Modulated cells is conceptually similar to adjusting the significance alpha threshold. However, setting an alpha threshold is inherently subjective. Because of the intrinsic variability of neural activity and the very large number cells, applying a fully multiple-comparison–corrected threshold would be overly conservative in this context, effectively eliminating genuine follower responses. While $\alpha = 0.05$ is a conventional baseline, we aimed to adopt a more stringent and biologically meaningful criterion: requiring cells to show significant modulation across two independent trial splits. We recognize that any threshold involves a degree of subjectivity, but we believe this approach provides a principled way to identify the most reliable followers.

We have compared Followers and Modulated connectivity maps to test whether they differ more than expected by chance. A caveat of such a test is that the two distributions are not independent, given that followers are for the most part a subset of modulated cells. We first performed a global permutation test on session-wise follower vs. modulated LM stimulation maps which revealed no difference (mean distance = 0.457, permutation $p = 1.0$): 100% of permutations produced differences at least as large as the one observed between the follower maps and the modulated maps. We followed up with paired per-area tests which only showed

a weak trend for V1 (p=0.051). For V1 stimulation maps, the global permutation test also didn't reveal any difference (mean distance: 0.53, permutation p = 1). Follow-up paired per-area t-tests showed significance only for LM (p = 0.04) and MMP (p=3.3 × 10-5). These results show that follower and modulated net influence maps are overall not statistically distinguishable at our current sample size, with only minor area-specific differences.

The distinction between follower and modulated maps remains biologically meaningful, even if the maps are not statistically separable. Follower cells represent strong functional connections, defined both by their high reliability and by the magnitude of their responses. By construction, the follower selection identifies the most reliable connections. Moreover, we show that follower cells exhibit stronger response amplitudes than modulated non-followers (Extended data Fig. 8c).

Supplementary table 1 – List of optical components (holography path)

L01	Achromatic doublet f = 50 mm, AC254-50-B. **Thorlabs, USA**
L02	Achromatic doublet f = 50 mm, AC254-50-B. **Thorlabs, USA**
D1	Shortpass Dichroic Mirror, DMSP1000R. **Thorlabs, USA**
D1 (RAM 3D-SHOT)	1040 nm StopLine notch laser dichroic, 50 × 50 mm. **AVR Optics USA**
D2	980 nm laser BrightLine single-edge dichroic beamsplitter, 25 mm diameter. **AVR Optics, USA**
G	Diffraction Grating, Plane Ruled 50 × 50 mm, 1000nm, 600Gr/mm, 33010FL01-520R. **Newport Corporation, USA**
L1	Achromatic doublet f = 125 mm, AC254-125-B-LM. **Thorlabs, USA**
L2	Aspheric Lens f = 20mm, AL2520H-B. **Thorlabs, USA**.
D	0.5° Diffusing Angle 50 mm, #47-989. **Edmund Optics, USA**
L3	Achromatic doublet f = 500 mm, ACT508-500-B. **Thorlabs, USA**
SLM	1920 × 1152 Phase Series Spatial Light Modulator, **Medowlark Optics, USA***
L4	Achromatic doublet f = 150 mm, AC508-150-B. **Thorlabs, USA**
L5	Achromatic doublet f = 300 mm, ACT508-300-B. **Thorlabs, USA**
BRU/L1 (RAM 3D-SHOT)	Achromatic doublet f = 150 mm, AC508-150-B. **Thorlabs, USA**
BRU/L2 (RAM 3D-SHOT)	N-BK7 Plano-Concave lens f = -75mm, LC1315. **Thorlabs, USA.**
XY Galvos (RAM 3D-SHOT)	Beam Galvo system QS30XY-AG. **Thorlabs, USA**
L6	Achromatic doublet f = 200 mm, ACT508-200-B. **Thorlabs, USA**
L6 (RAM 3D-SHOT)	F-theta scan lens FTH100-1064. Thorlabs, USA.
TL	Custom Tube Lens, f=236mm. **Jenoptik USA**<br>Optical design file can be found at: https://wikis.janelia.org/display/mesoscopy/Optical+design+files. See also Sofroniew et al., 2016.

Objective	Custom Objective, f=21mm. **Jenoptik USA**<br>Optical design file can be found at:<br>https://wikis.janelia.org/display/mesoscopy/Optical+design+files. See also Sofroniew et al., 2016.
D3	1040 nm StopLine notch laser dichroic, 50 × 50 mm. **AVR Optics USA**
D3 (RAM 3D-SHOT)	1040 nm StopLine notch laser dichroic, 75 × 75 mm. **AVR Optics USA**
D4	Shortpass Dichroic Mirror, DMSP1000R. **Thorlabs, USA**
Mirrors	Broadband dielectric mirrors, BB2-E03. **Thorlabs, USA**

Refer to CAD file for optomechanical parts.

*: This part has unfortunately been discontinued and is no longer sold by the vendor. We briefly discuss below two alternative options from the same vendor:

1) 1920 x 1200 Spatial Light Modulator, Meadowlark Optics, 8µm pixel pitch. This system will enable a replication of the optical performances presented in this paper without any changes in the optics, with a slightly larger accessible holographic FOV. This device is however slower than the device used in the manuscript. All experiments presented in the manuscript can be replicated with this device, at the exception of experiments in Fig.6 which require high-refresh rate devices (>300Hz).

2) 1024x1024 Spatial Light Modulator, Meadowlark Optics, 17µm pixel pitch. With the current optical design, this device can enable similar axial resolution performances, at the expense of a reduced holographic FOV. Adjustments on the relay lenses can be made to adjust the resolution/FOV trade-off, but having simultaneously the same resolution and FOV specs as presented in the manuscript will be challenging. This device has however a very high refresh rate (>500Hz). This device can therefore be used in the random-access 3D-SHOT configuration. Relay optics can be optimized for resolution and the limited FOV can be compensated for by the galvanometer system.

<u>Supplementary table 2</u> – Groupe delay dispersion estimation (holography path)

	Part #	Material	Thickness (mm)	GVD at 1.03 µm (fs^2/mm)	GDD at 1.03 µm (fs^2)
L01	AC254-50-B	N-LAK22	7.5	43.78	328.35
		N-SF6HT	1.8	241.67	435.006
L02	AC254-50-B	N-LAK22	7.5	43.78	328.35
		N-SF6HT	1.8	241.67	435.006
L1	AC254-125-B	N-BK7	6	25.123	150.738
		N-SF8	6	88.483	530.898
L2	AL2520H-B	S-LAH64	7.6	63.455	482.258
L3	ACT508-500-B	N-SK2	6	41.15	246.9
		N-SF5	6	155.86	935.16
L4	AC508-150-B	N-LAK22	8.2	43.78	358.996
		N-SF6HT	5	241.67	1208.35
L5	ACT508-300-B	N-SK2	8	41.15	329.2
		N-SF11	6	125.35	752.1

L6	ACT508-200-B	N-SK2	8.2	41.15	337.43
		N-SF57	5	271.33	1356.65
Tube lens	Jenoptiks/Thorlabs				1.20E+03
Objective	Jenoptiks/Thorlabs				5775

The total group delay dispersion (GDD) introduced by the holographic path is estimated at 15190 fs^2.

Supplementary table 3 – Functional connectivity summary statistics

Metric	Cell population	Photostim. Area	Permutation p	Corrected Permutation p (Holm-Bonferroni)	Significant	Figure
Net influence	Followers	LM	0.0268	0.0268	True	5f, ExtData.8a
Net influence	Followers	V1	0.0075	0.0150	True	5f, ExtData.8a
Net influence	Modulated	LM	0.1117	0.1117	False	ExtData.8b
Net influence	Modulated	V1	0.0006	0.0012	True	ExtData.8b
MBI	Followers	LM	0.0029	0.0058	True	ExtData.10a
MBI	Followers	V1	0.0262	0.0262	True	ExtData.10a
MBI	Modulated	LM	0.0007	0.0014	True	ExtData.10a
MBI	Modulated	V1	0.0053	0.0053	True	ExtData.10a
Absolute connectivity	Followers	LM	0.0117	0.0117	True	ExtData.10b
Absolute connectivity	Followers	V1	0.0012	0.0024	True	ExtData.10b
Absolute connectivity	Modulated	LM	0.0068	0.0068	True	ExtData.10b
Absolute connectivity	Modulated	V1	0.0009	0.0018	True	ExtData.10b
Modulation Strength	Followers	LM	0.0886	0.1772	False	ExtData.10c
Modulation Strength	Followers	V1	0.4191	0.4191	False	ExtData.10c
Modulation Strength	Modulated	LM	0.4994	0.4994	False	ExtData.10c
Modulation Strength	Modulated	V1	0.0218	0.0436	True	ExtData.10c

Normalized Fraction Recruited	Followers	LM	0.0758	0.1516	False	ExtData.10d
Normalized Fraction Recruited	Followers	V1	0.1001	0.1516	False	ExtData.10d
Normalized Fraction Recruited	Modulated	LM	0.0704	0.1408	False	ExtData.10d
Normalized Fraction Recruited	Modulated	V1	0.1392	0.1408	False	ExtData.10d

Supplementary references

[1] Papagiakoumou, E., Ronzitti, E., & Emiliani, V. (2020). Scanless two-photon excitation with temporal focusing. *Nature Methods*, *17*(6), 571-581.
[2] Pégard, N. C., Mardinly, A. R., Oldenburg, I. A., Sridharan, S., Waller, L., & Adesnik, H. (2017). Three-dimensional scanless holographic optogenetics with temporal focusing (3D-SHOT). *Nature communications*, *8*(1), 1228.
[3] Mardinly, A. R., Oldenburg, I. A., Pégard, N. C., Sridharan, S., Lyall, E. H., Chesnov, K., ... & Adesnik, H. (2018). Precise multimodal optical control of neural ensemble activity. *Nature neuroscience*, *21*(6), 881-893.
[4] Lees, R. M., Pichler, B., & Packer, A. M. (2024). Contribution of optical resolution to the spatial precision of two-photon optogenetic photostimulation in vivo. *Neurophotonics*, *11*(1), 015006-015006.
[5] Daie, K., Svoboda, K., & Druckmann, S. (2021). Targeted photostimulation uncovers circuit motifs supporting short-term memory. *Nature neuroscience*, *24*(2), 259-265.
[6] Fişek, M., Herrmann, D., Egea-Weiss, A., Cloves, M., Bauer, L., Lee, T. Y., ... & Häusser, M. (2023). Cortico-cortical feedback engages active dendrites in visual cortex. *Nature*, *617*(7962), 769-776.
[7] Bounds, H. A., Sadahiro, M., Hendricks, W. D., Gajowa, M., Gopakumar, K., Quintana, D., ... & Adesnik, H. (2023). All-optical recreation of naturalistic neural activity with a multifunctional transgenic reporter mouse. *Cell reports*, *42*(8).
